# BDCA1^+^ cDC2s, BDCA2^+^ pDCs and BDCA3^+^ cDC1s reveal distinct pathophysiologic features and impact on clinical outcomes in melanoma patients

**DOI:** 10.1002/cti2.1190

**Published:** 2020-11-24

**Authors:** Eleonora Sosa Cuevas, Laurissa Ouaguia, Stephane Mouret, Julie Charles, Florence De Fraipont, Olivier Manches, Jenny Valladeau‐Guilemond, Nathalie Bendriss‐Vermare, Laurence Chaperot, Caroline Aspord

**Affiliations:** ^1^ Institute for Advanced Biosciences, Immunobiology and Immunotherapy in Chronic Diseases Inserm U 1209 CNRS UMR 5309 Université Grenoble Alpes Grenoble 38000 France; ^2^ R&D Laboratory Etablissement Français du Sang Auvergne‐Rhône‐Alpes Grenoble 38000 France; ^3^ Dermatology clinic Grenoble University Hospital Grenoble F‐38043 France; ^4^ Medical Unit of Molecular genetic (hereditary diseases and oncology) Grenoble University Hospital Grenoble F‐38043 France; ^5^ INSERM 1052 CNRS 5286 Centre Léon Bérard Centre de Recherche en Cancérologie de Lyon Université Claude Bernard Lyon 1 Univ Lyon Lyon 69373 France

**Keywords:** cDC1, cDC2, immune subversion, melanoma, pDC, prognosis factor

## Abstract

**Objectives:**

Dendritic cells play a pivotal but still enigmatic role in the control of tumor development. Composed of specialised subsets (cDC1s, cDC2s, pDCs), DCs are critical in triggering and shaping antitumor immune responses. Yet, tumors exploit plasticity of DCs to subvert their functions and escape from immune control. This challenging controversy prompted us to explore the pathophysiological role of cDCs and pDCs in melanoma, where their precise and coordinated involvement remains to be deciphered.

**Methods:**

We investigated in melanoma patients the phenotypic and functional features of circulating and tumor‐infiltrating BDCA1^+^ cDC2s, BDCA2^+^ pDCs and BDCA3^+^ cDC1s and assessed their clinical impact.

**Results:**

Principal component analyses (PCA) based on phenotypic or functional parameters of DC subsets revealed intra‐group clustering, highlighting specific features of DCs in blood and tumor infiltrate of patients compared to healthy donors. DC subsets exhibited perturbed frequencies in the circulation and actively infiltrated the tumor site, while harbouring a higher activation status. Whereas cDC2s and pDCs displayed an altered functionality in response to TLR triggering, circulating and tumor‐infiltrating cDC1s preserved potent competences associated with improved prognosis. Notably, the proportion of circulating cDC1s predicted the clinical outcome of melanoma patients.

**Conclusion:**

Such understanding uncovers critical and distinct impact of each DC subset on clinical outcomes and unveils fine‐tuning of interconnections between DCs in melanoma. Elucidating the mechanisms of DC subversion by tumors could help designing new therapeutic strategies exploiting the potentialities of these powerful immune players and their cross‐talks, while counteracting their skewing by tumors, to achieve immune control and clinical success.

## Graphical abstract

We explored the phenotypic and functional features of circulating and tumor‐infiltrating cDC2s, pDCs and cDC1s in melanoma patients together with their clinical impact. Our study reveals critical and distinct impact of each DC subset on melanoma progression and brings new insights into the pathophysiology of cDC2s, pDCs and cDC1s in melanoma and their prognostic impact on patients’ clinical outcome, allowing a better understanding of melanoma escape from immune‐surveillance. Elucidating the mechanisms of DC subversion by tumors could help designing new therapeutic strategies exploiting the potentialities of these potent immune players and their cross‐talks, while counteracting their skewing by tumors to improve clinical outcomes.

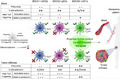

## Introduction

Subversion of immunity by tumors is a hallmark of cancer and a crucial step for their development.^1^ Despite recent improvements in cancer treatment, using targeted therapies^2^ or immunomodulatory strategies,^3^, ^4^, ^5^ long‐term control of the tumor still remains a challenge, especially in melanoma. The infiltration by specific innate and adaptive immune cells has been linked to good clinical outcomes and is correlated with responsiveness to immunotherapies.^6^ These findings point out the essential role of immune cells in the control of tumor progression and motivate the further elucidation of the mechanisms of tumor‐induced immune subversion. A better understanding of the tumor evasion from immune control is crucial to design new therapeutic strategies and to potentiate existing immunotherapies to achieve better clinical success.

Dendritic cells (DCs) are strategic immune cells that connect innate and adaptive immunity and are critical for triggering and shaping immune responses.^7^ DCs recognise pathogen‐ but also damage‐associated molecular patterns (PAMPs and DAMPs, respectively) through their pattern recognition receptors (PRRs), resulting in DC activation and subsequent triggering of anti‐viral/antitumor immunity due to their unique ability to uptake antigens, perform cross‐presentation, and activate antigen‐specific adaptive immune responses.^8^ There are three functionally specialised main DC subsets in human peripheral blood and lymphoid tissues: conventional DCs (cDCs) segregated into type 2 cDCs (BDCA1/CD1c+ DCs, cDC2s) and type 1 cDCs (BDCA3/CD141^+^ DCs, cDC1s), and plasmacytoid DCs (BDCA2^+^ DCs, pDCs).^9^, ^10^ They differ in surface marker expression, localisation, migratory capacity, Toll‐like receptor (TLR) expression, antigen processing and presentation capacities, and cytokine secretion^11^ allowing them to complement each other and induce appropriate immune responses after danger recognition. cDC2s, the main population of DCs in peripheral blood and lymphoid organs, display TLR4 and TLR8 allowing them to recognise various danger signals and are the main producers of interleukin‐12p70 (IL‐12).^12^ cDC2s specialise in MHC class II‐restricted antigen presentation initiating CD4^+^ T‐cell responses. pDCs are experts in type I interferon (IFN) production after TLR7/TLR9 stimulation, important for anti‐viral immune responses^13^ but also crucial for antitumor immunity through their pleiotropic immunomodulatory function.^14^, ^15^ pDCs can lead to antitumor responses through antigen cross‐presentation^16^ or by secreting pro‐inflammatory cytokines such as IFN‐α and tumor necrosis factor (TNF)‐α that induce the regulation of other immune cell types (cDCs, NKs, T cells). cDC1s, corresponding to mouse CD8α^+^ DCs,^17^ represent only 0.03–0.08% of peripheral blood mononuclear cells (PBMCs)^18^ making their study very challenging. In response to TLR3 triggering,^19^ cDC1s are the main producers of type III IFN (IFNλ1/IL29 and IFNλ2/IL28A) leading to stimulation of innate immune cells.^20^ They express XCR1, TLR3 and CLEC9A and exhibit a high cross‐presentation potential of exogenous antigens through MHC class I^21^ hence inducing efficient CD8^+^ cytotoxic T‐cell responses against infected or tumor cells.

DCs are endowed with a high functional plasticity allowing them to orientate immune responses towards diverse profiles depending on the microenvironment. Tumors exploit such versatility of DCs through suppressive pathways to subvert DC functions and escape immunity.^22^, ^23^, ^24^, ^25^ Despite evidence of tumor infiltration by the three DC subsets,^26^, ^27^, ^28^, ^29^, ^30^ their precise pathophysiologic role remains unclear. cDC2s contribute indirectly to antitumor CD8^+^ T‐cell responses by providing help through CD4^+^ T‐cell activation.^31^ In humans, cDC2s were found diminished in many cancer patients’ blood, whereas abundance of cDC2s in primary tumors was correlated with high levels of protective CD4^+^ T cells and better response to ICB.^32^ While being well characterised in human tumors, the role of pDCs in tumor immunity remains enigmatic.^33^ pDC infiltration has been linked with tolerance induction and worse clinical outcome in many tumor types.^34^, ^35^, ^36^, ^37^ In melanoma, tumor‐infiltrating pDCs, recruited from the circulation through the CCR6/CCL20 axis,^38^ are associated with a poor prognosis.^26^, ^39^ pDCs found in metastatic lymph nodes display an impaired IFNα secretion^40^ and express IDO, driving Treg activation and a suppressive microenvironment.^41^ Type I IFN production can be repressed by tumor‐derived soluble factors (PGE2, IL‐10, TGF‐β) and through triggering LAG‐3‐dependent activation resulting in tolerogenic pDC.^39^, ^42^ Hijacking of pDCs by tumor cells induces pro‐tumor regulatory and Th2 immune responses.^26^, ^34^ Yet, pDCs were shown to favor antitumor immunity through their ability to process and cross‐present tumor antigens to T cells^16^, ^43^, ^44^ and subsequently induce adaptive immune responses.^27^, ^44^ Tumor‐infiltrating pDCs can also exhibit a direct cytotoxic potential towards tumor cells in a TNF‐related apoptosis‐inducing ligand (TRAIL)‐dependent manner.^45^, ^46^ Once activated via TLR7/9L, pDCs potentially achieve tumor control through efficient priming of antitumor responses.^47^, ^48^ Tumor antigen‐loaded pDCs properly activated can be vectors for immunotherapy and elicit favorable antitumor immune responses in patients upon vaccination.^49^, ^50^ Besides, the role of cDC1s in antitumor immunity, mostly described in mouse models, is poorly known in humans.^51^, ^52^, ^53^ cDC1s are present in peripheral tissues where they capture tumor antigens and, after migration to tumor‐draining lymph nodes, activate antitumor CD4^+^ and CD8^+^ T cells.^31^ cDC1s revealed to be crucial for the generation and maintenance of antitumor immunity,^54^ T‐cell proliferation and recruitment to the tumor site.^52^ Moreover, cDC1s emerge as important players for the efficacy of targeted therapies and immunotherapies^29^ and are required for effector T‐cell trafficking following adoptive T‐cell therapy.^55^ Interestingly, a recent study highlighted that cDC1s transcriptomic signature in melanoma correlated with a better clinical outcome.^56^ Overall, in melanoma, pDCs still display an enigmatic role, and the physiopathology of cDC1s and cDC2s and interplays between DC subsets are not well characterised. DC subsets require further elucidation to achieve a deeper understanding of melanoma immune subversion.

Thus, DCs play a pivotal yet still puzzling role in the control of tumor development. Their plasticity endorses them with a powerful ability to drive effective antitumor immunity but also with a potential to trigger tolerance and tumor progression. This challenging controversy prompted us to investigate the pathophysiologic role of cDCs and pDCs in the context of melanoma where the precise and coordinated involvement of each DC subset is not fully understood. In this study, we elucidated the phenotypic and functional features of circulating and tumor‐infiltrating BDCA1^+^ cDC2s, BDCA2^+^ pDCs, and BDCA3^+^ cDC1s from melanoma patients and assessed their clinical relevance. Using an innovative multi‐parametric flow cytometry approach that enables to simultaneously depict the three DC subsets, we provide an integrated overview of the features of circulating and tumor‐infiltrating cDC2s, pDCs and cDC1s in melanoma patients together with their clinical impact, uniquely allowing deciphering the interrelations within DC subsets that shaped clinical outcome. Such understanding reveals critical and distinct impact of each DC subset on melanoma progression and brings insights into the mechanism of melanoma escape from immune control. This study opens promising ways to develop new therapeutic strategies to optimise antitumor immunity and achieve better clinical success.

## Results

### Frequencies of circulating and/or tumor‐infiltrating BDCA1^+^ cDC2s, BDCA2^+^ pDCs and BDCA3^+^ cDC1s show perturbations in melanoma patients that drastically correlated with clinical outcome

**Figure 1:**
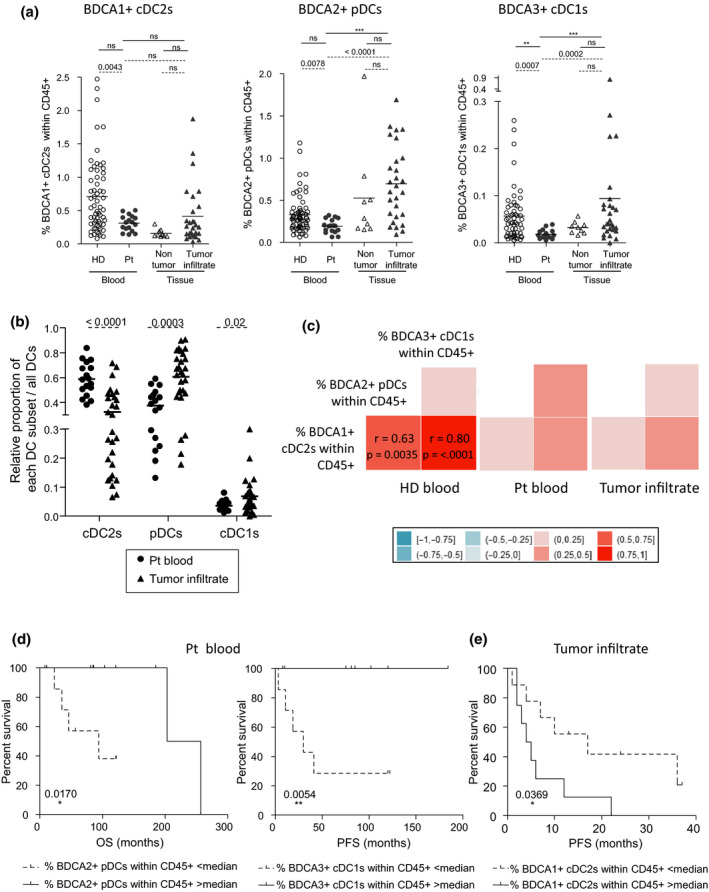
Decreased frequencies of circulating DC subsets in melanoma patients and infiltration level of the tumor site determine the clinical outcome of patients. PBMC and tumor‐infiltrating cells from melanoma patients together with PBMC from HD and control tissues were labelled with specific antibodies allowing depicting the three DC subsets and submitted to flow cytometry analysis. **(a)** Comparative frequencies of BDCA1^+^ cDC2s, BDCA2^+^ pDCs and BDCA3^+^ cDC1s within alive CD45^+^ cells on the blood of healthy donors (HD, open circles, *n* = 56 to 67) and patients (Pt, filled circles, *n* = 17), non‐tumor tissue (tonsils, open triangles, *n* = 9) and tumor infiltrate of melanoma patients (filled triangles, *n* = 23). Results are expressed as percentages of positive cells. Bars indicate mean. *P*‐values were calculated using Mann–Whitney (dashed lines) and Kruskal–Wallis (straight lines) nonparametric tests. * *P* ≤ 0.05, ** *P* ≤ 0.01, *** *P* ≤ 0.001. **(b)** Relative proportions of each DC subsets within all DCs in patients’ blood (*n* = 17) and tumor infiltrates (*n* = 23). Bars indicate mean. *P*‐values were calculated using the Mann–Whitney test. **(c)** Correlation matrix between the three DC subsets frequencies in HD blood (left panel), patient blood (middle panel) and tumor infiltrate (right panel). Spearman correlations r factors with their significant *P*‐values (< 0.05) after Bonferroni–Holm’s correction are noted within the squares. **(d)** Comparative OS (from diagnostic time – left panel) and PFS (from sampling time – right panel) of patients with low or high circulating pDCs or cDC1s, respectively. Groups were separated according to the median percentage of circulating pDCs (0.196%, *n* = 8 or 9 patients/group) or cDC1s (0.016%, *n* = 7–10 patients/group). **(e)** Comparative PFS (from sampling time) of patients with low or high tumor‐infiltrating cDC2s. Groups were separated according to the median percentage of infiltrating cDC2s (0.244%, *n* = 12 patients/group). **(d, e)** Comparison using log‐rank test.

To assess the frequency of cDC2s, pDCs and cDC1s in patient blood and determine whether these DC subsets infiltrate the tumor, we designed a novel multi‐parametric flow cytometry approach that allowed their simultaneous analysis. Among CD45^+^ cells, cDC2s, pDCs and cDC1s were defined as Lin‐HLADR^+^CD11c^+^BDCA1^+^ cells, Lin‐HLADR^+^CD11c^–^BDCA2+ cells and Lin‐HLADR^+^CD11c^+^BDCA3^high^ cells, respectively (Supplementary figure 1a). All analyses were performed comparing blood of patients with healthy donors (HD), and tumor metastasis with non‐tumor tissue (tonsils) (Supplementary figure 1b). The clinical features of patients are reported in Supplementary tables 1 and 2. We first observed a reduced frequency of the three DC subsets in the blood of melanoma patients when compared to HD blood (Figure 1a), whatever disease stage (Supplementary figure 2a). Of note, the three DC subsets infiltrated the tumor of patients whether they were lymph node or cutaneous metastatic tumors (Figure 1a, Supplementary figure 2b). By analysing the relative proportions of the three DC subsets in each group, we further highlighted a massive infiltration by pDCs and cDC1s that shifted the relative proportions of DC subsets at the tumor site when compared to the blood (Supplementary figure 2c, d). By further assessing the relative proportion of each subset compared to all DCs, we underlined that cDC2s decreased while pDCs and cDC1s were enriched in tumors compared to patients’ blood (Figure 1b). Interestingly, modulations for cDC2s and pDCs were witnessed for both lymph node and cutaneous metastases, whereas the increased frequency of cDC1s was detected only for lymph node metastases (Supplementary figure 2e). To assess the interrelations between DC subsets (‘inter‐DCs’), we performed correlation analysis using the data collected on frequencies of DCs for HD blood, patient blood, and tumor infiltrate. We found that cDC2s displayed strong positive interrelations with pDCs and cDC1s in HD blood, with both *P*‐values lower than 0.005, and that such correlation was lost in both blood and tumor of melanoma patients (Figure 1c). Notably, the observed alterations of frequencies of DCs had an impact on clinical outcome of patients (Supplementary table 3a). Indeed, high frequencies of circulating pDCs and cDC1s positively impacted progression‐free survival (PFS) and overall survival (OS) (Figure 1d, Supplementary figure 2f), whereas high frequencies of tumor‐infiltrating cDC2s have a negative impact on PFS of melanoma patients (Figure 1e, Supplementary table 3a). Thus, we unveiled for the first time melanoma infiltration by cDC1s. Our data also showed that frequencies of circulating and tumor‐infiltrating DCs have a distinct impact on clinical outcome in melanoma patients depending on the DC subset.

### The basal activation status of BDCA1^+^ cDC2s, BDCA2^+^ pDCs and BDCA3^+^ cDC1s, observed in melanoma patients, impacts clinical outcome

**Figure 2:**
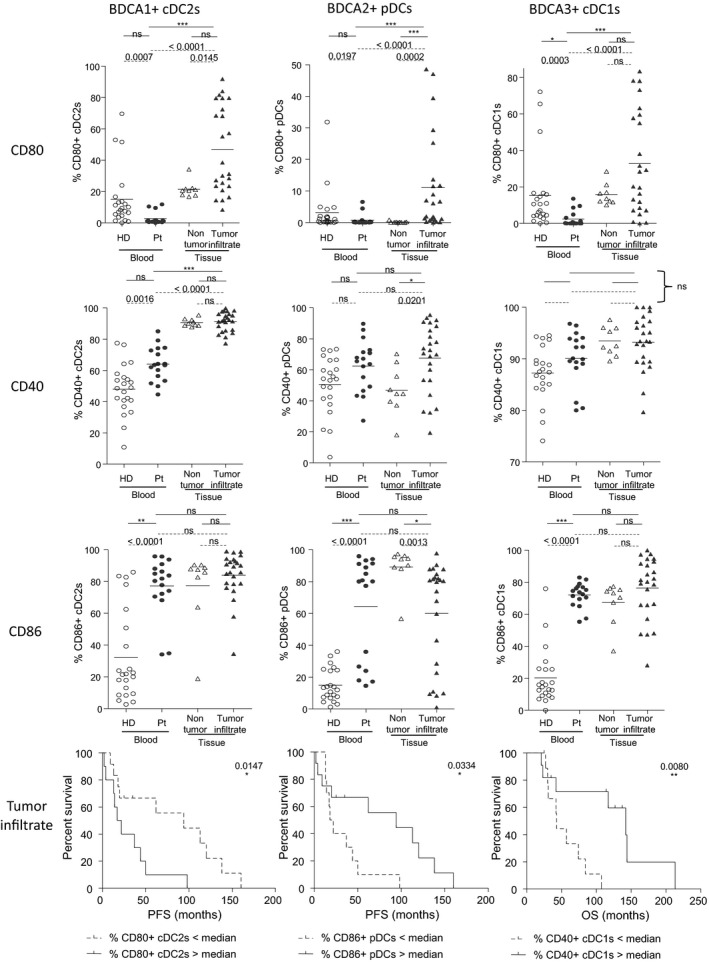
Peripheral and tumor‐infiltrating DC subsets from melanoma patients displayed an overall activated basal status. The expression of the co‐activation molecules CD80, CD40 and CD86 on DC subsets was analysed by flow cytometry on PBMCs and tumor‐infiltrating cells of melanoma patients, HD or non‐tumor tissue controls. **(a)** Expression levels of the co‐stimulatory molecules CD80, CD40 and CD86 on the three DC subsets from the blood of healthy donors (HD, open circles, *n* = 22) and melanoma patients (Pt, filled circles, *n* = 17), tumor infiltrates of melanoma patients (filled triangles, *n* = 23) and non‐tumor tissues (tonsils, open triangles, *n* = 9). Results are expressed as percentages of positive cells within the corresponding DC subset. Bars indicate mean. *P*‐values were calculated using Mann–Whitney (dashed lines) and Kruskal–Wallis with post hoc Dunn’s multiple comparison (stars) nonparametric tests. *P ≤ 0.05, **P ≤ 0.01, ***P ≤ 0.001. **(b)** Comparative PFS (from diagnosis time) of patients with low or high tumor‐infiltrating CD80^+^ cDC2s (left panel), CD86^+^ pDCs (middle panel), and comparative OS (from diagnosis time) of patients with low or high tumor‐infiltrating CD40^+^ cDC1s (right panel). Groups were separated using the median percentage of tumor‐infiltrating CD80^+^ cDC2s (34.96%), CD86^+^ pDCs (80.15%) and CD40^+^ cDC1s (93.42%) (*n* = 10–12 patients/group) among each DC subset, respectively. Comparison using log‐rank test.

To further characterise the features of circulating and tumor‐infiltrating DC subsets in the context of melanoma, we investigated their basal activation status (Supplementary figure 3a) and assessed their clinical relevance (Supplementary table 3b). Circulating cDC2s, pDCs and cDC1s displayed a lower expression of CD80 but a higher expression of CD40 and CD86 compared to control groups (percentages and/or MFI) (Figure 2a, Supplementary figure 3b), which was similar at early and late stages of the disease (Supplementary figure 3c). In addition, compared to non‐tumor tissue infiltrating DCs, all tumor‐infiltrating DC subsets exhibited an increased CD80 expression together with an upregulation of CD40 for pDCs and cDC1s, while the level of CD86 was found to be downregulated on tumor‐infiltrating pDCs (Figure 2a, Supplementary figure 3b). No differences were observed between lymph node and cutaneous metastases (Supplementary figure 3d). Interestingly, higher proportions of tumor‐infiltrating CD40^+^ or CD86^+^ pDCs were linked to longer PFS, whereas higher proportions of tumor‐infiltrating CD80+ cDC2s or pDCs foresaw worse clinical outcome as it was linked with shorter PFS (Figure 2b, Supplementary figure 3e). Strikingly, we also found that higher proportions of circulating or tumor‐infiltrating CD40^+^ cDC1s and tumor‐infiltrating CD40^+^ pDCs predicted better clinical outcome (Figure 2b, Supplementary figure 3e, f). Altogether, these results indicate that the perturbed activation status of DC subsets in the blood and tumor of melanoma patients differentially impacts clinical outcome.

### DC basal activation status allows clustering of patients and highlights perturbed interrelations between the three DC subsets in the context of melanoma

**Figure 3:**
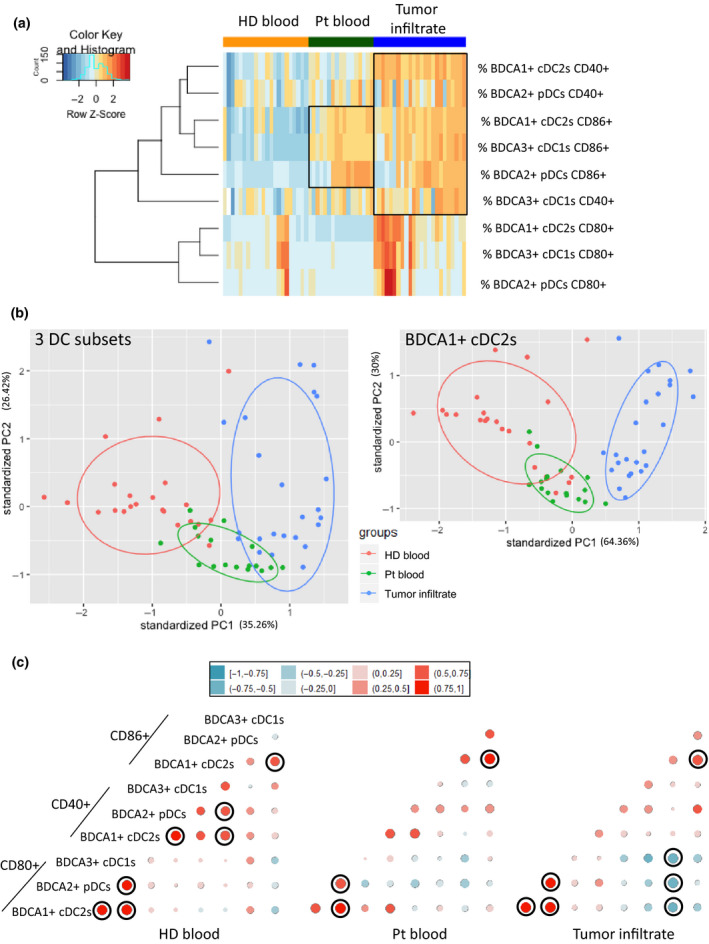
The frequency and activation status of circulating and tumor‐infiltrating DC subsets in melanoma patients allowed their distinct clustering from healthy donors. **(a)** Heat map based on the expression of co‐stimulatory molecule (CD80, CD40, CD86) by the three DC subsets in each sample type (HD blood, patient blood and tumor infiltrate). **(b)** Principal component analysis (PCA) based on DC subsets co‐stimulatory molecules expression for the three DC subsets (left panel) or only cDC2s (right panel – including graph of variables). **(c)** Correlation matrix between the three DC subsets expressing co‐stimulatory molecules in HD blood (left panel), patient blood (middle panel) and tumor infiltrate (right panel). Spearman correlations with significant *P*‐values (< 0.05) after Bonferroni–Holm’s correction are circled in black.

To understand whether the activation profile of each DC subset could distinguish patients from HD, we performed Euclidean distance‐based hierarchical clustering and ran PCA analyses. Heat map based on co‐stimulatory molecules expression illustrated the distinct patterns of DC features in blood and tumor infiltrate of melanoma patients compared to HD as previously mentioned (Figure 3a). Furthermore, each group was located in distinct areas of PCA analyses (based on PC1 and PC2), thus allowing intra‐group clustering by activation profile of DC subsets (Figure 3b, left panel and Supplementary figure 3g left panel). Notably, by further looking at each subset, such observation was mostly due to features of cDC2s (Figure 3b right panel, Supplementary figure 3g right panel, h). In addition, to assess interrelations between DC subsets, we performed statistical correlation analyses between DC activation markers for each group (Figure 3c, Supplementary figure 3i). Graphical spearman correlation matrix revealed strong positive correlations between DC features in HD (Figure 3c, left panel) which were altered in blood and tumor infiltrate of patients (Figure 3c, middle and right panels). Positive correlations between CD40‐expressing DC subsets were lost in patients, and negative correlations between CD80‐expressing DCs and CD86^+^ pDCs emerged in tumor infiltrates, which fits with impacts observed on clinical outcomes (Figure 2b, Supplementary figure 3f). Thus, we uncovered that the basal activation profile of DCs allowed clustering of patients and indicated that melanoma may drive coordinated regulations between DC subsets.

### Circulating and/or tumor‐infiltrating cDC2s and pDCs displayed an altered capacity to upregulate activation markers upon TLR triggering in link with clinical outcomes

**Figure 4:**
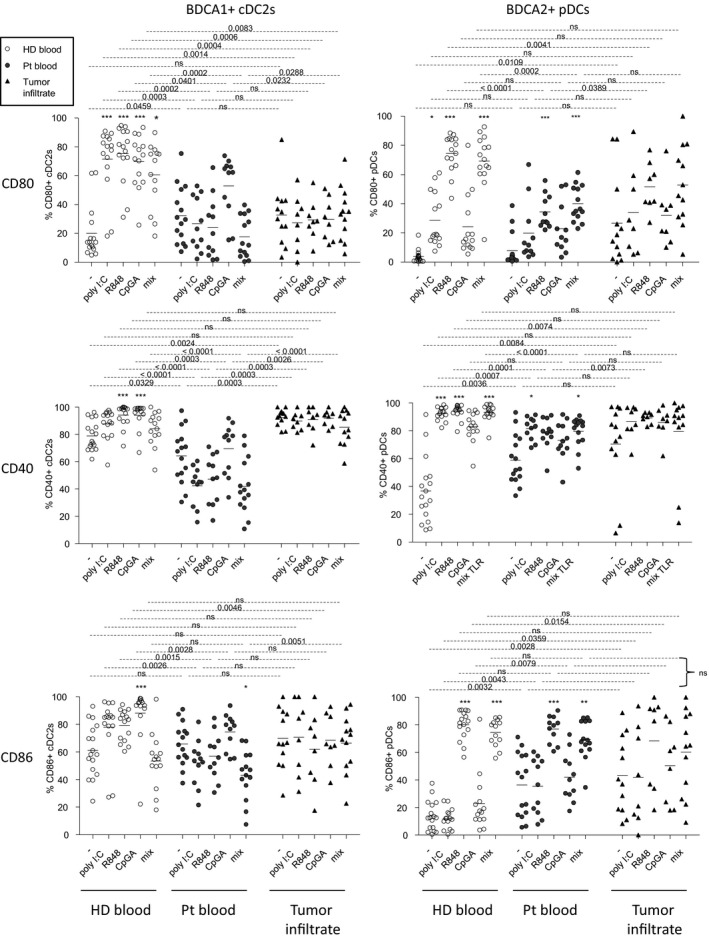
Circulating and tumor‐infiltrating BDCA1^+^ cDC2s and BDCA2^+^ pDCs from melanoma patients displayed defective maturation after TLR stimulation. Cell suspensions from blood (HD, *n* = 17; Pt, *n* = 15) or tumor infiltrates (Pt, *n* = 14) were stimulated or not for 21h with or without TLR ligands (polyI:C, R848 or CpG_A_) alone or mixed together (mix), and the expression of the co‐stimulatory molecules CD80, CD40 and CD86 was measured on BDCA1^+^ cDC2s and BDCA2^+^ pDCs using flow cytometry. Results are expressed as percentages of positive cells within the corresponding subset. Bars indicate mean. Stars indicate significant differences compared to the control condition without stimulation (−) from each group. *P‐*values were calculated using Mann–Whitney tests (dashed lines) and Kruskal–Wallis with post hoc Dunn’s multiple comparison (stars) nonparametric tests. * *P* ≤ 0.05, *** *P* ≤ 0.001.

The functional capacity of circulating and tumor‐infiltrating DCs to respond to TLR triggering was subsequently investigated by monitoring the expression of activation molecules by cDC2s and pDCs in response to specific single or combined TLR ligands after 20h of culture, offering also the opportunity to assess cross‐regulation between DC subsets (Figure 4 and Supplementary figure 4a). Due to cDC1s scarcity after 20 h of culture, their analysis did not reach quality criteria and this DC subset could not be considered, yet their potential cross‐talk with the other DC subsets still being effective during culture. In absence of TLR stimulation, circulating and tumor‐infiltrating 20 h‐cultured cDC2s and pDCs displayed higher levels of CD80, CD40 and/or CD86 (% and/or MFI) when compared to HD (Figure 4 and Supplementary figure 4a). Interestingly, higher expression of CD40 and CD80 on cultured tumor‐infiltrating cDC2s and pDCs, respectively, was associated with a worse clinical outcome as they were linked with shorter PFS (Supplementary figure 4b). Strikingly, upon TLR triggering, response of circulating and tumor‐infiltrating cDC2s was totally abrogated for all markers, except for CD40 on tumor‐infiltrating cDC2s, who already exhibited a high level in unstimulated conditions and couldn't be interpreted (Figure 4 and Supplementary figure 4a). Moreover, circulating pDCs exhibited an altered response when compared to HD (% and/or MFI), while tumor‐infiltrating pDCs still upregulated CD80 and CD40 (MFI) upon R848 stimulation (Figure 4 and Supplementary figure 4a). Levels of CD86 on cDC2s and pDCs tended to be lower in late stage compared to early stage patients (Supplementary figure 4c). No differences were observed between lymph node and cutaneous metastases (Supplementary figure 4d). To assess the clinical relevance of our findings, we performed correlations between cultured circulating or tumor‐infiltrating DC features (activation status upon TLRL stimulation) and clinical outcomes (Supplementary tables 4–6). Upon R848 stimulation, high proportions of circulating CD80 or CD40‐expressing cDC2s were linked with a good clinical outcome (both for PFS and OS from diagnosis time), whereas high proportions of circulating CD86‐expressing pDCs after CpG_A_ stimulation were associated with a bad clinical outcome (Supplementary figures 4e, 5a). Furthermore, higher levels of CD86 on tumor‐infiltrating cDC2s upon TLR stimulation (R848 or mix) were linked with a worse clinical outcome both for PFS and OS from sampling time (Supplementary figures 4f, 5b). Moreover, high expressions of CD80 or CD86 by tumor‐infiltrating pDCs after TLR stimulation were linked with a worse clinical outcome (Supplementary figure 5c). To have a global view of the specific features of DC subsets in patients compared to controls, we executed Euclidean distance‐based hierarchical clustering and ran PCA analyses. Heat map based on co‐stimulatory molecules upregulation after TLR triggering highlighted distinct patterns of DC features between each group, CD80 expression being a major driving component (Supplementary figure 6a). In addition, each group was located in distinct areas of PCA analyses (based on PC1 and PC2), thus allowing intra‐group clustering when considering the activation profile of DC subsets (Supplementary figure 6b). By further looking at each subset, such observation was mostly due to features of cDC2s (Supplementary figure 6c). To further assess interrelations between DC subsets based on activation marker expression after TLR triggering, we performed Spearman correlations between immune parameters. A correlation matrix revealed modifications of intra‐DCs’ and inter‐DCs’ features in melanoma patients compared to HD (Supplementary figure 6d, e). Positive intra‐DCs’ correlations seen in HD were lost in patients, and positive and negative intra‐DCs’ and inter‐DCs’ correlations between cDC2s and pDCs emerged in patient blood and tumor infiltrates. Interestingly, upon TLR triggering, CD40‐ and CD80‐expressing cDC2s were positively correlated in patient blood, and both impacted clinical outcomes the same way (Supplementary figures 5a, 6e). We further observed that within tumor infiltrates, there are positive correlations between highly activated pDCs upon 20h culture and upregulation of co‐stimulatory molecules upon TLR triggering, both linked with a bad clinical outcome (Supplementary figures 4b, 5c, 6e). Regarding inter‐DCs’ analyses, CD86 expression on tumor‐infiltrating pDCs and cDC2s upon TLR triggering was linked together, similarly impacting clinical evolution (Supplementary figures 5b, c, 6e). On the contrary, CD80 and CD86 markers were negatively correlated between tumor‐infiltrating cDC2s and pDCs upon TLR stimulation, reflecting complex cross‐talks within DC subsets (Supplementary figure 5c). Taken together, these results highlight that circulating and/or tumor‐infiltrating cDC2s and pDCs display an altered capacity to upregulate activation markers upon TLR triggering and that melanoma drastically modulates interrelations between DC subsets.

### Intrinsic cytokine production and ability of dendritic cells to respond to TLR triggering dictate the clinical outcome of melanoma patients

**Figure 5:**
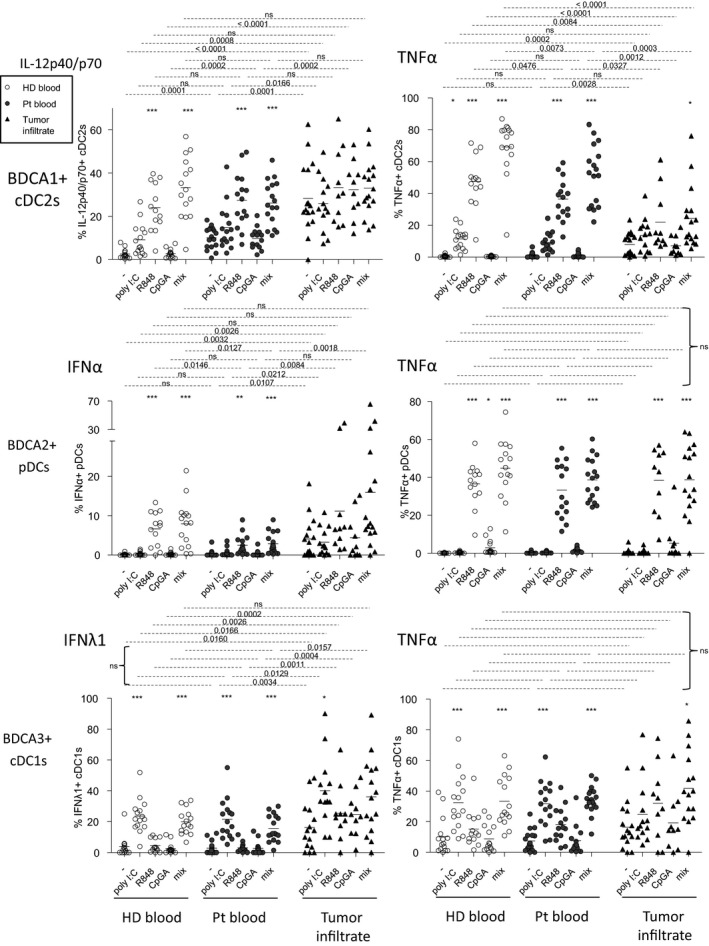
Upon TLR triggering, TNFα and IFNα productions by BDCA1^+^ cDC2s and BDCA2^+^ pDCs, respectively, from blood and tumor were impaired, whereas IFNλ1 and TNFα productions by circulating and tumor‐infiltrating BDCA3^+^ cDC1s remained fully functional in the context of melanoma. Cell suspensions from blood (HD, *n* = 15, open circles; Pt, *n* = 17, filled circles) or tumor infiltrates (Pt, *n* = 16, filled triangles) were stimulated for 5h with or without TLR‐L (polyI:C, R848 or CpG_A_) alone or mixed together, and the production of cytokines was evaluated by intracellular cytokine staining using flow cytometry. Results are expressed as percentages of cytokine‐expressing cells within the corresponding DC subset. Bars indicate mean. Stars indicate significant difference with control without stimulation from each group. *P*‐values were calculated using Mann–Whitney (dashed lines) and Kruskal–Wallis with post hoc Dunn’s multiple comparison (stars) nonparametric tests. *P ≤ 0.05, **P ≤ 0.01, ***P ≤ 0.001.

**Figure 6:**
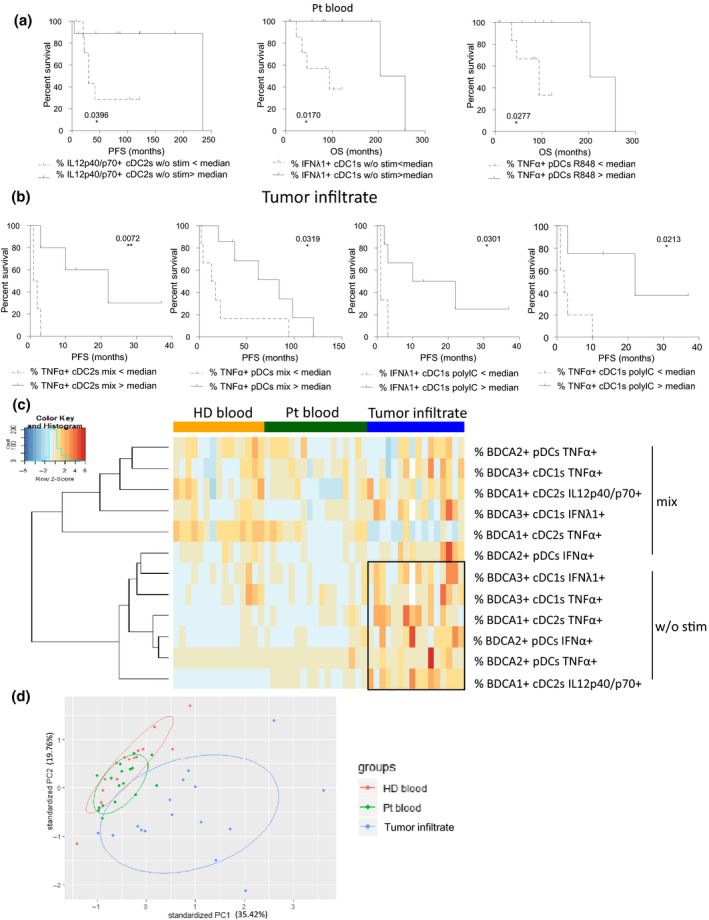
High productions of IL12p40/p70, TNFα and IFNλ1 by circulating and tumor‐infiltrating cDC2s, pDCs and cDC1s after TLR stimulation positively impacted melanoma patients’ clinical evolution. Cell suspensions from blood (HD, *n* = 15; Pt, *n* = 17) or tumor infiltrates (Pt, *n* = 16) were stimulated for 5h with or without TLR‐L (polyI:C, R848 or CpG_A_) alone or mixed together, and the production of cytokines was evaluated by intracellular cytokine staining using flow cytometry. The proportions of cytokine‐expressing cells were correlated with the clinical parameters of the corresponding patients. **(a)** Comparative PFS (from diagnosis time – left panel) and OS (from diagnosis time – middle and right panels) of patients with low or high circulating IL12p40/p70^+^ cDC2s and IFNλ1 + cDC1s in absence of *ex vivo* stimulation, and TNFα^+^ pDCs after R848 stimulation. Groups were separated using the median percentage of the corresponding parameters (IL12p40/p70^+^ cDC2s: 10.58%, TNFα+ pDCs: 29.51%, and IFNλ1^+^ cDC1s :1.22% (*n* = 7 or 9 patients/group). **(b)** Comparative PFS (from diagnosis or sampling time) of patients with low or high TNFα^+^ cDC2s or pDCs and IFNλ1^+^ or TNFα^+^ cDC1s after stimulation of tumor‐infiltrating cells with mix TLR‐L or polyI:C, respectively. Groups were separated using the median percentage of tumor‐infiltrating TNFα^+^ cDC2s (22.80%) or pDCs (45.43%) after TLR‐L mix stimulation, and IFNλ1^+^ (36.23%) or TNFα^+^ (18.62%) cDC1s after polyI:C stimulation (*n* = 3 or 7 patients/group). **(a, b)** Comparison using log‐rank test. **(c)** Heat map based on intracellular cytokine expressions (IL‐12p40/p70, IFNα, IFNλ1, TNFα) by the three DC subsets following stimulation or not with the mixture of TLR‐L (polyI:C, R848 and CpG_A_) in each sample type (HD blood, patient blood and tumor infiltrate). **(d)** PCA based on of intracellular cytokine expressions (IL‐12p40/p70, IFNα, IFNλ1, TNFα) by the three DC subsets after 5h of culture with or without TLR triggering (polyI:C, R848 or CpG_A_ alone or mixed together) in HD blood, patient blood and tumor infiltrates.

Cytokines are critical for DCs to cross‐talk between them and with antitumor effectors, hence shaping antitumor immunity. Thus, we further investigated the functional capacity of circulating and tumor‐infiltrating DCs to produce cytokines upon TLR triggering by performing intracellular labelling of IL‐12p40/p70, TNFα, IFNα and IFNλ1 within DC subsets stimulated or not with single or combined TLR‐Ls (Figure 5, Supplementary figure 7a). In absence of *ex vivo* stimulation by TLR‐L (condition ‘stim –’), we observed a higher production of IL‐12p40/p70 by circulating and tumor‐infiltrating cDC2s and of IFNα and IFNλ1 by tumor‐infiltrating pDCs and cDC1s, respectively, compared to HD. Notably, such features were linked (in the case of cDCs) to a better clinical outcome (Figure 5, Figure 6a, Supplementary tables 7 and 8), revealing that *in situ* activation of DCs could occur in patients and favor tumor immune control. Following *ex vivo* TLR triggering, we observed that in most conditions, all circulating DC subsets were able to secrete cytokines (Figure 5). Even though productions of TNFα and IFNα were slightly impaired, respectively, in circulating cDC2s and pDCs upon R848 and mix TLR stimulation, production of IL‐12p40/p70 by circulating cDC2s, TNFα or/and IFNλ1 by circulating pDCs and cDC1s remained similar to HD. We noticed that the functionality of circulating DC subsets tended to be more altered at late stage compared to early stage disease, especially for cDC1s (Supplementary figure 7b). Furthermore, high capacity to produce TNFα by circulating pDCs upon R848 stimulation was associated with improved clinical outcome since it had a positive impact on OS (Figure 6a, Supplementary figure 8a). Strikingly, while tumor‐infiltrating cDC2s and pDCs were defective in response to TLR‐L stimulation, TNFα and IFNλ1 production by tumor‐infiltrating cDC1s remained similar to HD (Figure 5). In addition, such functional defects of cDC2s and pDCs were noticed for both lymph node and cutaneous metastases, while the IFNλ1 production by tumor‐infiltrating cDC1s was higher in cutaneous compared to lymph node metastases (Supplementary figure 8b). Outstandingly, high proportions of tumor‐infiltrating TNFα‐producing DCs, IFNα^+^ pDCs and IFNλ1^+^ cDC1s after TLR triggering were linked to a better clinical outcome (Figure 6b, Supplementary figure 8a). To further analyse the differences between DC subsets, we executed Euclidean distance‐based hierarchical clustering and ran PCA analyses. Heat map based on cytokine production after TLR triggering emphasised distinct patterns of DC features and showed differences of cytokine production without TLR triggering in the tumor infiltrate when compared to HD (Figure 6c). In addition, samples from tumor infiltrates were located in a distinct area of the PCA analyses (based on PC1 and PC2), thus allowing intra‐group clustering (between patients and HD) when considering cytokine production by DC subsets (Figure 6d, Supplementary figure 8c). By further looking at each subset, such observations were mostly due to features of cDC2s (Supplementary figure 8d). To further assess interrelations between DC subsets based on their cytokine production after TLR triggering, we performed Spearman correlations and underlined modifications of intra‐DCs’ and inter‐DCs’ features in melanoma patients compared to HD (Supplementary figure 8e, f). First of all, for the three circulating DC subsets of HD, production of each cytokine was positively correlated in response to the different TLR‐L stimulation for most of them, whereas production of different cytokines by a given DC subset was not interrelated (Supplementary figure 8f). Such interrelations were modulated in blood and tumor of patients, as new relations emerged within cytokine‐producing DCs between different TLR triggering. Interestingly, production of IL12p40/p70 and TNFα by circulating cDC2s, and of IFNα and TNFα by tumor‐infiltrating pDCs were correlated together in patients (Supplementary figure 8f). Regarding inter‐DCs’ relationship, positive correlations between TNFα‐producing cDC2s and pDCs observed in HD blood were lost in melanoma patients (Supplementary figure 8f). Notably, upon TLR triggering, positive correlations between IFNλ1‐producing cDC1s and IFNα/TNFα‐producing pDCs appeared within tumor microenvironment and were both linked likewise with clinical outcome (Supplementary figure 8a, f and Figure 6b). Thus, these findings highlight that while cDC2s and pDCs’ functionality was not optimal in melanoma patients, circulating and tumor‐infiltrating cDC1s preserved a potent functionality upon TLR triggering, improving clinical outcome, and endorsing the importance of targeting cDC1s *in vivo* to enhance antitumor immunity.

### Cytokine secretions in response to TLR triggering are associated with a good clinical outcome and reveal modulation of the cross‐talks between DC subsets in the context of melanoma

**Figure 7:**
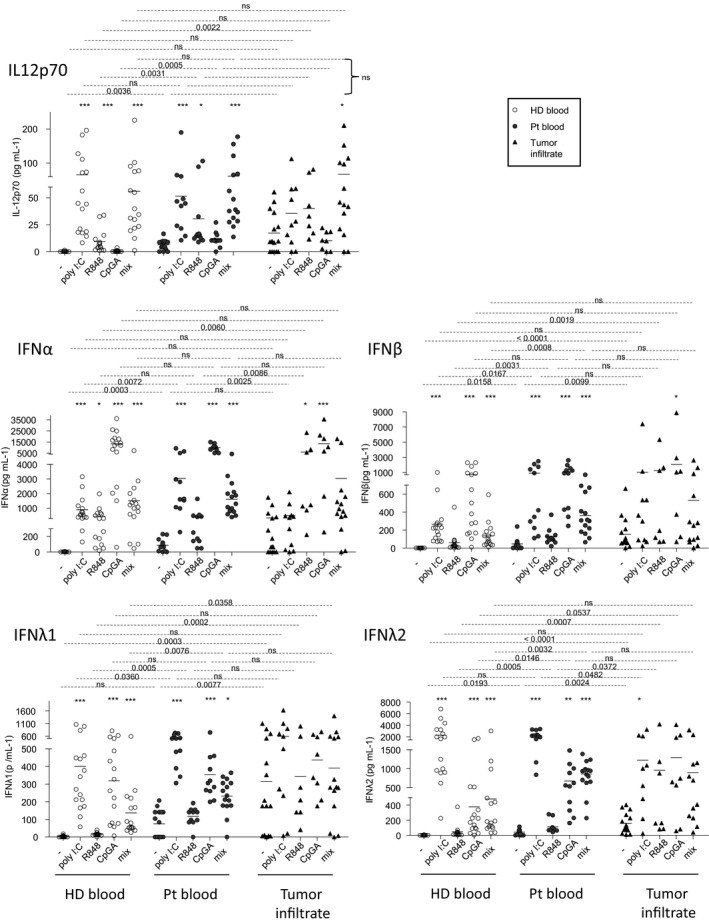
Enhanced secretions of IL12p70, type I and III IFNs both with and without *ex vivo* TLR triggering arised from circulating and tumor‐infiltrating cells of melanoma patients. Cell suspensions from blood (HD, *n* = 18, open circles; Pt, *n* = 15, filled circles) or tumor infiltrates (Pt, *n* = 15, filled triangles) were stimulated for 20 h with or without TLR ligands (polyI:C, R848 or CpG_A_) alone or mixed together, and the culture supernatants were examined for the presence of IL‐12p70, IFNα, IFNβ, IFNλ1 and IFNλ2 by Luminex technology. Results are expressed in pg mL^–1^. Bars indicate mean. Stars indicate significant differences of the stimulated conditions compared to unstimulated ones within each group. *P*‐values were calculated using Mann–Whitney (dashed lines) and Kruskal–Wallis with post hoc Dunn’s multiple comparison (stars) nonparametric tests. *P ≤ 0.05, **P ≤ 0.01, ***P ≤ 0.001.

To obtain a larger view on cytokine production and different global quantitative measurements, we investigated the impact of melanoma on cytokine secretion by circulating or tumor‐infiltrating immune cells upon TLR triggering. We assessed IL‐12p70, IFNα, IFNβ, IFNλ1 and IFNλ2 secretions using Luminex Technology in culture supernatants from patients after 20 h of culture with or without TLRL. In absence of *ex vivo* stimulation by TLRL, we observed significant increased secretions of IL12p70, IFNα, IFNβ and IFNλ2 in patient blood, and increased productions of IFNβ, IFNλ1 and IFNλ2 in tumors when compared to the control group (Figure 7), further demonstrating the basal activation of DCs within tumor microenvironment. Cytokine productions were similar at early and late stages (Supplementary figure 9a), and between lymph node and cutaneous metastases (Supplementary figure 9b). By studying correlations between immune features and clinical data, we revealed that such an increase of IFNα secretion in patient blood was linked with better clinical outcome, whereas in tumors it had a negative impact on OS (from diagnosis time) (Supplementary figure 9c, d, Supplementary tables 9, 10). Following *ex vivo* TLR triggering of circulating immune cells of patients, secretions of IFNα, IFNβ, IFNλ1 and IFNλ2 were observed upon stimulation with polyI:C, CpG_A_, and the mixture of TLRL, whereas IL12p70 was also secreted after stimulation with polyI:C, R848 or the mix. Such TLRL‐dependent secretions were significantly increased for IL‐12p70 (R848), IFNα (polyI:C), IFNβ (polyI:C, mix), IFNλ1 (mix), and IFNλ2 (polyI:C, CpG_A_, mix) in circulating immune cells of patients when compared to controls (Figure 7), even though proportions of DC subsets were decreased in patients (Figure 1). Regarding tumor‐infiltrating cells, R848 and/or CpG_A_ stimulation induced, respectively, IFNα and IFNβ secretions at comparable levels between patients and HD blood, whereas polyI:C and mix, respectively, induced increased IFNα and IL‐12p70 secretions in patients when compared to controls. Strikingly, high levels of IL12p70, IFNα, and IFNβ secretion in patient blood (after R848 or mix) and tumor (after R848) were linked with better clinical outcome (Supplementary figure 9c, d). Regarding type III interferon secretion in tumors, TLR triggering could not further improve the high basal level of IFNλ1 secretion, and the polyI:C‐dependent secretion of IFNλ2 in tumor‐infiltrating cells was similar to controls (Figure 7). To further analyse the differences in cytokine secretion between groups, we executed Euclidean distance‐based hierarchical clustering and ran PCA analyses. Heat map emphasised differences of cytokine secretion without TLR triggering in patient blood and tumor infiltrate when compared to HD (Supplementary figure 9e), with higher level of cytokines secreted by tumor‐infiltrating DC. PCA analyses (based on PC1 and PC2) did not provide further information on the differences between HD and patients (Supplementary figure 10a). To further assess interrelations between cytokines secreted after TLR triggering, we performed Spearman correlations (Supplementary figure 10b, c). In HD, we observed that secretions of type I IFN (IFNα and IFNβ) upon different TLRL stimulation (respectively for R848 or mix/CpG_A_) were positively correlated, which was not the case for the other cytokines. Furthermore, upon a single stimulation, different cytokines were regulated together, as highlighted for IFNλ1 and IFNλ2 upon CpG_A_ or mix, and IFNα and IFNλ2 upon R848. Interestingly, such observations were not seen in blood of patients, yet new inter‐regulations emerged between type I and III IFN secretions upon CpG_A_ or mix stimulation, revealing that the interrelations between circulating DC subsets were disturbed in the context of melanoma. Notably, within tumor microenvironment, the increased levels of IL12p70, IFNβ, IFNλ1 and IFNλ2 observed in absence of *ex vivo* stimulation were correlated together (Supplementary figure 10c). Some interactions between type I and III IFNs seen in HD were preserved in tumors of patients, whereas new ones occurred, such as between IFNλ1/IFNλ2 and IFNβ (mix) or IFNα/IFNβ and IFNλ2 (polyI:C), underlying that cDC1s, whose functionality is poorly affected by the tumor, might positively impact pDCs.

## Discussion

**Figure 8:**
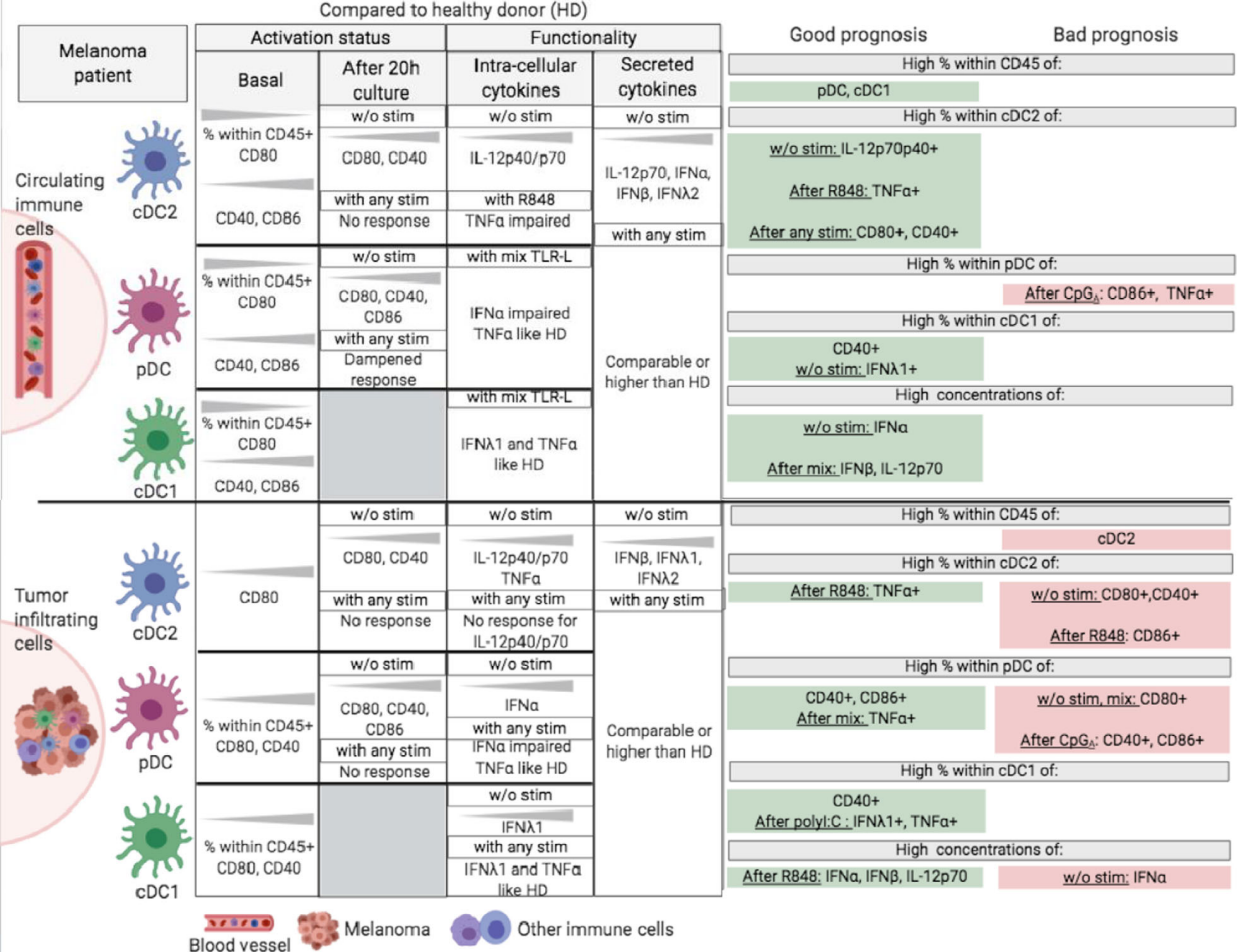
Graphical summary of the main features of circulating and tumor‐infiltrating DC subsets in melanoma patients and their impact on clinical outcome. The left part of the figure displays phenotypic and functional perturbations observed in both blood (upper part) and tumor (bottom part) of melanoma patients when compared to healthy donors. The right part of the figure highlights DC‐based prognosis factors of clinical evolution. This figure has been created with BioRender science illustration tool.

Dendritic cells display a critical role in orchestrating and shaping immune responses. Despite evidence of tumor infiltration by DC subsets, their pathophysiologic role as well as their coordinated involvement in the control of tumor development remain enigmatic. In this study, we provide an integrated overview of the phenotypic and functional features of circulating and tumor‐infiltrating cDC2s, pDCs and cDC1s in melanoma patients together with their interrelations and their clinical impact (summarised in Figure 8 and Supplementary figures 11 and 12). Such understanding reveals critical and distinct impacts of each DC subset on melanoma progression. This study opens exciting ways to develop new therapeutic strategies to optimise antitumor immunity and achieve better clinical success.

Our work, even though performed in a rather small cohort of patients, represents the first study depicting simultaneously the phenotypic and functional features of the three main DC subsets both in circulation and within tumor microenvironment in the context of melanoma, and to assess their impact on clinical outcome. For the first time, we revealed the coexistence of distinct DC subsets in melanoma tumors using a highly specific multi‐parametric flow cytometry approach. We observed a reduction of the frequency of the three DC subsets in the blood of melanoma patients compared to HD together with an infiltration of the tumor, which was massive for pDCs and cDC1s, revealing an active recruitment of the three DC subsets to the tumor. Furthermore, we found that perturbations of the frequencies of circulating and tumor‐infiltrating DC subsets in melanoma patients drastically correlated with clinical outcome, with distinct impacts depending on the DC subset. Importantly, all the phenotypic and functional modulations of DC subsets were not linked with disease stage (not shown). High frequencies of circulating pDCs and cDC1s positively impact time to relapse and overall survival, as recently confirmed by the exploitation of big TCGA cancer data sets,^53^ whereas high frequencies of tumor‐infiltrating cDC2s have a negative impact on PFS of melanoma patients. Our multi‐parametric flow cytometry approach is complementary of other studies mostly investigating DC subsets in cancer patients based on *in situ* characterisation of DCs by immunohistochemistry or high dimensional technologies such as CyTOF and transcriptomic signatures of DC subsets.^23^, ^24^, ^28^ The levels of circulating cDCs were shown to correlate with melanoma activity as their persistent defect reflected high risk of tumor recurrence^24^ and the abundance of cDC2s relative to Treg predicted survival.^32^ A negative prognostic impact of cDC2 infiltration has been also highlighted in lung cancer.^57^ Furthermore, in melanoma, circulating pDC proportions were shown to decrease at advanced stages^58^ and high levels of tumor‐infiltrating pDC were associated with poor prognosis^26^. Such observations were also highlighted in other tumor types such as breast cancer^35^, ^36^ and ovarian tumors.^37^ In line with our data, high expression of cDC1s transcriptomic signature in various tumor microenvironments correlated with a better clinical outcome^30^, ^51^ including melanoma.^53^, ^56^ Importantly, we uncovered that the proportion of cDC1s in the blood was the most accurate parameter to predict clinical outcome of melanoma patients (naïve of treatment by immunotherapies). This novelty could be very useful to optimise disease management and better orientate decisions by clinicians to define the best treatment leading to increased clinical success.

The intra‐group clusterings illustrated on heat maps and PCA analyses based on phenotypic or functional parameters of all DC subsets highlighted distinct patterns of DC features in each group. Such observations underline specific features of cDCs and pDCs in the blood and tumor infiltrate of melanoma patients compared to HD. All DC subsets displayed an overall higher basal activation status in both blood and tumor of melanoma patients compared to controls, which is in line with our previous observations^26^ and reports in other tumor types.^35^, ^37^ Upon *ex vivo* TLR triggering, the ability of circulating and tumor‐infiltrating cDC2s to upregulate co‐stimulatory molecules was totally abrogated, while the capacity of pDCs to respond was maintained. Despite impairments of productions of TNFα and IFNα, respectively, by circulating cDC2s and pDCs, production of IL‐12p40/p70 by circulating cDC2s together with TNFα and/or IFNλ1 respectively by circulating or tumor‐infiltrating cDC1s and pDCs remained similar to HD. Our findings are consistent with previous studies showing for pDCs a modulated activation status and impaired functionality^26^ and a capacity of DC subsets to respond to TLR triggering.^59^ Overall, it clearly emerges from our study that, upon TLR triggering, high proportions of CD40/CD86‐expressing DCs were mainly linked to a better clinical outcome in the circulation but mostly associated with worse prognosis in the tumor, whereas high levels of both circulating and tumor‐infiltrating cytokine‐producing DCs were generally connected to a better clinical outcome. While circulating and/or tumor‐infiltrating cDC2s and pDCs displayed an altered capacity to respond to TLR triggering for at least one of the studied parameter, the functional competences of circulating and tumor‐infiltrating cDC1s remained potent and intact. Thus, despite cDC2s and pDCs defective functions, circulating and tumor‐infiltrating cDC1s preserved a potent functionality associated with improved prognosis, suggesting that cDC1s could escape from tumor‐induced immune subversion (Supplementary figure 12).

Up to now, the common vision is the immune‐subversion of DC subsets in cancer. Many studies have described functional defects of DCs in cancer with a switching towards an immunosuppressive activation state subsequently impairing antitumor immunity.^22^, ^23^, ^24^ Our work unveiled that cDC2s and pDCs exhibit defective functional features, while cDC1s display full competency. Even if cDC1s are numerously inferior to the other DC subsets, their potent capacities represent a driving force to trigger protective antitumor immune responses. Such observation enlightened the importance of targeting cDC1s in immunotherapeutic strategies to enhance antitumor immunity and improve clinical outcomes. Recent evidence suggests that cDC1s are critically involved in the triggering of tumor‐specific T‐cell responses and orchestration of cancer immune control.^51^ cDC1s uniquely combined several key features not simultaneously expressed by other cell types,^52^ allowing the generation and maintenance of an effective antitumor immunity. cDC1s are pivotal to trigger antitumor immunity^60^ but also required for trafficking of T cells to the tumor,^55^ for the maintenance of CTL, and generation of memory preventing relapse.^52^ In light of their outstanding properties, the exploitation of cDC1s for therapeutic developments is promising. Strategies aiming at enhancing the abundance and function of cDC1s in tumors are promising new ways to improve patients’ clinical outcome and response to immunotherapies and could consist in restoring cDC1 numbers, enhancing cDC1 antigenicity and adjuvanticity, or directly targeting them through CLEC9A.

In absence of *ex vivo* stimulation by TLR‐L, we observed a higher production of IL‐12p40/p70 by circulating and tumor‐infiltrating cDC2s and of IFNα and IFNλ1 by tumor‐infiltrating pDCs and cDC1s, respectively, compared to HD (increase in both proportion of positive DCs and total amount of cytokine secreted). Notably, in the case of cDCs, such features were linked to a better clinical outcome, as recently shown for cDC1s in breast cancer,^53^ revealing that when *in situ* activation of DCs could occur within the tumor microenvironment in patients it favors tumor immune control. Natural *in situ* activation of DCs could result from damage‐associated molecular patterns (DAMPs) released by tumor cells following immunogenic cell death,^61^ activation of the cGAS‐cGAMP‐STING pathway by cytosolic DNA,^62^ complexes of self‐DNA/RNA with cathelicidin (LL37)^63^, ^64^ or mitochondrial DNA^65^ as uncovered for pDCs, or from soluble factors released by tumors cells such as alarmin IL‐33 which was shown to affect DC maturation.^66^ Such DC‐activating signals remain to be identified in melanoma.

Our investigations also allowed uniquely deciphering the interrelations within DC subsets that shaped clinical outcome. We unveiled through correlation matrix performed between DC subsets’ features that melanoma drastically tuned interrelations between DC subsets. Indeed, perturbed interrelations between the three DC subsets were observed in both blood and tumor of melanoma patients compared to HD for the frequency, the basal activation status, and the response to TLR triggering. Overall, positive interrelations between cDC2s, pDCs and cDC1s were lost in both blood and tumor of melanoma patients, whereas new interactions emerged within tumor microenvironment especially between pDCs and cDC1s. Such perturbations may participate to melanoma escape from immune control, as inter‐DC cross‐talks are crucial for effective antitumor immune responses.^67^ cDC1s predominantly prime CD8 T cells, cDC2s mostly activate CD4 T cells that provide help to maximise CTL responses, whereas pDCs are specialised in immune regulation but could also provide critical licensing signals to cDCs. Indeed, local production of type I IFN can drive the maturation and activation of cDCs, triggering upregulation of co‐stimulatory molecules and favoring their antigen‐presenting functions. Type I IFN signalling in cDC1s promotes their accumulation at the tumor site, enhances their immunogenic maturation as well as their cross‐presentation ability^68^, ^69^ and improves type III IFN secretion^70^ together with the trans‐presentation of IL‐15, which promotes proliferation of CTLs.^71^ Conversely, IFNλ1 can impact features of pDCs and potentiate IFNα production.^72^ cDC1s require contributions from other DC subsets for an optimal CTL response.^73^ Therefore, inter‐DCs’ synergistic cooperation is crucial for efficient cross‐priming of antitumor responses^74^ and should be considered for the development of optimal DC‐based immunotherapies to achieve robust antitumor immunity and maximal clinical success.

Despite tumor‐induced immune‐subversion, our data together with available literature highlight that DC subsets are still able to respond to TLR triggering and suggest that the proper activation of DC subsets may participate in the triggering of protective immunity. We previously described that imiquimod (TLR7‐L) treatment in melanoma‐bearing mouse models can reverse the tolerogenic activity of tumor‐infiltrating pDCs, triggering their cytotoxic functions and impeding tumor vascularisation.^46^ In a clinical trial, vaccination of melanoma patients with TAA‐loaded autologous pDCs drove antigen‐specific CD8^+^ and CD4^+^ T‐cell responses and improved OS^49^ showing the reversibility of pDCs subversion by tumor cells. The activation of human cDC1s by TLR‐L (polyI:C) in humanised mice was associated with cross‐presentation and induction of CTL responses.^75^ Current clinical trials in melanoma patients are using combination therapies based on DC vaccines together with ICB to increase patient responsiveness.^76^ It has been reported from a phase II clinical trial that intradermal administration of CpG_B_ and GM‐CSF around the primary tumor excision site triggered the concerted activation of pDCs and cDCs together with the recruitment of BDCA3^+^ cDC1s in sentinel lymph nodes in a type I IFN‐dependent manner responsible for cross‐priming T‐cell responses.^77^ Promisingly, the co‐delivery of αGalCer and tumor antigens to cDC1s using nanoparticle‐based vaccine covered with anti‐CLEC9A antibodies promotes antitumor responses both *in vivo* in mouse model and *ex vivo* from PBMC of melanoma patients.^78^ Such new knowledge on DC biology suggests that past and ongoing DC vaccination protocols used in clinical trials to date, based mostly on monocyte‐derived DCs, may not be ideal, and our study provides exciting new therapeutic tracks to use or modulate DCs for cancer therapy.

Interestingly, the importance of DC subsets for therapeutic response to immune checkpoint blockers (ICB) has been demonstrated in several studies. Using mice deficient for cross‐presentation of cell‐associated antigens, it has been highlighted that Batf3‐dependent DCs (equivalent to human cDC1s) are essential for the response to therapy with anti‐CD137 combined to anti‐PD1^54^ antibodies. Furthermore, activation of CD103^+^ DCs at the tumor site enhances tumor responses to PDL1 and BRAF inhibition.^29^ In melanoma patients, it has been shown that the proportion of tumor‐infiltrating cDC1s was higher in patients responding to anti‐PD1 therapy.^56^ TLR9 agonists, targeting pDCs, could also improve the therapeutic potential of ICB.^79^ Thus, properly activated DC subsets revealed to be crucial for accurate elicitation of antitumor responses and response to immunotherapies.

DC subsets represent attractive candidates for therapeutic manipulation. Overall, our study brings new insights into the pathophysiologic role of DC subsets in melanoma and the prognostic impact of features of DCs on clinical outcome of patients, allowing a better understanding of the mechanisms of melanoma escape from immune surveillance. Elucidating the mechanisms of subversion of DC subsets by tumors is essential to manipulate or target these potent immune players and design new immunotherapeutic strategies. Exploiting the potencies of each DC subset to trigger appropriate immune responses together with efficient inter‐DC cross‐talks while avoiding their subversion is promising to achieve immune control of the tumor and improve clinical success. DC subsets are thus critical players to position in the therapeutic landscape of cancers.

## Methods

### Melanoma patients and control samples

This protocol conformed to the French Blood Service’s (EFS‐AuRA) Institutional Review Board and the ethics committee of Grenoble University Hospital (CHU‐Grenoble) and declared under the reference #DC‐2008‐787. Written informed consent was acquired from all participants prior to their participation in this study. Blood samples were obtained from Stage I‐IV melanoma patients (*n* = 17) and healthy donors (HD, *n* = 80). Peripheral blood mononuclear cells (PBMCs) were isolated using Ficoll‐Hypaque density gradient centrifugation (Eurobio, Les Ulis). Lymph node or cutaneous metastatic tumors were obtained from 27 melanoma patients (naïve of treatment by immunotherapies). Tonsils obtained from patients that underwent tonsillectomy (*n* = 9) were used as a tissue control. Tumor samples and tonsils were reduced to cell suspensions by enzymatic digestion with 2 mg mL^−1^ collagenase‐D (Roche, Boulogne‐Billancourt) 20 U mL^–1^ DNase (Sigma, Lyon) and mechanical disruption. The resulting cell suspensions were filtered and washed. Blood and tissue samples were biobanked and stored in liquid nitrogen at −196˚C. Clinical features of patients are reported in Supplementary tables 1 and 2.

### Flow cytometry

Frozen samples were thawed and stained in PBS 2% foetal calf serum (FCS) with several fluorochrome‐labelled anti‐human antibodies depending on the DC subset and analyses. We developed a novel multi‐parametric flow cytometry approach (11 colours) allowing depicting the 3 DC subsets simultaneously. The combination of the following surface markers allowed to define cDC1s, cDC2s and pDCs: CD11c, HLA‐DR (BD Biosciences, Le Pont de Claix), Lin (Biolegend, Paris), CD45, cDC1/BDCA1 (Beckman, Roissy), BDCA2, BDCA3 and BDCA4 (Miltenyi, Paris). We used the same fluorochrome for BDCA2 and BDCA3 antibodies because the corresponding DC subsets were distinguishable by different intensities of labelling. To assess the basal activation status, CD86 (BD), CD40 and CD80 (Beckman) fluorochrome‐labelled anti‐human antibodies were used. Stained cells were then analysed using LSRII Flow Cytometer and FACSDiva software v.8 (BD). Isotype controls were used to differentiate positive cells from nonspecific background staining (CD45^+^ cells also served to determine the threshold of positivity). Dead cells were excluded with live and dead staining. To ensure quality control during the study, we performed a standardisation of the fluorescence intensities using cytometer setup and tracking beads (CST) (BD).

### Functional analysis of circulating and tumor‐infiltrating DCs in response to TLR triggering

#### Intracellular cytokine staining within DC subsets

Cultures were performed in RPMI‐1640/GlutaMAX (Invitrogen, Courtaboeuf) supplemented with 1% non‐essential amino acids, 100 µg mL^−1^ gentamicin, 10% FCS (Invitrogen) and 1 mmol L^−1^ sodium pyruvate (Sigma) (complete medium). For intracellular cytokine characterisation, cells from the different samples were cultured at 4x10^6^ cells mL^–1^ for 5h with or without TLR ligands alone or mixed together, including polyinosinic‐polycytidylic acid (polyI:C, TLR3L, 30µg mL^−1^), Resiquimod (R848, TLR7/8L, 1µg mL^−1^) and Class‐A CpG oligonucleotide ODN‐2336 (CpGA, TLR9L, 1µM (Invivogen, Toulouse). 1µg mL^−1^ of Brefeldin A (BD) was added after 1h. Later on, cells were stained for surface markers allowing to define cDC1s, cDC2s and pDCs (CD11c, HLA‐DR (BD), Lin, CD45 (Biolegend), cDC1/BDCA1 (Beckman), BDCA2 and BDCA3 (Miltenyi)) and then fixed and permeabilised according to the manufacturer’ instructions (BD Biosciences). Intracellular cytokine staining was then performed using the fluorochrome‐labelled anti‐human TNF⍺, Il‐12p40/70 (BD), IFN⍺ (Miltenyi) antibodies and anti‐human IFNλ1 (Novus, Abingdon) antibody stained with mix‐*n*‐stain CF488 (Biotium, Fremont). Analyses were done by flow cytometry using LSRII Flow Cytometer and FACSDiva software v.8.

#### Maturation of DC subsets and cytokine secretion

To study the maturation of DCs after TLR‐L stimulation, samples were cultured in complete RPMI medium at 4 × 10^6^ cells mL^–1^ for 21 h with or without a single or a mixture of the TLR ligands previously described. The potential upregulation of the co‐stimulatory molecules on the 3 DC subsets was then investigated using the fluorochrome‐labelled anti‐human CD86 (BD), CD40 and CD80 (Beckman) antibodies, together with the antibodies allowing to depict the 3 DC subsets (CD11c, HLA‐DR (BD), Lin, CD45 (Biolegend), cDC1/BDCA1 (Beckman), BDCA2 and BDCA3 (Miltenyi)). Analyses were performed using LSRII Flow Cytometer and FACSDiva software v8 (BD). PBMCs and melanoma metastatic tumor supernatants were harvested after 20h of culture, and IL12p70, IFNα, IFNβ, IFNλ1 and IFNλ2 cytokine secretions were measured by LUMINEX technology using MAGPIX®200 Instrument with xPONENT® software (Bio‐Rad, Cressier).

### Statistics

Statistical analyses were performed using the Mann–Whitney and the Wilcoxon nonparametric *U*‐tests with Bonferroni correction, and the Kruskal–Wallis and the Friedman nonparametric test with post hoc Dunns’ multiple comparison test using GraphPad Prism software (San Diego). The data are shown as means, and significance threshold was placed at *P* < 0.05. Survival analyses (Cox regression, Kaplan‐Meier), correlations, heat maps and principal component analysis (PCA) were performed using the survival, GGally, gplots, ggplot2, ggbiplot, MissMDA and FactoMineR packages of the R i386 software version 3.6.2.

## Conflicts of interest

The authors declare no conflict of interest.

## Author Contribution

**Eleonora Sosa Cuevas:** Conceptualization; Data curation; Formal analysis; Investigation; Methodology; Writing‐original draft. **Laurissa Ouaguia:** Data curation; Formal analysis; Methodology; Writing‐review & editing. **Stephane Mouret:** Formal analysis; Resources; Writing‐review & editing. **Julie Charles:** Formal analysis; Resources; Writing‐review & editing. **Florence De Fraipont:** Formal analysis; Resources; Writing‐review & editing. **Olivier Manches:** Formal analysis; Methodology; Writing‐review & editing. **Jenny Valladeau‐guilemond:** Conceptualization; Methodology; Validation; Writing‐review & editing. **Nathalie Bendriss‐Vermare:** Conceptualization; Funding acquisition; Methodology; Validation; Writing‐review & editing. **Laurence Chaperot:** Formal analysis; Project administration; Validation; Writing‐review & editing. **Caroline Aspord:** Conceptualization; Data curation; Formal analysis; Funding acquisition; Investigation; Methodology; Project administration; Resources; Supervision; Validation; Writing‐original draft; Writing‐review & editing.

## Supporting information

Supplementary Material
