# Supplementary material for: BDCA1+ cDC2s, BDCA2+ pDCs and BDCA3+ cDC1s reveal distinct pathophysiologic features and impact on clinical outcomes in melanoma patients
Source: Clin Transl Immunology. 2020 Nov 24;9(11):e1190. doi: 10.1002/cti2.1190 (PMC7684973; doi:10.1002/cti2.1190)
Supplement: Supplementary file 1 — Supplementary Material [file CTI2-9-e1190-s001.pdf]

Supplementary Figure legends

__Supplementary Figure 1__. **Multi-parametric flow cytometry approach to simultaneously depict BDCA1+ DC/cDC2s, BDCA2+ DC/pDCs and BDCA3+ DC/cDC1s**

(a) DC subsets' gating strategy. This technique allows the study of the three major human DC subsets in the same sample by using specific surface markers. FSC-A and SSC-A parameters allowed to first exclude cell debris. Single cells were then gated and dead cells excluded with Live and Dead cell staining. Within CD45+ cells, Lin-HLA-DR+ cells were then selected and further distinguished using CD11c and BDCA2+BDCA3 markers. Within CD45+Lin-HLA-DR+CD11c+ BDCA2-BDCA3- cells, cDC2s were finally depicted using BDCA1 positive labeling. Thus, BDCA1+ cDC2s were defined as CD45+ Lin- HLA-DR+ CD11c+ BDCA1+, BDCA2+ pDCs were depicted as CD45+ Lin- HLA-DR+ CD11c- BDCA2+, and BDCA3+ cDC1s highlighted as CD45+ Lin- HLA-DR+ CD11c+ BDCA3+. Representative flow cytometry plots from a HD. **(b)** Representative dot plots depicting the three major DC subsets in blood (healthy donors, patients) and tissues (non-tumor and metastatic melanoma).

__Supplementary Figure 2__. **Impact of disease stage and tumor location, and inter-relations between DC subsets in blood and tumor of melanoma patients compared to controls**

PBMC and tumor-infiltrating cells from melanoma patients together with PBMC from HD and control tissues were labelled with specific antibodies allowing depicting the three DC subsets and submitted to flow cytometry analysis. **(a)** Frequencies of the three circulating DC subsets were compared between early stage I-II (grey symbols, n=10) and late stage III-IV (black symbols, n=7) patients. **(b)** Among tumor-infiltrating cells, samples were separated according to the location of the tumor. Frequencies of the three DC subsets in lymph node (grey symbols, n=12) and cutaneous (black symbols, n=7) metastases of melanoma patients. Results are expressed as percentages of positive cells. Bars indicate mean. *P*-values were calculated using Mann-Whitney test. **(c)** Comparative frequencies of BDCA1+ cDC2s (triangles), BDCA2+ pDCs (circles) and BDCA3+ cDC1s (diamonds) within alive $CD45^{+}$ cells in the blood of healthy donors (HD, n=22) and patients (Pt, n=17), non-tumor tissue (tonsils, n=9) and tumor infiltrate of melanoma patients (n=23). Bars indicate mean. *P*-values were calculated using

Wilcoxon (dashed lines) and Friedman with post hoc Dunns' multiple comparison test (straight lines) non parametric tests. *$P \leq 0.05$,** $P \leq 0.01$,***$P \leq 0.001$. **(d)** Pie charts representing the relative proportions of each DC subsets in each group (BDCA1+ cDC2s, blue; BDCA2+ pDCs, red; BDCA3+ cDC1s, green). **(e)** Relative proportions of each DC subsets compared to all DCs in patients' blood (n=17), lymph nodes (n=12) and cutaneous (n=7) metastases. Bars indicate mean. *P*-values were calculated using Mann-Whitney test. **(f)** Comparative PFS (from diagnosis time) of patients with low or high circulating pDC frequencies. Groups were separated using the median percentage of circulating pDCs within CD45+ cells (0.196%) (n=8 or 9 patients/group). Comparison using Log-rank test.

Supplementary Figure 3. Circulating and tumor-infiltrating DCs in patients can be differentiated from healthy donors by their frequencies and basal activation status

The expression of the co-activation molecules CD80, CD40, and CD86 on the three DC subsets was analyzed by flow cytometry on PBMCs and tumor infiltrating cells of melanoma patients, HD or non-tumor tissue controls, and correlated with the clinical parameters of the corresponding patients. **(a)** Representative flow cytometry dot plots of CD40 expression by BDCA1+ cDC2s (blue), BDCA2+ pDCs (pink), and BDCA3+ cDC1s (green)of healthy donor or patient blood and tumor infiltrate of melanoma patient. **(b)** Comparative expression levels (MFI) of the co-stimulatory molecules CD80, CD40 and CD86 were analyzed on the three CD80-, CD40- or CD86-expressing DC subsets by flow cytometry on healthy donor (HD, n=19 to 22) and patient (Pt, n=9 to 17) blood, tumor infiltrate of melanoma patients (n=23 or 24) and non-tumor tissue (tonsils, n=8 or 9). Only samples were the proportions of CD80-, CD40- or CD86-expressing cells were above 2% were kept for this analysis. Bars indicate mean. *P*-values were calculated using Mann-Whitney (dashed lines) and Kruskal-Wallis with post hoc Dunns' multiple comparison test (straight lines) non parametric tests. *$P \leq 0.05$, **$P \leq 0.01$, ***$P \leq 0.001$. **(c, d)** Comparative expression levels of the co-stimulatory molecule CD86 on the three DC subsets **(c)** from the blood of early stage I-II (grey symbols, n=10) and late stage III-IV (black symbols, n=7) patients and **(d)** in lymph nodes (n=10) and cutaneous (n=7) metastases. Results are expressed as percentages of positive cells within the corresponding DC subset. Bars indicate mean.

P-values were calculated using Mann-Whitney test. **(e)** Comparative PFS (from sampling time – left panel) and OS (from diagnosis time – right panel) of patients with low or high tumor-infiltrating CD80+ or CD40+ pDCs respectively. Groups were separated using the median percentage of tumor-infiltrating CD80+ (4.04%) and CD40+ (71.67%) pDCs (n=7 to 11 patients/groups). **(f)** Comparative OS (from sampling time) of patients with low or high circulating CD40+ cDC1s. Groups were separated using the median percentage of circulating CD40+ cDC1s (90.10%) (n=8 or 9 patients/group). **(e-f)** Comparison using Log-rank test. **(g)** Graph of variables relative to the PCA shown on Figure 3B (left panel : all DC subsets, right panel: cDC2s. **(h)** PCA based on DC subsets co-stimulatory molecules expression (CD80, CD40, CD86) for BDCA2+ pDCs and BDCA3+ cDC1s (including their graph of variables). **(i)** Spearman correlations table showing *r* factors with their significant *P*-values (<0.05) after Bonferroni–Holm's correction in each sample group (HD blood, patient blood, tumor infiltrate).

Supplementary Figure 4. The maturation status of circulating and tumor-infiltrating BDCA1+ cDC2s and BDCA2+ pDCs from melanoma patients upon TLR stimulation dictated their overall survival

Cell suspensions from blood (HD, n=17; Pt, n=15) or tumor infiltrates (Pt, n=12) were stimulated or not for 21h with or without TLR ligands (polyI:C, R848 or CpG_A) alone or mixed together (mix). The expression levels of the co-stimulatory molecules CD80, CD40 and CD86 were measured on BDCA1+ cDC2s and BDCA2+ pDCs using flow cytometry and correlated with the clinical parameters of the corresponding patients. **(a)** Comparative expression levels (MFI) of the co-stimulatory molecules CD80, CD40 and CD86 were analyzed on CD80-, CD40- or CD86-expressing DC subsets by flow cytometry Bars indicate mean. Stars indicate significant differences compared to the control condition without stimulation (-) within each group. *P*-values were calculated using Mann-Whitney (dashed lines) and Kruskal-Wallis with post hoc Dunns' multiple comparison test (stars) non parametric tests. $*P \leq 0.05$, $***P \leq 0.001$. (**b**) Comparative PFS (from sampling (left) or diagnosis (right) time) of patients with low or high tumor-infiltrating CD40+ cDC2s and CD80+ pDCs in absence of TLR stimulation. Groups were separated using the median percentage of CD40+ cDC2s

(93.27%) and CD80+ pDCs (16.48%) from unstimulated tumor infiltrates (n=4-6 patients/group). **(c-d)** Comparative expression levels of the co-stimulatory molecule CD86 on cDC2s and pDCs **(c)** from the blood of early stage I-II (grey symbols, n=8 or 9) and late stage III-IV (black symbols, n=4 to 6) patients, and **(d)** in lymph nodes (n=6) and cutaneous (n=3) metastases. Results are expressed as percentages of positive cells within the corresponding DC subset. Bars indicate mean. *P*-values were calculated using Mann-Whitney (dashed lines) and Kruskal-Wallis with post hoc Dunns' multiple comparison test (full lines) non parametric tests. **(e)** Comparative PFS (from diagnosis time) of patients with low or high CD80+ or CD40+ cDC2s after R848 stimulation and CD86+ pDCs after CpG_A stimulation of circulating cells. Groups were separated using the median percentage of circulating CD80+ (19.83%) and CD40+ (48.34%) cDC2s after R848 stimulation, and CD86+ pDCs (40.15%) after CpG_A stimulation (n=5 or 6 patients/groups). **(f)** Comparative OS (from sampling time) of patients with low or high CD86+ cDC2s after mix TLR-L mix stimulation of tumor-infiltrating cells. Groups were separated using the median percentage of CD86+ cDC2s after mix TLRL stimulation (70.86%) (n=5 or 6 patients/group). **(e-f)** Comparison using Log-rank test.

<u>Supplementary Figure 5</u>. **Expression of co-stimulatory molecules by circulating and tumor-infiltrating cDC2s and pDCs upon TLR triggering segregated them from healthy donors and impacted the clinical outcome of melanoma patients**

Cell suspensions from blood (HD, n=17; Pt, n=15) or tumor infiltrates (n=14) were stimulated or not for 21h with or without TLR-L (polyI:C, R848 or CpGA) alone or mixed together (mix). The expression of the co-stimulatory molecules CD80, CD40 and CD86 were measured on cDC2s and pDCs using flow cytometry and correlated with the clinical parameters of the corresponding patients. **(a)** Comparative OS (from diagnosis time) of patients with low or high CD80+ or CD40+ cDC2s and CD86+ pDCs after stimulation of PBMC with R848 or CpGA respectively. Groups were separated using the median percentage of CD80+ cDC2s (19.84%), CD40+ cDC2s (48.34%) and CD86+ pDCs (40.15%) after stimulation with R848 and CpGA respectively (n=5 or 6 patients/group). **(b)** Comparative PFS and OS (from sampling time) of patients with low or high CD86+ cDC2s upon stimulation of tumor-infiltrating cells with R848 or mix TLR-L. Groups were separated using the

median percentage of CD86+ cDC2s following stimulation with a mixture of TLR-L (70.86%) or R848 (66.07%) (n=4-5 patients/group). **(c)** Comparative OS (from diagnosis (left) or sampling (right) time) of patients with low or high proportions of pDCs positive for CD80 or CD86 following stimulation of tumor-infiltrating cells with CpGA or mix TLR-L. Groups were separated using the median percentage of CD80+ pDCs (49.23%) or CD86+ pDCs (54.69%) (n=4 to 6 patients/group) upon stimulation with mix TLR-L or CpGA. **(a-c)** Comparison using Log-rank test.

Supplementary Figure 6. The inter-relations between co-stimulatory molecules-expressing DC subsets after TLR-triggering were altered in the context of melanoma, and impacted on patients' clinical outcome

Cell suspensions from blood (HD, n=17; Pt, n=15) or tumor infiltrates (Pt, n=12) were stimulated or not for 21h with or without TLR ligands (polyI:C, R848 or CpG_A) alone or mixed together (mix). The expression levels of the co-stimulatory molecules CD80, CD40 and CD86 were measured on BDCA1+ cDC2s and BDCA2+ pDCs using flow cytometry and correlated between them. cDC1s were removed from the analysis due to cell scarcity after 20h culture. **(a)** Heat map based on co-stimulatory molecule expressions by cDC2s and pDCs with or without a 20h stimulation with TLR-L (polyI:C, R848 or CpG_A) in each sample type (HD blood, patient blood and tumor infiltrate). **(b)** PCA and graph of variables (in HD blood, patient blood and tumor infiltrate) of co-stimulatory molecule expression (CD80, CD40 and CD86) by cDC2s and pDCs after TLR stimulation (polyI:C, R848 or CpGA). **(c)** PCA based on co-stimulatory molecules expression (CD80, CD40 and CD86) by cDC2s and pDCs after a 20h stimulation with TLRL in each sample group (HD blood, patient blood, tumor infiltrate) together with their corresponding graph of variables. **(d)** Correlation matrix between co-stimulatory molecule-expressing cDC2s and pDCs after a 20h TLRL stimulation (polyI:C, R848 or CpG_A alone or mixed together) in HD blood (left panel), patient blood (right panel) and tumor infiltrate (bottom panel). Spearman correlations with significant *P*-values (< 0.05) after Bonferroni–Holm's correction are circled in black. "Stim." corresponds to condition without ex-vivo TLR triggering, and "X" stands for percentage. **(e)** Table summarizing the intra-DCs and inter-DCs spearman correlations showing *r* factors with their significant *P*-values (< 0.05) after Bonferroni–

Holm's correction in each sample group (HD blood, patient blood, tumor infiltrate). Only parameters for which significant correlations were observed in at least one of the groups are shown.

Supplementary Figure 7. **Intracellular IL-12p40/p70, IFNα and IFNλ1 cytokine analysis within circulating and tumor-infiltrating DC subsets following stimulation with TLRL**

Cell suspensions from blood (HD, n=15, open circles; Pt, n=17, filled circles) or tumor infiltrates (Pt, n=16, filled triangles) were stimulated for 5h with or without TLR-L (polyI:C, R848 or CpG_A) alone or mixed together, and the production of cytokines was evaluated by intracellular cytokine staining using flow cytometry. **(a)** Representative flow cytometry dot plots of healthy donor or patient blood and tumor infiltrate of melanoma patient displaying intracellular staining for IL12p40/70, IFNα and IFNλ1 within respectively BDCA1+ cDC2s (blue, top panels), BDCA2+ pDCs (pink, middle panels), and BDCA3+ cDC1s (green, lower panels) following stimulation or not with R848 or polyI:C as indicated. **(b)** Comparative proportion of cytokine-expressing cDC2s, pDCs and cDC1s from the blood of early stage I-II (white symbols, n=10) and late stage III-IV (black symbols, n=7) patients. Results are expressed as percentages of positive cells within the corresponding DC subset. Bars indicate mean. *P*-values were calculated using Mann-Whitney (dashed lines) and Kruskal-Wallis with post hoc Dunns' multiple comparison test (full lines) non parametric tests.

Supplementary Figure 8. **The inter-relations between cytokine-expressing DC subsets after TLR triggering s were altered in the context of melanoma, and impacted on patients' clinical outcome**

Cell suspensions from blood (HD, n=15, open circles; Pt, n=17, filled circles) or tumor infiltrates (Pt, n=16, filled triangles) were stimulated for 5h with or without TLR-L (polyI:C, R848 or CpG_A) alone or mixed together. The production of cytokines was evaluated by intracellular cytokine staining using flow cytometry and correlated with the clinical parameters of the corresponding patients and also between them. **(a)** Comparative PFS (from sampling time) of patients with low or high TNFα+ cDC2s and IFNα+ pDCs after stimulation of tumor-infiltrating cells with R848. Groups were separated using the median percentage of TNFα+ cDC2s (15.07%) and IFNα+ pDCs (6.72%) after R848 stimulation

(n=2 to 4 patients/group). Comparison using Log-rank test. **(b)** Comparative proportion of cytokine-expressing cDC2s, pDCs and cDC1s in lymph nodes (n=7) and cutaneous (n=3) metastases. Results are expressed as percentages of positive cells within the corresponding DC subset. Bars indicate mean. *P*-values were calculated using Mann-Whitney (dashed lines) and Kruskal-Wallis with post hoc Dunns' multiple comparison test (full lines) non parametric tests. **(c)** Graph of variables relative to the PCA displayed on Figure 7D. **(d)** PCA based on intra-cellular cytokine expressions (IL-12p40/p70, IFNα, IFNλ1, TNFα) by cDC2s, pDCs and cDC1s upon 5h stimulation with or without TLRL in each sample group (HD blood, patient blood, tumor infiltrate). **(e)** Correlation matrix between cytokine-expressing DC subsets after 20h TLR stimulation (polyI:C, R848 or CpG_A alone or mixed together) in HD blood (left panel), patient blood (right panel) and tumor infiltrate (bottom panel). Spearman correlations with significant *P*-values (<0.05) after Bonferroni–Holm's correction are circled in black. **(f)** Table summarizing the intra-DCs and inter-DCs spearman correlations showing *r* factors with their significant *P*-values (<0.05) after Bonferroni–Holm's correction in each sample group (HD blood, patient blood, tumor infiltrate). Only parameters for which significant correlations were observed in at least one of the groups are shown.

Supplementary Figure 9. High levels of IL12p70, IFNα and IFNβ secreted from circulating and tumor-infiltrating cells after TLR stimulation are linked with better overall survival of melanoma patients

Cell suspensions from blood (HD, n=18, open circles; Pt, n=15, filled circles) or tumor infiltrates (Pt, n=15, filled triangles) were stimulated for 20h with or without TLR-ligands (polyI:C, R848 or CpG_A) alone or mixed together. The culture supernatants were examined for the presence of IL-12p70, IFNα, IFNβ, IFNλ1 and IFNλ2 by Luminex technology, and correlated with the clinical parameters of the corresponding patients. . **(a-b)** Comparative amounts of IL-12p70, IFNα, and IFNλ1 **(a)** from the blood of early stage I-II (white symbols, n=9) and late stage III-IV (black symbols, n=6) patients, and **(b)** in lymph nodes (n=7) and cutaneous (n=3) metastases. Bars indicate mean. *P*-values were calculated using Mann-Whitney (dashed lines) and Kruskal-Wallis with post hoc Dunns' multiple comparison test (full lines) non parametric tests. **(c)** Comparative OS (from diagnosis time) of patients

with low or high IL12p70, IFNα or IFNβ secretions from circulating immune cells in absence of stimulation or after stimulation with R848 or mix TLR-L as indicated. Groups were separated using the median of IFNα levels without stimulation (70.19pg/mL), the median of IL12p70 (39.05pg/mL) or IFNβ (307.63pg/mL) levels after mix TLR-L stimulation, or the median of IFNα levels after R848 stimulation (1303.02pg/mL) of circulating immune cells (n=6 or 9 patients/group). **(d)** Comparative OS (from diagnosis time) of patients with low or high IL12p70, IFNα or IFNβ secretions from tumor-infiltrating cells in absence of stimulation or after R848 stimulation as indicated. Groups were separated using the median of IFNα levels without stimulation (70.19pg/mL) (n=7 patients/group) or the median of IL12p70 (32.95pg/mL), IFNβ (193.76pg/mL) or IFNα (1218.37pg/mL) levels after R848 stimulation (n=3 or 4 patients/group). **(c-d)** Comparison using Log-rank test **(e)** Heat map of cytokine secretions (IL-12p70, IFNα, IFNβ, IFNλ1, IFNλ2) with or without stimulation with a mixture of TLR-L (polyI:C, R848 and CpG_A) in each sample type (HD blood, patient blood and tumor infiltrate).

Supplementary Figure 10. The inter-relations between cytokine secretions from circulating and tumor-infiltrating cells after TLR triggering were disturbed in melanoma patients

Cell suspensions from blood (HD, n=16, open circles; Pt, n=15, filled circles) or tumor infiltrates (Pt, n=14, filled triangles) were stimulated for 20h with or without TLR-ligands (polyI:C, R848 or CpG_A) alone or mixed together. The culture supernatants were examined for the presence of IL-12p70, IFNα, IFNβ, IFNλ1 and IFNλ2 by Luminex technology, and levels were correlated between them. **(a)** PCA based on secreted cytokine levels (IL-12p70, IFNα, IFNβ, IFNλ1, IFNλ2) upon 20h stimulation with or without TLRL in each sample group (HD blood, patient blood, tumor infiltrate). **(b)** Correlation matrix between secreted cytokines in response to a 20h stimulation with TLRL (polyI:C, R848 or CpG_A alone or mixed together) in HD blood (left panel), patient blood (right panel) and tumor infiltrate (bottom panel). Spearman correlations with significant *P*-values (< 0.05) after Bonferroni–Holm's correction are circled in black. **(c)** Table summarizing the spearman correlations showing *r* factors with their significant *P*-values (< 0.05) after Bonferroni–Holm's correction in each sample

group (HD blood, patient blood, tumor infiltrate). Only parameters for which significant correlations were observed in at least one of the groups are shown.

Supplementary Figure 11. The compass of immune deviations depicted in circulating and tumor-infiltrating DC subsets from melanoma patients

The figure integrates the main phenotypic and functional modulations of circulating and tumor-infiltrating DC subsets (cDC2s, top panel; pDCs, middle panel; cDC1s bottom panel) that we highlighted in the paper. For each DC subset, we studied the proportions within CD45+ cells (green pie), the basal activation status (orange pie), the co-stimulatory molecule expression with (+) or without (-) TLR triggering (pink pie), and the intra-cellular cytokine expression after (+) or not (-) TLR triggering (purple pie). Black circle: HD condition; Blue arrow: Pt blood; Red arrow: Tumor infiltrate. The length of the arrows is proportional to the features observed in HD conditions. This figure has been created with BioRender science illustration tool.

Supplementary Figure 12. Graphical summary of the findings

Suppl Figure 1

a

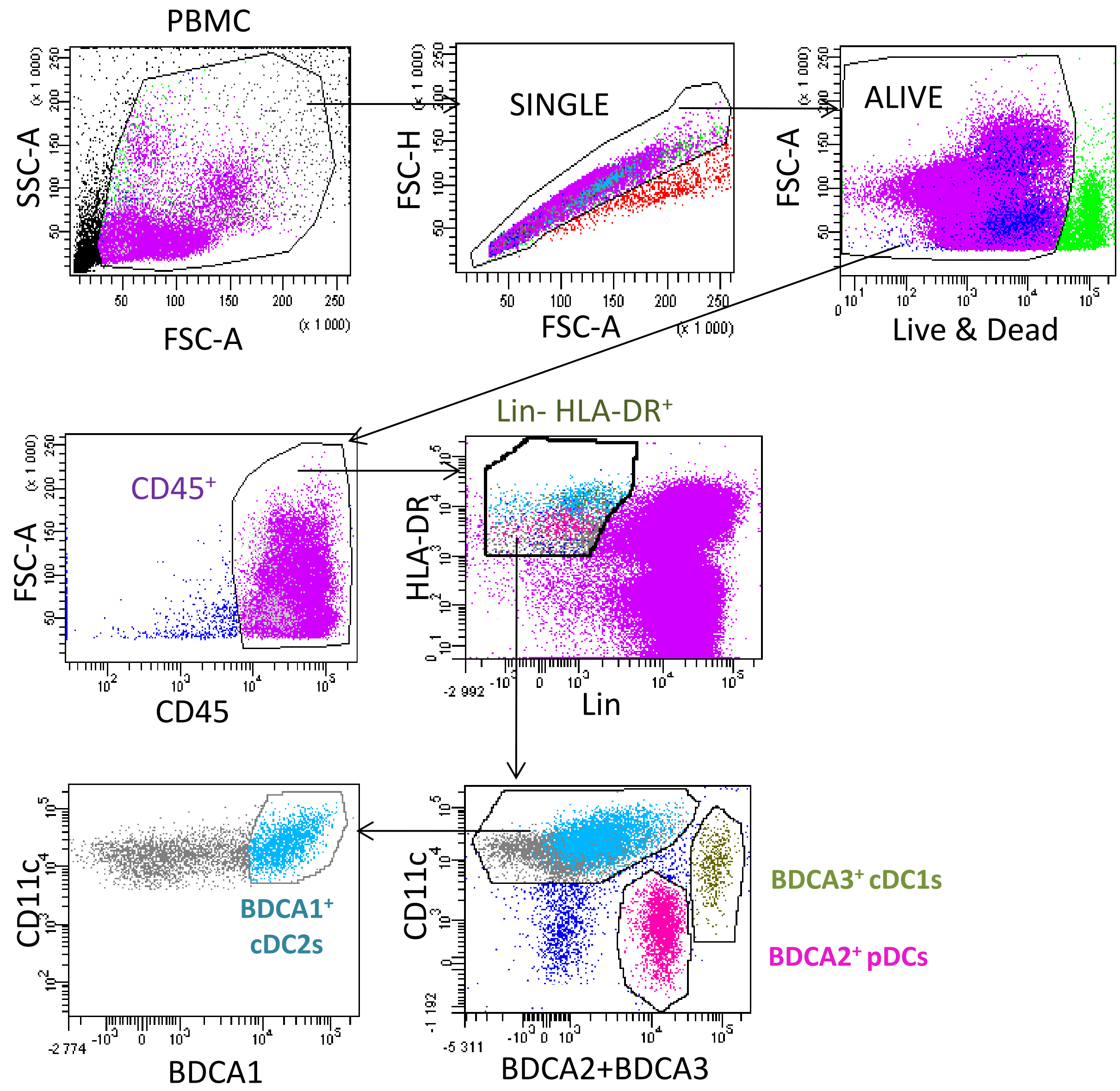

b

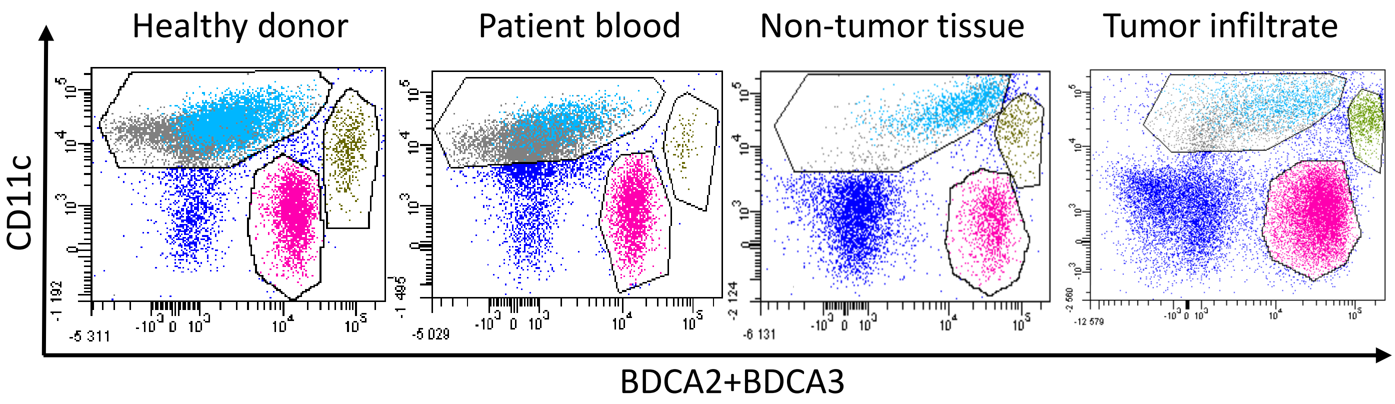

Suppl Figure 2

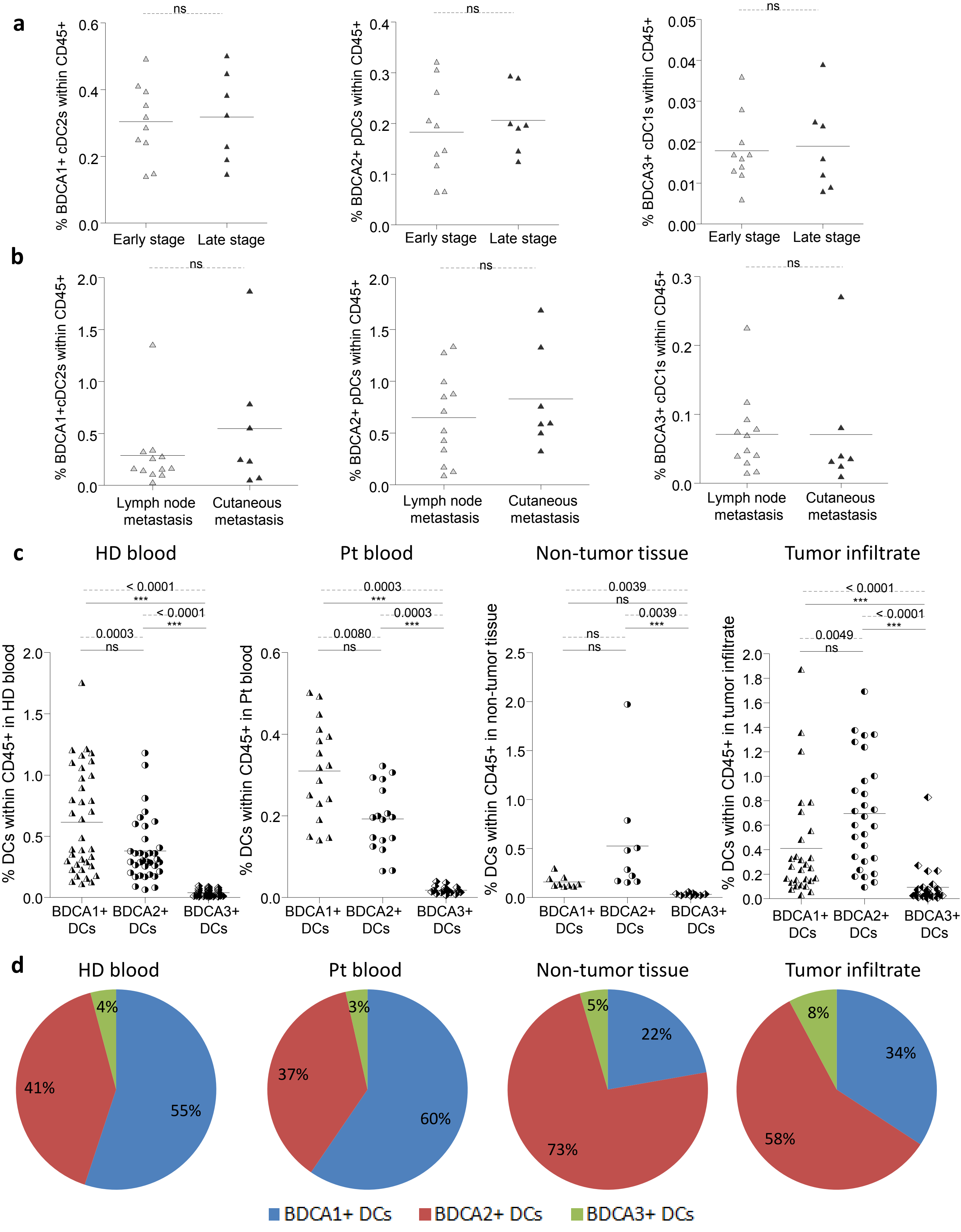

e

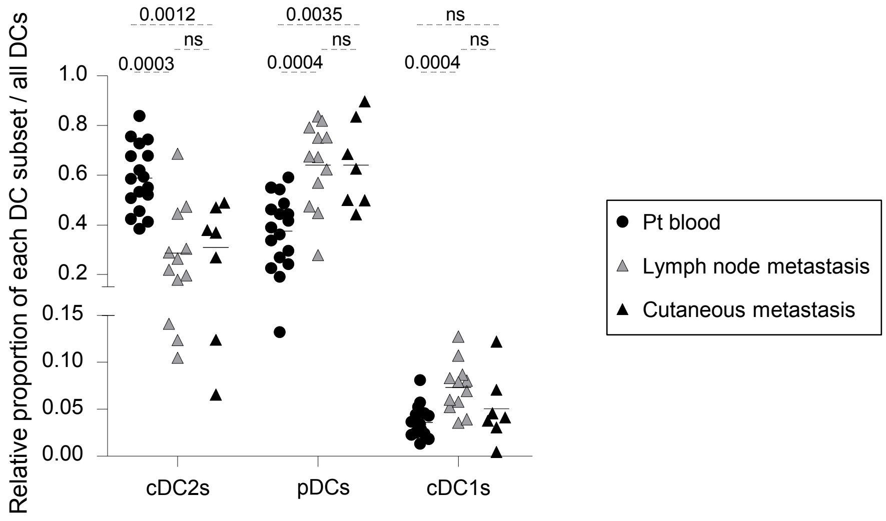
0.0012
ns
0.0003
0.0035
ns
0.0004
ns
ns
0.0004
Relative proportion of each DC subset / all DCs
1.0
0.8
0.6
0.4
0.2
0.15
0.10
0.05
0.00
cDC2s
pDCs
cDC1s
Pt blood
Lymph node metastasis
Cutaneous metastasis

f

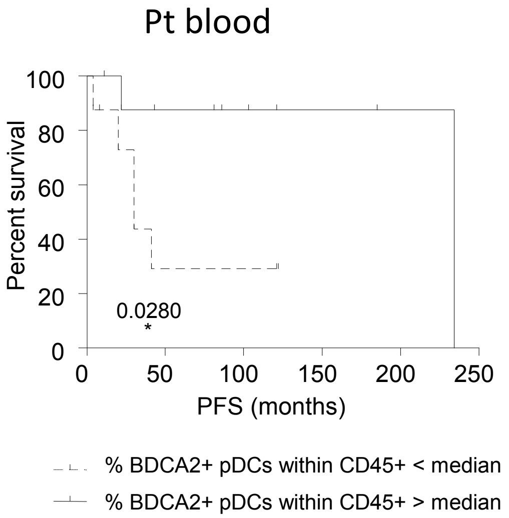
Pt blood
Percent survival
100
80
60
40
20
0
0.0280
*
0
50
100
150
200
250
PFS (months)
% BDCA2+ pDCs within CD45+ < median
% BDCA2+ pDCs within CD45+ > median

Suppl Figure 3

a

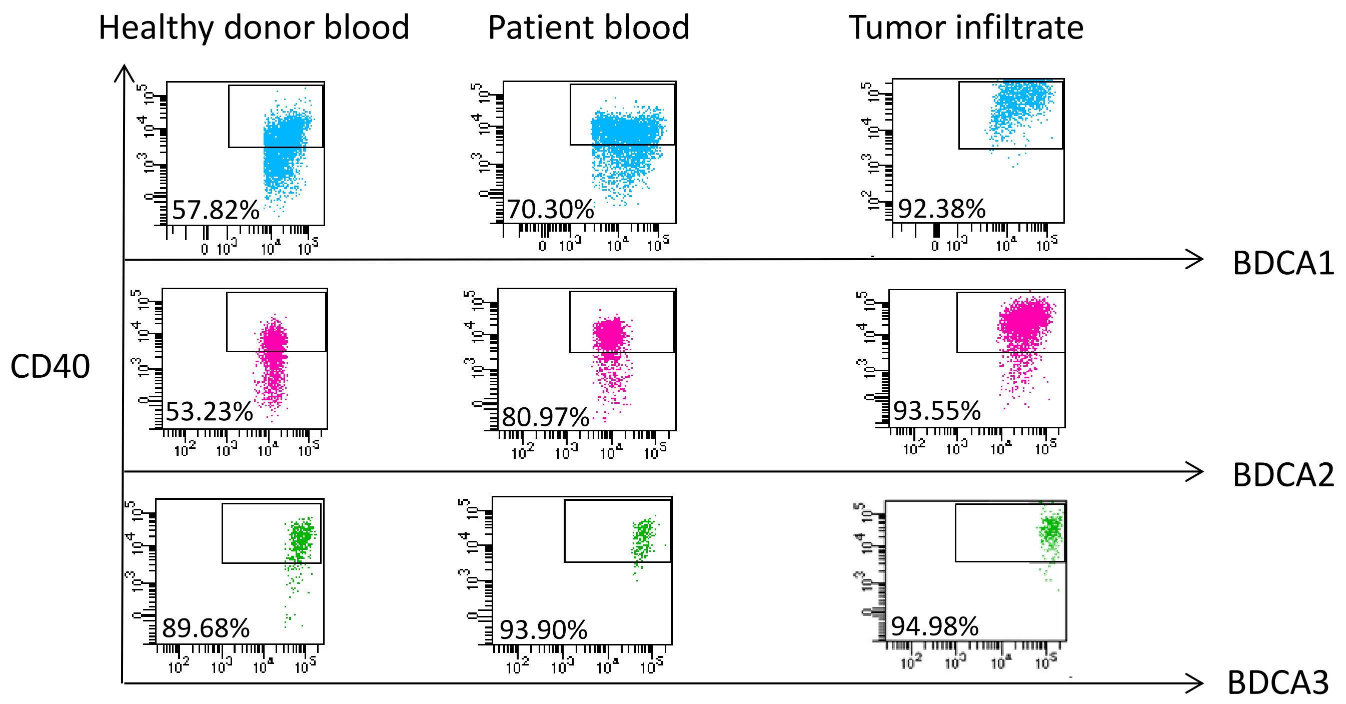

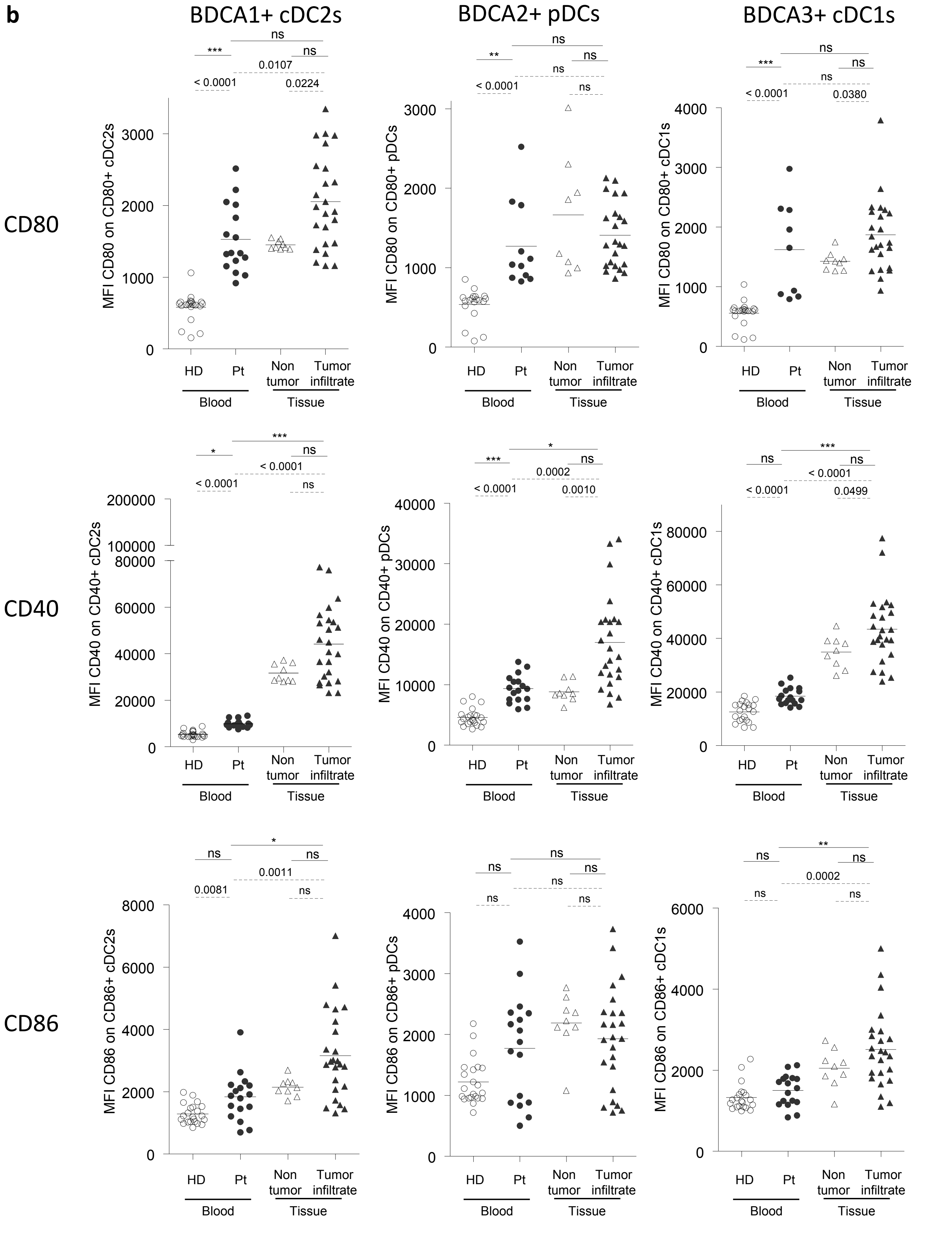

b
BDCA1+ cDC2s
BDCA2+ pDCs
BDCA3+ cDC1s
CD80
CD40
CD86
MFI CD80 on CD80+ cDC2s
MFI CD80 on CD80+ pDCs
MFI CD80 on CD80+ cDC1s
MFI CD40 on CD40+ cDC2s
MFI CD40 on CD40+ pDCs
MFI CD40 on CD40+ cDC1s
MFI CD86 on CD86+ cDC2s
MFI CD86 on CD86+ pDCs
MFI CD86 on CD86+ cDC1s
HD
Pt
Non tumor
Tumor infiltrate
Blood
Tissue
< 0.0001
0.0107
0.0224
0.0380
0.0002
0.0010
0.0499
0.0081
0.0011
ns
*
**
***

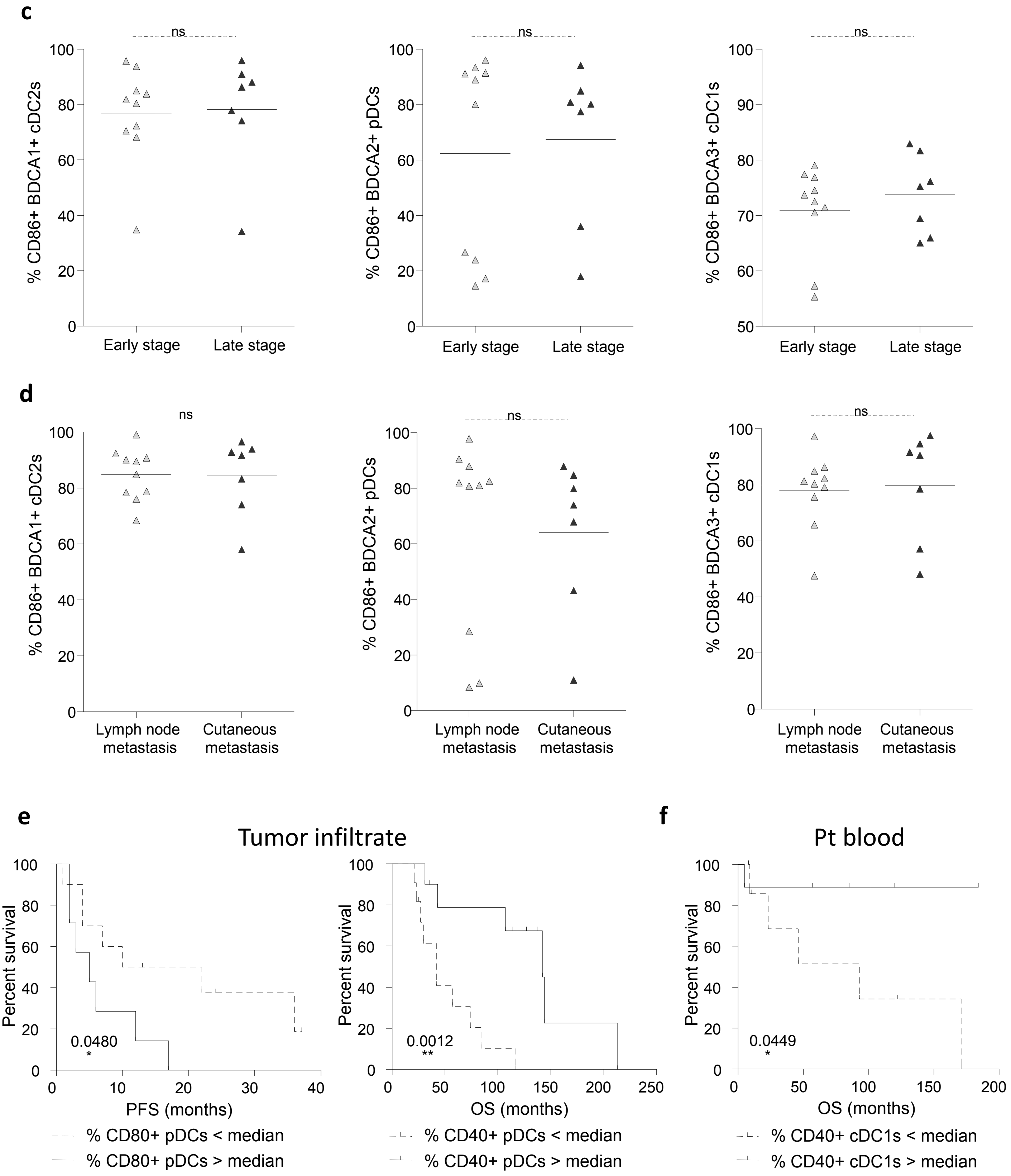

c
ns
% CD86+ BDCA1+ cDC2s
Early stage
Late stage
% CD86+ BDCA2+ pDCs
% CD86+ BDCA3+ cDC1s
d
Lymph node metastasis
Cutaneous metastasis
e
Tumor infiltrate
f
Pt blood
Percent survival
0.0480
*
PFS (months)
0.0012
**
OS (months)
0.0449
% CD80+ pDCs < median
% CD80+ pDCs > median
% CD40+ pDCs < median
% CD40+ pDCs > median
% CD40+ cDC1s < median
% CD40+ cDC1s > median

g

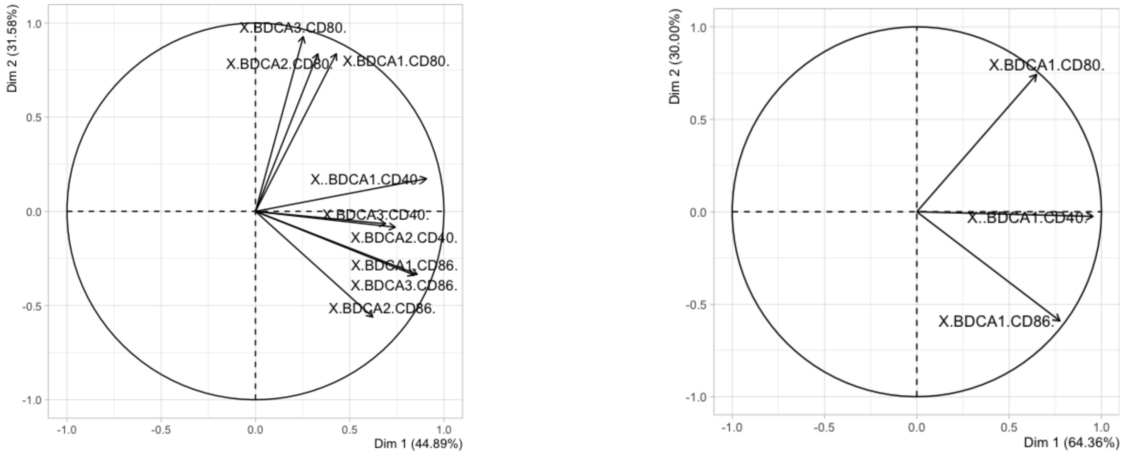

h

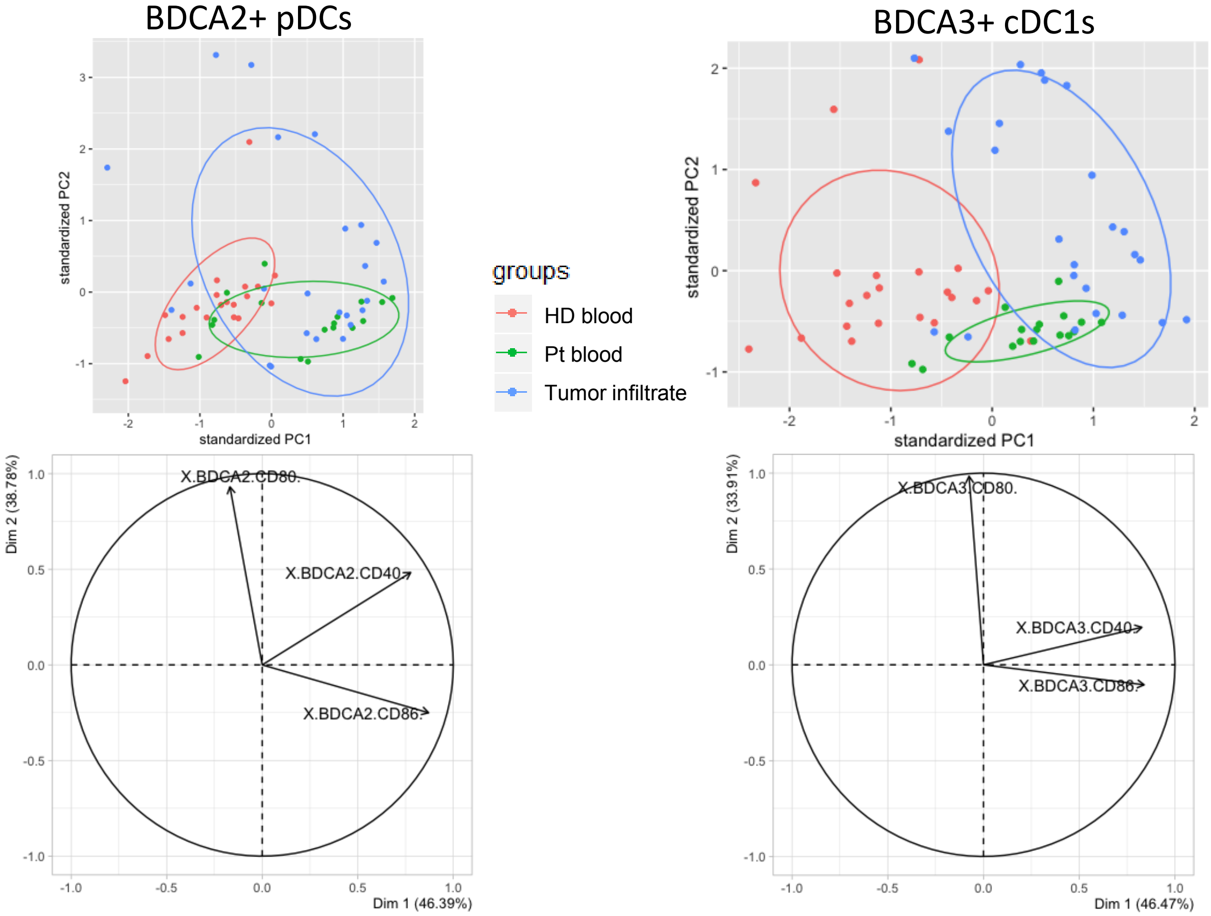

i

Variables		HD blood		Pt blood		Pt TIL	
		r	*p*	*r*	*p*	*r*	*p*
% BDCA1+ cDC2s CD40+	% BDCA1+ cDC2s CD86+	0.74	*0.004*	0.12	*1*	0.07	*1*
% BDCA1+ cDC2s CD80+	% BDCA2+ pDCs CD80+	0.83	*0.0001*	0.63	*0.220*	0.77	*0.0004*
	% BDCA2+ pDCs CD86+	0.33	*1*	-0.19	*1*	-0.64	*0.021*
	% BDCA3+ cDC1s CD80+	0.93	*<0.0001*	0.76	*0.014*	0.80	*0.0001*
% BDCA1+ cDC2s CD40+	% BDCA2+ pDCs CD40+	0.78	*0.001*	0.57	*0.516*	0.37	*1*
% BDCA1+ cDC2s CD86+	% BDCA2+ pDCs CD40+	0,66	*0.032*	0.39	*1*	0.39	*1*
	% BDCA3+ cDC1s CD86+	0.70	*0.012*	0.78	*0.008*	0.75	*0.0008*
% BDCA2+ pDCs CD80+	% BDCA2+ pDCs CD86+	0.17	*1*	-0.48	*1*	-0.71	*0.003*
% BDCA2+ pDCs CD80+	% BDCA3+ cDC1s CD80+	0.84	*<0.0001*	0.71	*0.046*	0.76	*0.0006*
% BDCA2+ pDCs CD86+		0.33	*1*	-0.34	*1*	-0.73	*0.002*

Suppl Figure 4

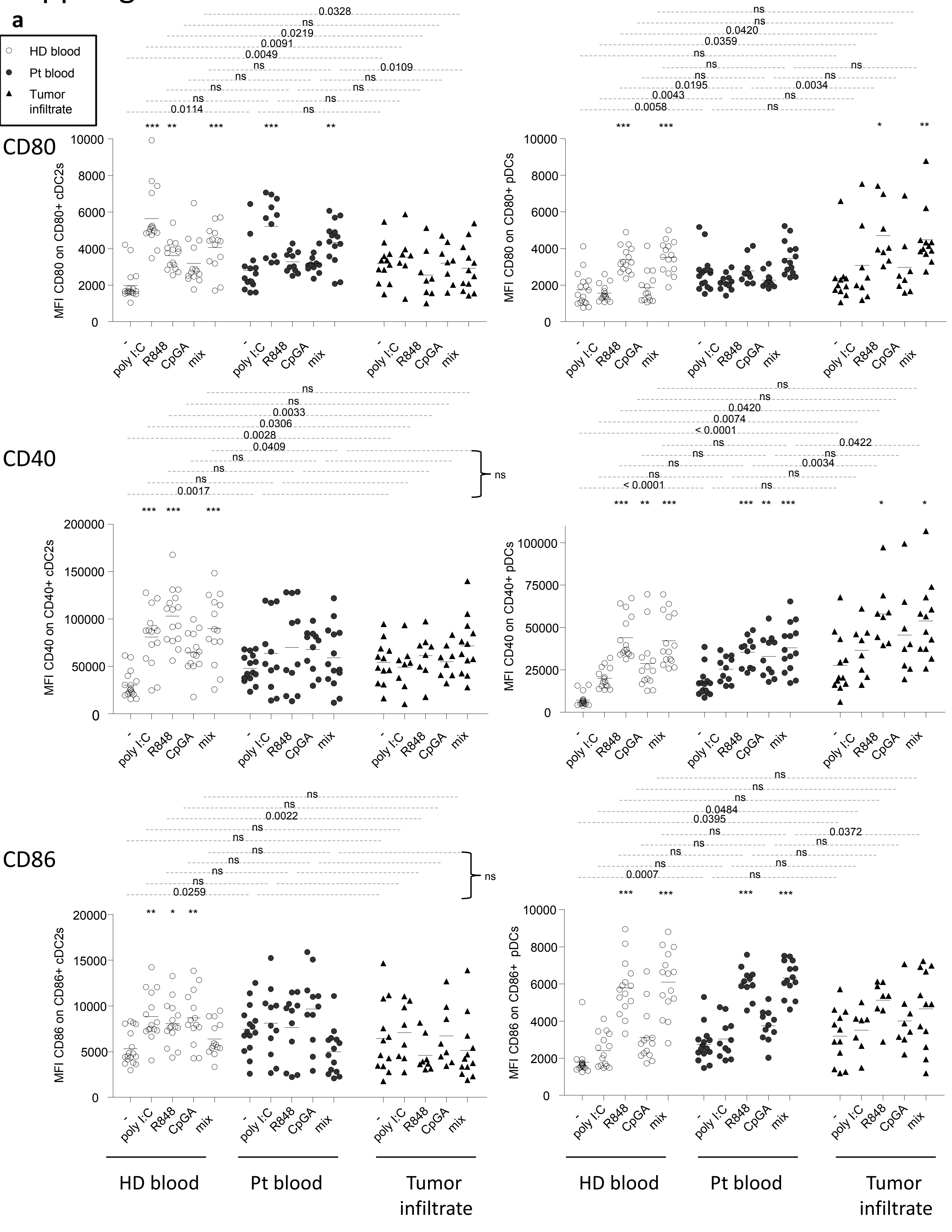

b

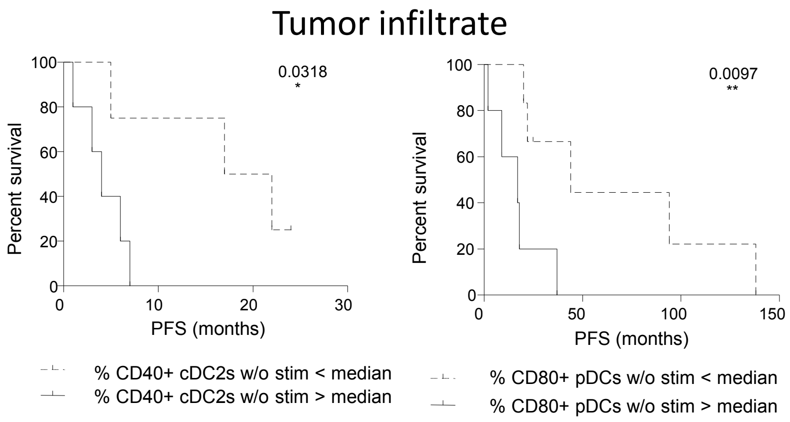
Tumor infiltrate
Percent survival
PFS (months)
0.0318
*
0.0097
**
% CD40+ cDC2s w/o stim < median
% CD40+ cDC2s w/o stim > median
% CD80+ pDCs w/o stim < median
% CD80+ pDCs w/o stim > median

c

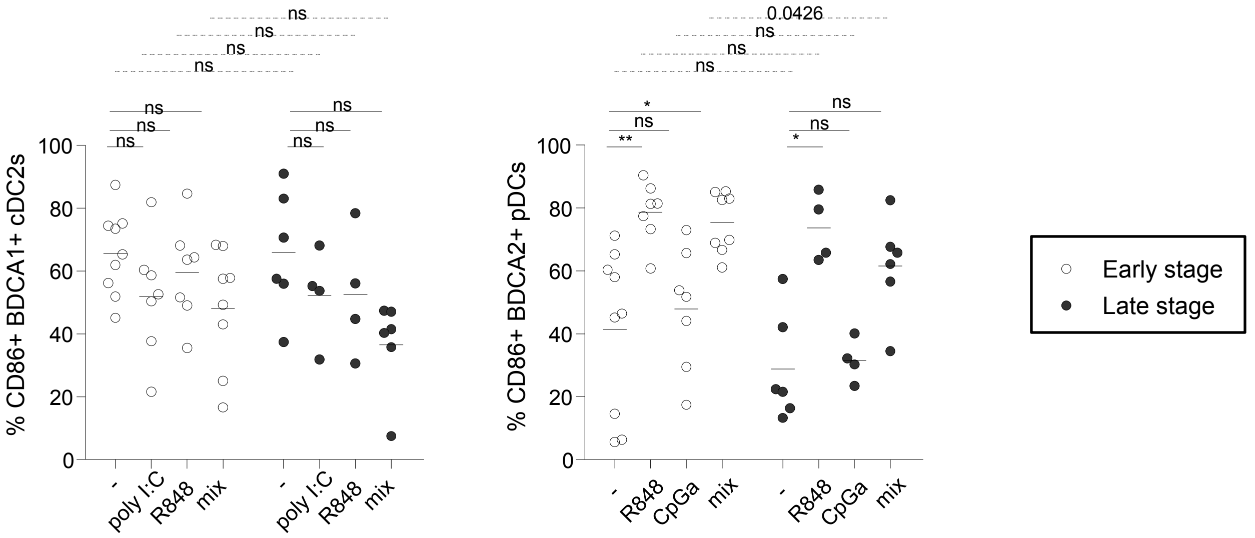
% CD86+ BDCA1+ cDC2s
% CD86+ BDCA2+ pDCs
ns
0.0426
*
**
- poly I:C R848 mix
- R848 CpGa mix
Early stage
Late stage

d

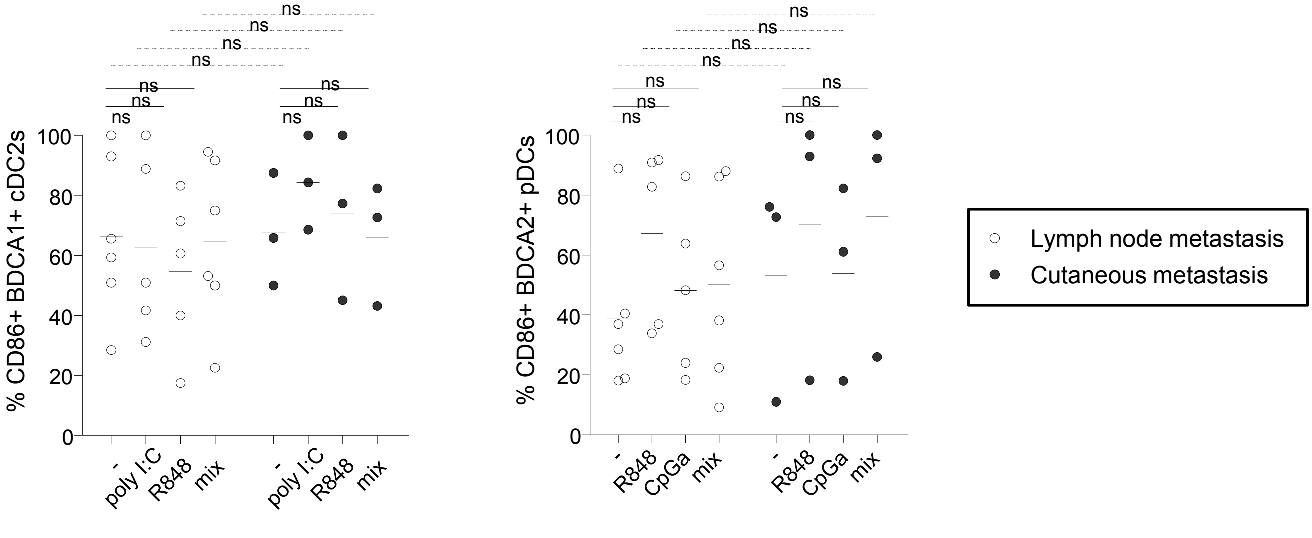
% CD86+ BDCA1+ cDC2s
% CD86+ BDCA2+ pDCs
ns
- poly I:C R848 mix
- R848 CpGa mix
Lymph node metastasis
Cutaneous metastasis

e

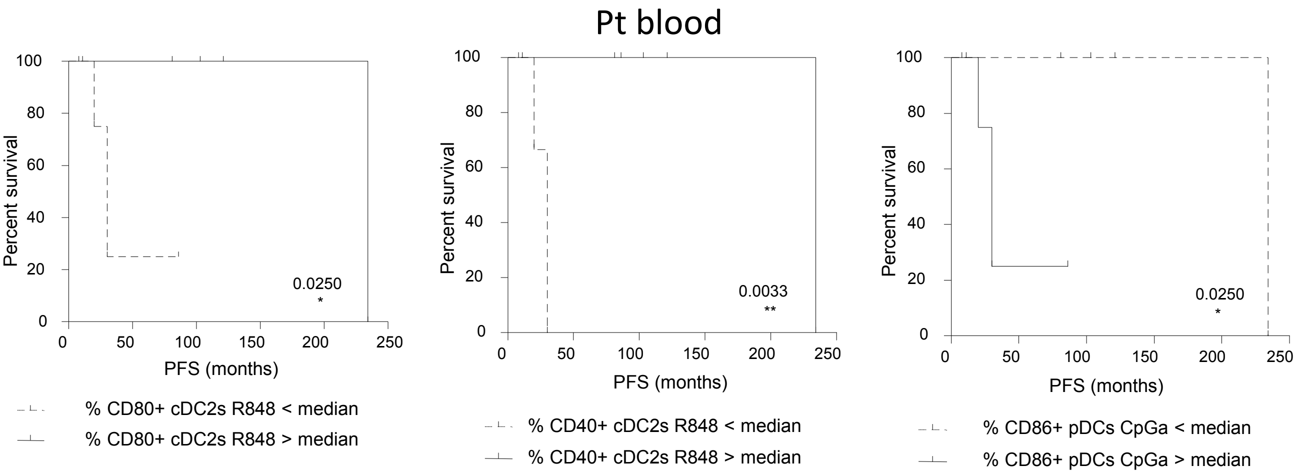
Pt blood
Percent survival
100
80
60
40
20
0
0 50 100 150 200 250
PFS (months)
0.0250
*
% CD80+ cDC2s R848 < median
% CD80+ cDC2s R848 > median
0.0033
**
% CD40+ cDC2s R848 < median
% CD40+ cDC2s R848 > median
0.0250
*
% CD86+ pDCs CpGa < median
% CD86+ pDCs CpGa > median

f

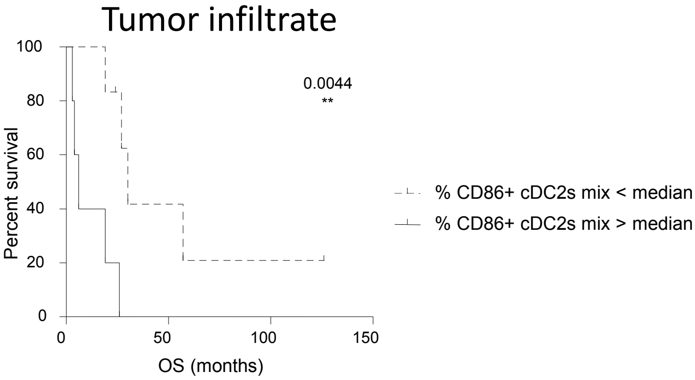
Tumor infiltrate
Percent survival
100
80
60
40
20
0
0 50 100 150
OS (months)
0.0044
**
% CD86+ cDC2s mix < median
% CD86+ cDC2s mix > median

Suppl Figure 5

a

Pt blood

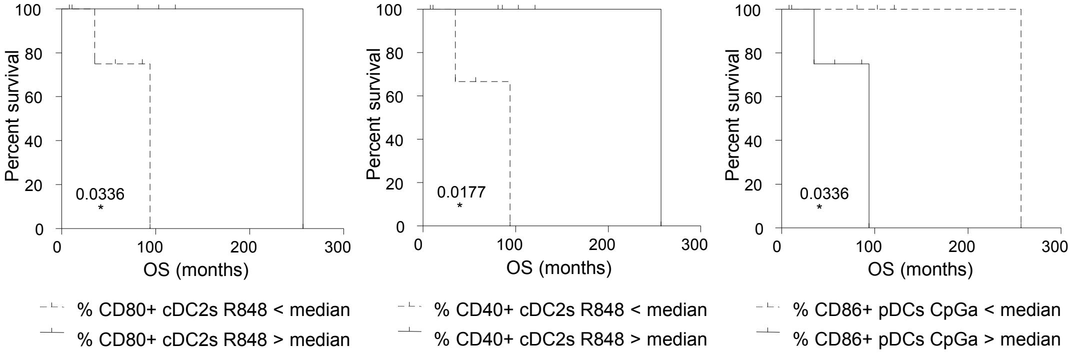

b

Tumor infiltrate

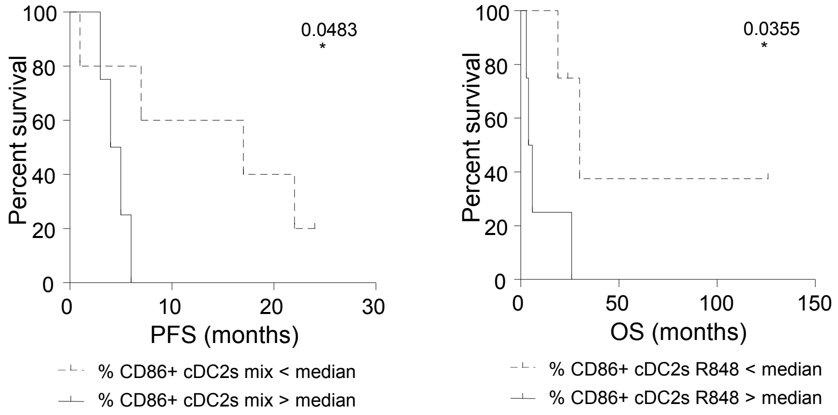

c

Tumor infiltrate

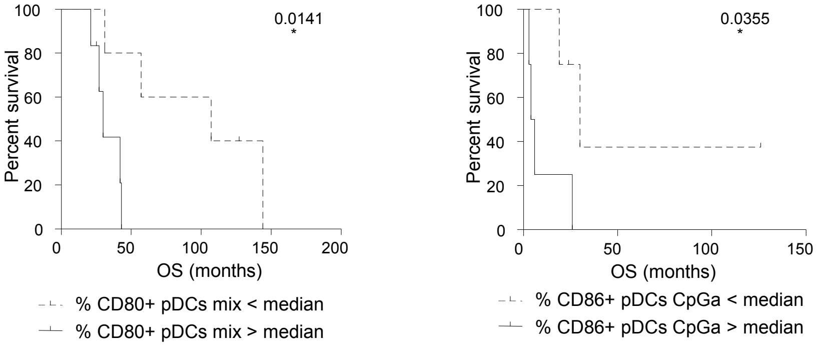

Suppl Figure 6

a

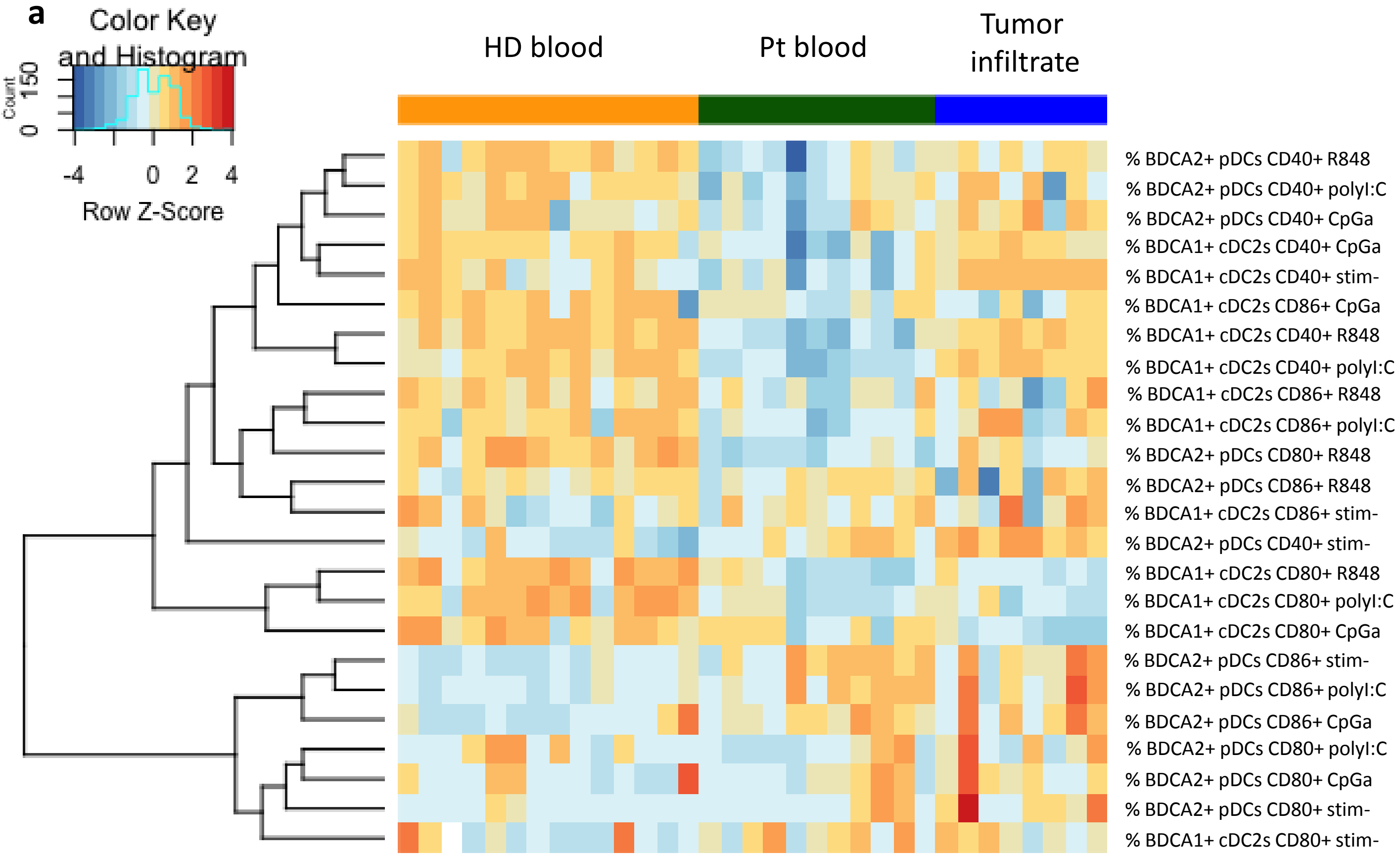

b

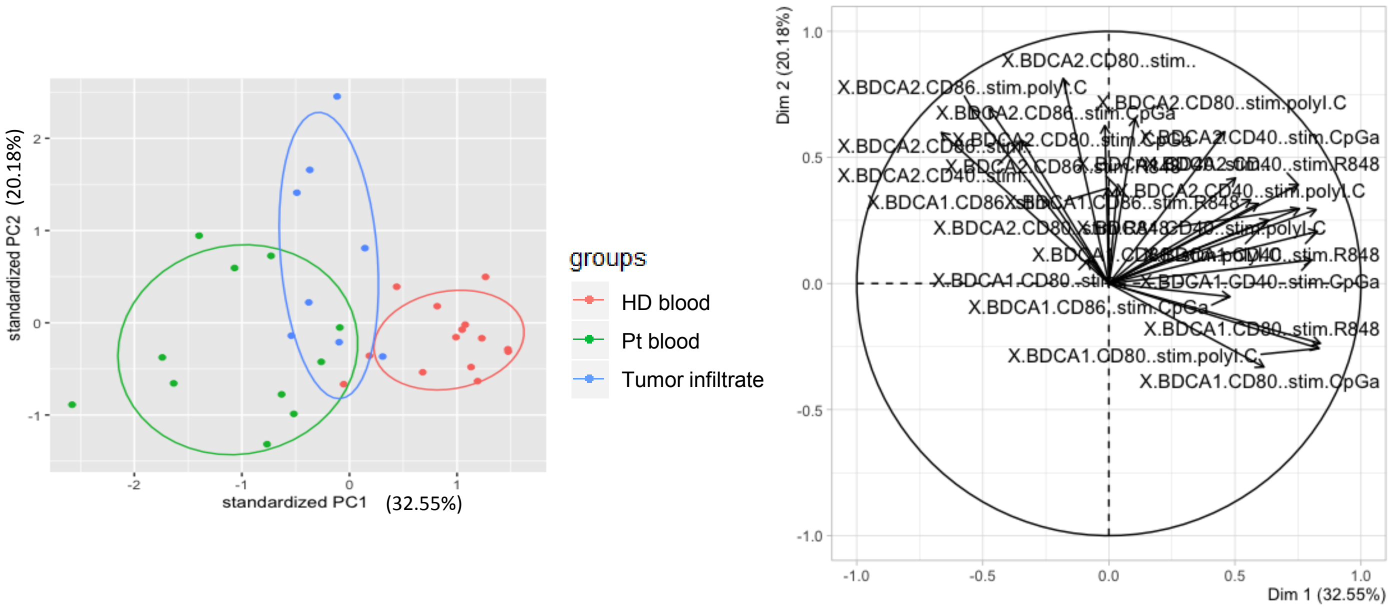

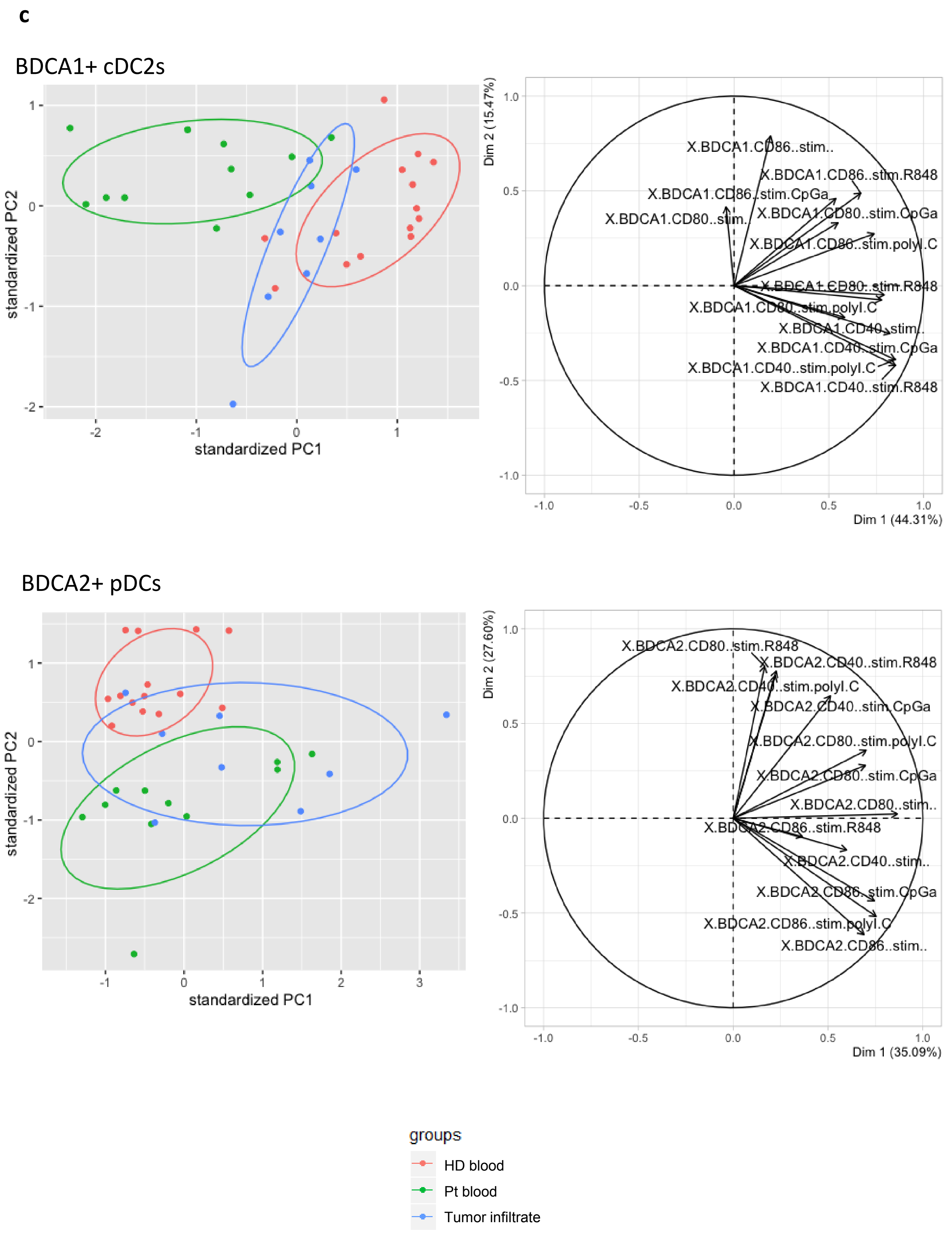
c
BDCA1+ cDC2s
standardized PC2
standardized PC1
Dim 2 (15.47%)
Dim 1 (44.31%)
X.BDCA1.CD86..stim..
X.BDCA1.CD86..stim.R848
X.BDCA1.CD86..stim.CpGa
X.BDCA1.CD80..stim..
X.BDCA1.CD80..stim.CpGa
X.BDCA1.CD86..stim.polyI.C
X.BDCA1.CD80..stim.R848
X.BDCA1.CD80..stim.polyI.C
X.BDCA1.CD40..stim..
X.BDCA1.CD40..stim.CpGa
X.BDCA1.CD40..stim.polyI.C
X.BDCA1.CD40..stim.R848
BDCA2+ pDCs
standardized PC2
standardized PC1
Dim 2 (27.60%)
Dim 1 (35.09%)
X.BDCA2.CD80..stim.R848
X.BDCA2.CD40..stim.R848
X.BDCA2.CD40..stim.polyI.C
X.BDCA2.CD40..stim.CpGa
X.BDCA2.CD80..stim.polyI.C
X.BDCA2.CD80..stim.CpGa
X.BDCA2.CD80..stim..
X.BDCA2.CD86..stim.R848
X.BDCA2.CD40..stim..
X.BDCA2.CD86..stim.CpGa
X.BDCA2.CD86..stim.polyI.C
X.BDCA2.CD86..stim..
groups
HD blood
Pt blood
Tumor infiltrate

d

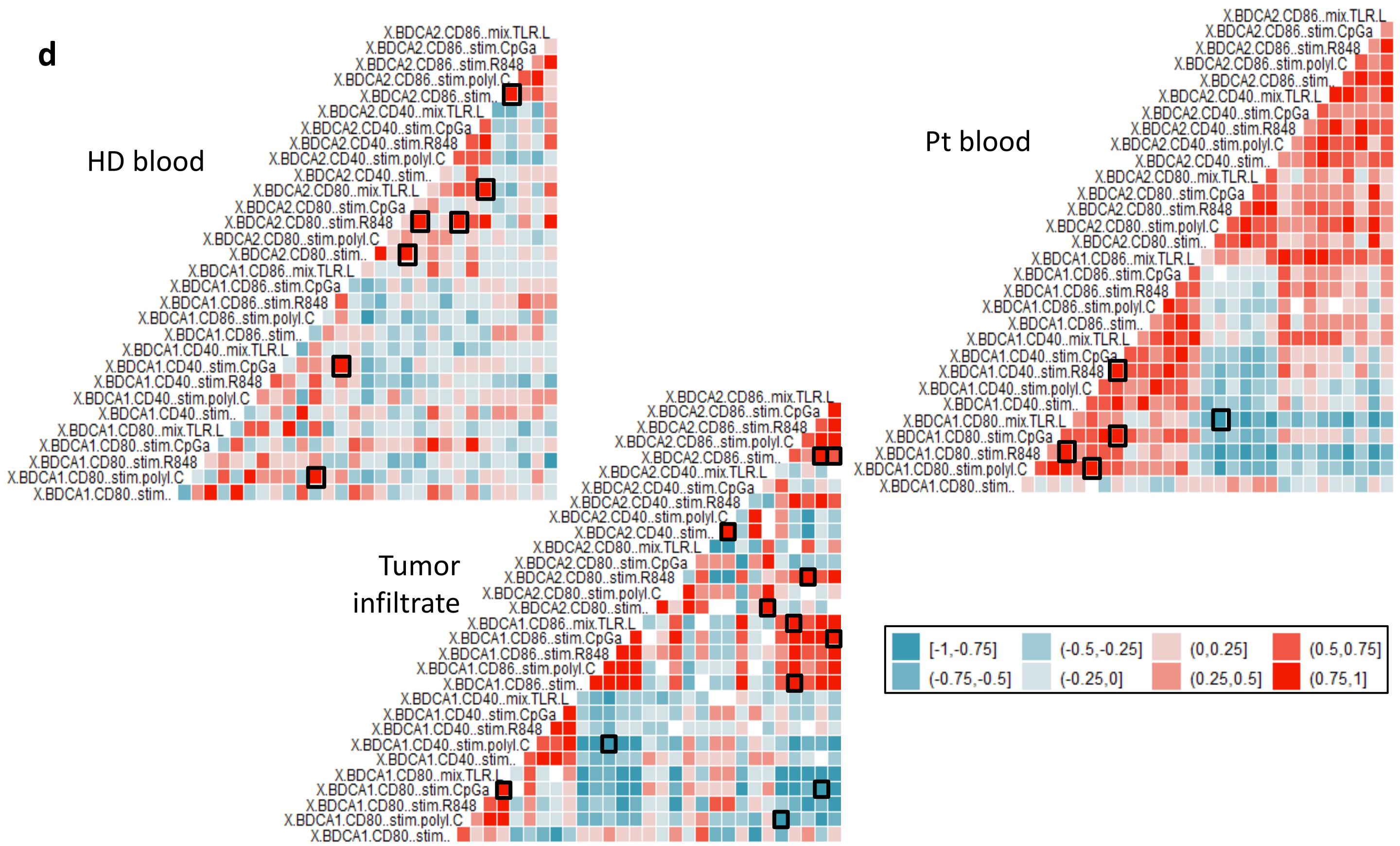

e

w/o stim

polyI:C

R848

CpGa

mix

		Variables		HD blood r	HD blood p	Pt blood r	Pt blood p	Tumor infiltrate r	Tumor infiltrate p
Intra-DCs	cDC2s	CD80+	CD80+	-0.55	1	0.65	1	0.95	0.027
		CD80+		0.61	1	0.96	0.0008	0.90	0.20
		CD80+	CD40+	0.84	0.067	0.90	0.035	-0.09	1
		CD80+	CD40+	0.10	1	0.95	0.002	0.19	1
		CD80+	CD86+	0.89	0.012	0.26	1	-0.20	1
		CD40+	CD40+	0.47	1	0.90	0.035	0.75	1
		CD40+	CD86+	-0.47	1	0.57	1	-1	<0.001
		CD40+	CD86+	0.93	0.0003	-0.42	1		
	pDCs	CD80+	CD80+	0.83	0.038	0.83	0.291	0.59	1
		CD80+	CD80+	0.88	0.006	0.87	0.121	0.81	1
		CD80+	CD40+	-0.04	1	0.27	1	1	<0.0001
		CD80+	CD40+	0.86	0.018	0.28	1	0.56	1
		CD80+	CD40+	0.87	0.010	0.44	1	0.81	1
		CD80+	CD86+	0.38	1	0.10	1	1	<0.0001
		CD40+	CD40+	0.22	1	0.40	1	1	<0.0001
		CD86+	CD86+	0.93	0.0003	0.79	0.586	0.93	0.089
			CD86+	0.72	0.526	0.59	1	0.95	0.027
			CD86+	0.16	1	0.55	1	0.98	0.003
Inter-DCs		cDC2s CD80+	pDCs CD80+	-0.10	1	-0.97	0.021	-0.2	1
		cDC2s CD80+	pDCs CD86+	0.03	1	-0.43	1	-1	<0.0001
		cDC2s CD80+	pDCs CD86+	-0.05	1	0.93	0.23	-1	<0.0001
		cDC2s CD86+	pDCs CD86+	0.43	1	0.30	1	1	<0.0001
		cDC2s CD86+	pDCs CD86+	0.09	1	0.27	1	1	<0.0001
		cDC2s CD86+	pDCs CD86+	-0.04	1	0.60	1	1	<0.0001

Suppl Figure 7

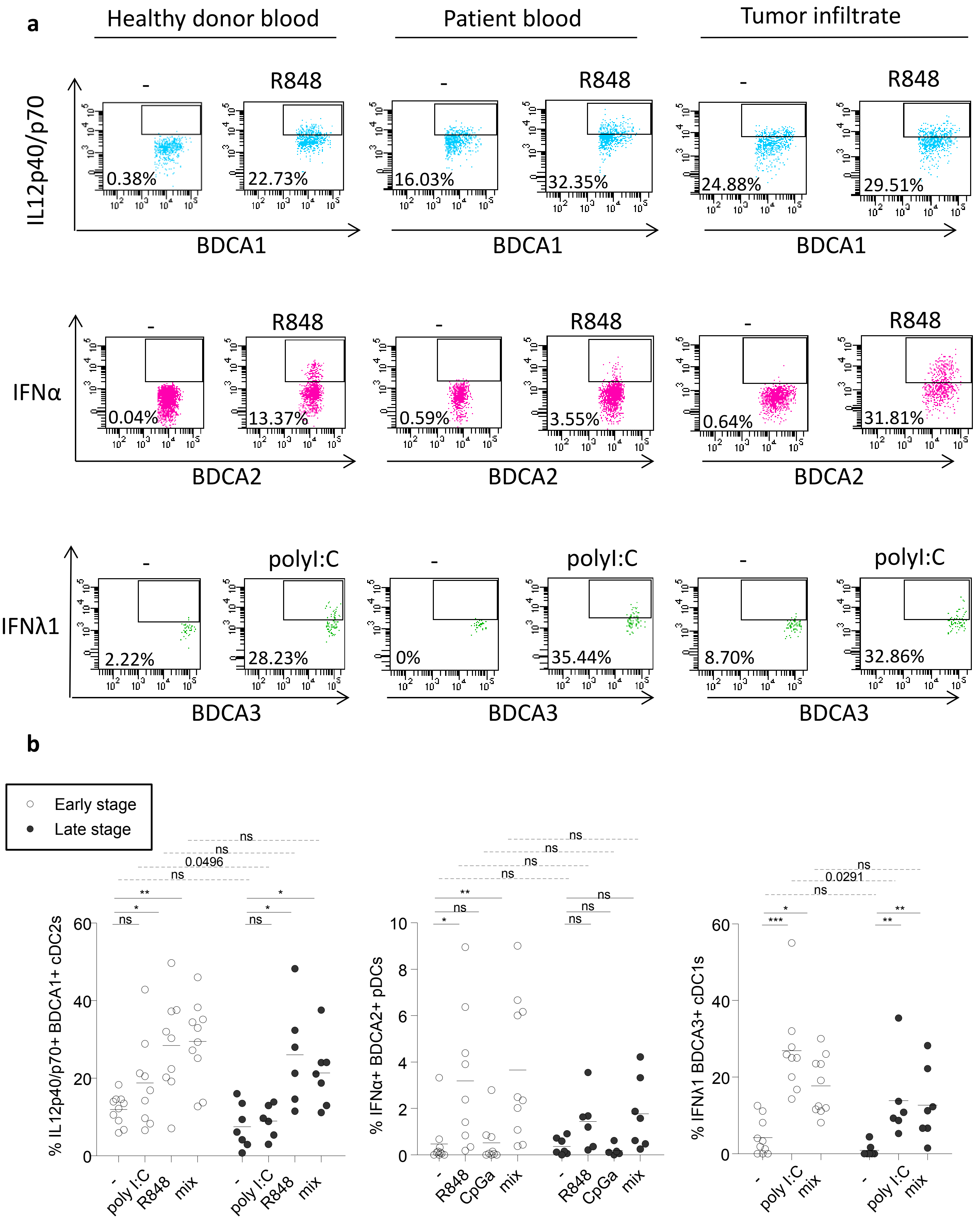

Suppl Figure 8

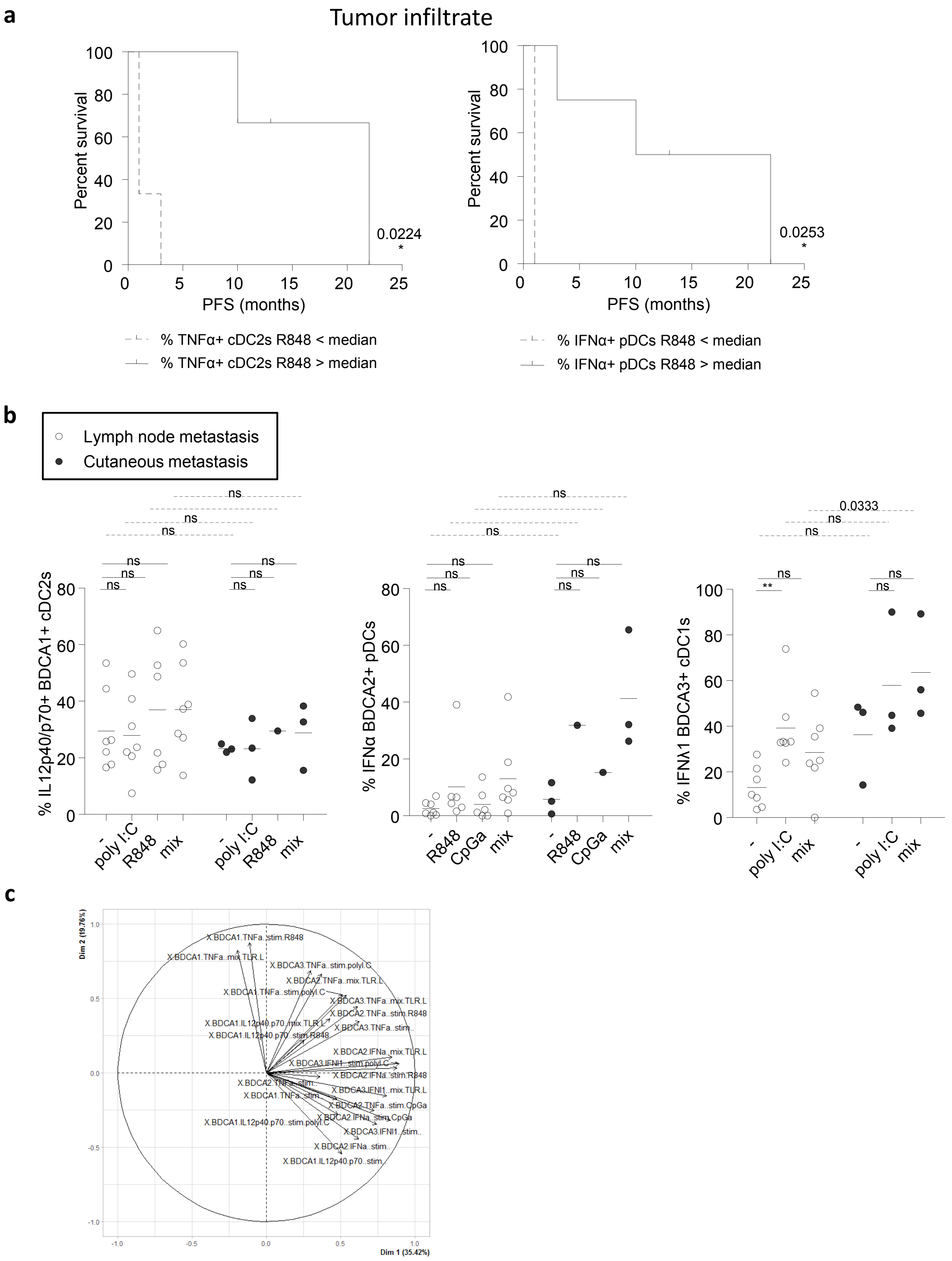

d

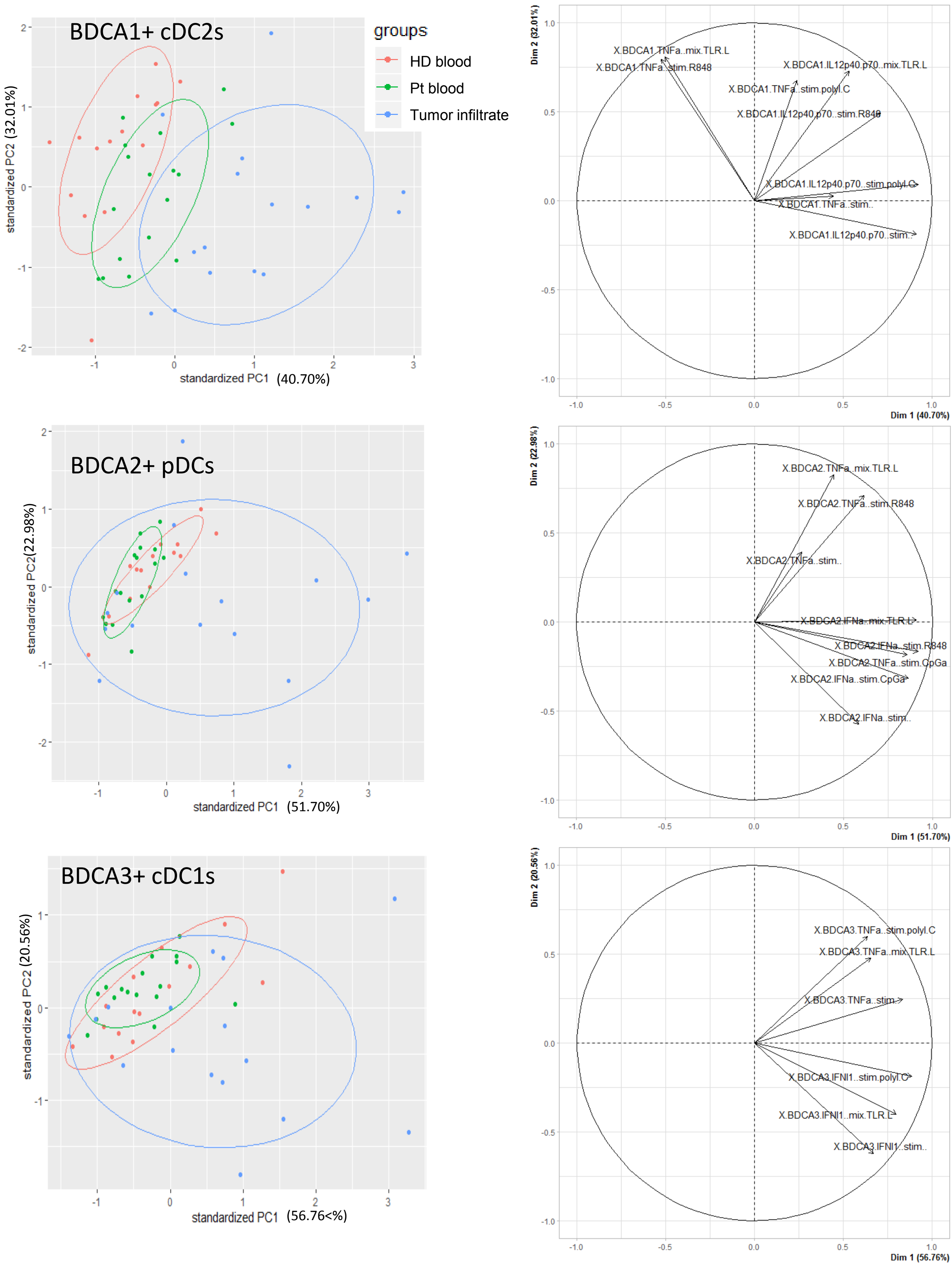

BDCA1+ cDC2s
groups
HD blood
Pt blood
Tumor infiltrate
standardized PC1 (40.70%)
standardized PC2 (32.01%)
X.BDCA1.TNFa..mix.TLR.L
X.BDCA1.TNFa..stim.R848
X.BDCA1.IL12p40.p70..mix.TLR.L
X.BDCA1.TNFa..stim.polyI.C
X.BDCA1.IL12p40.p70..stim.R848
X.BDCA1.IL12p40.p70..stim.polyI.C
X.BDCA1.TNFa..stim..
X.BDCA1.IL12p40.p70..stim..
Dim 1 (40.70%)
Dim 2 (32.01%)
BDCA2+ pDCs
standardized PC1 (51.70%)
standardized PC2(22.98%)
X.BDCA2.TNFa..mix.TLR.L
X.BDCA2.TNFa..stim.R848
X.BDCA2.TNFa..stim..
X.BDCA2.IFNa..mix.TLR.L
X.BDCA2.IFNa..stim.R848
X.BDCA2.TNFa..stim.CpGa
X.BDCA2.IFNa..stim.CpGa
X.BDCA2.IFNa..stim..
Dim 1 (51.70%)
Dim 2 (22.98%)
BDCA3+ cDC1s
standardized PC1 (56.76<%)
standardized PC2 (20.56%)
X.BDCA3.TNFa..stim.polyI.C
X.BDCA3.TNFa..mix.TLR.L
X.BDCA3.TNFa..stim..
X.BDCA3.IFNl1..stim.polyI.C
X.BDCA3.IFNl1..mix.TLR.L
X.BDCA3.IFNl1..stim..
Dim 1 (56.76%)
Dim 2 (20.56%)

e

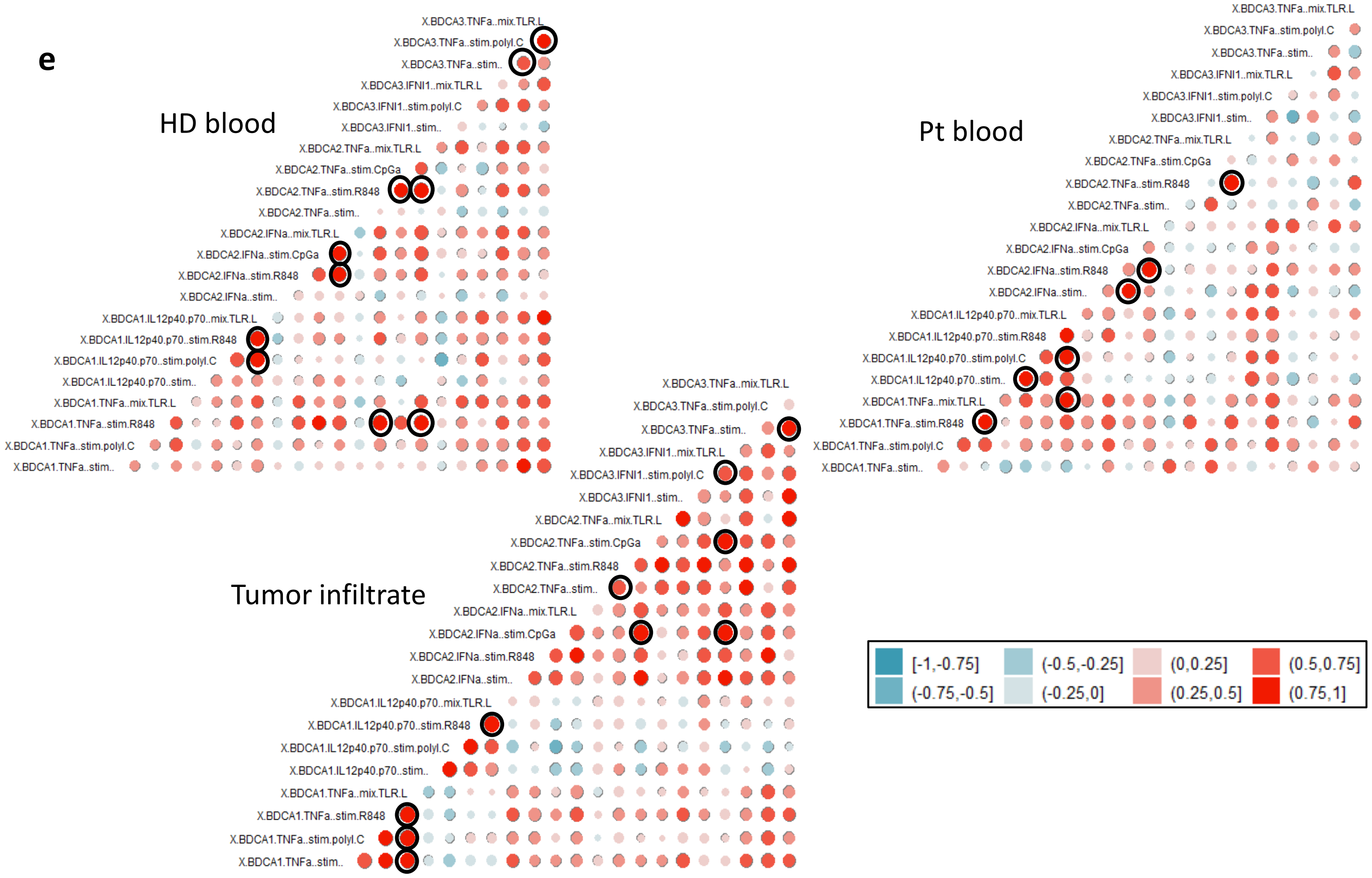

f

- w/o stim
- polyI:C
- R848
- CpGa
- mix

		Variables		HD blood		Pt blood		Tumor infiltrate	
				r	*p*	r	*p*	r	*p*
Intra-DCs	cDC2s	IL-12p40/p70	IL-12p40/p70	0.39	*1*	0.81	*0.007*	0.76	*0.234*
		IL-12p40/p70	IL-12p40/p70	0.88	*0.002*	0.80	*0.009*	0.68	*0.550*
		IL-12p40/p70		0.87	*0.003*	0.72	*0.053*	0.93	*0.003*
		IL-12p40/p70	TNFα	0.54	*1*	0.76	*0.023*	0.006	*1*
		TNFα		0.62	*0.153*	-0.39	*1*	0.86	*0.003*
		TNFα		0.64	*0.450*	0.49	*1*	0.95	*0.0006*
		TNFα	TNFα	0.50	*1*	0.81	*0.006*	0.95	*0.0006*
	pDCs	IFNα	IFNα	0.13	*1*	0.78	*0.023*	0.72	*0.410*
		IFNα	IFNα	0.85	*0.006*	0.87	*0.001*	0.78	*0.196*
		IFNα		0.82	*0.016*	0.39	*1*	0.74	*0.334*
		IFNα	TNFα	0.39	*1*	-0.13	*1*	0.97	*0.0002*
		TNFα	TNFα	-0.04	*1*	0.05	*1*	0.88	*0.020*
		TNFα	TNFα	0.79	*0.036*	0.05	*1*	0.51	*1*
		TNFα	TNFα	0.89	*0.001*	0.89	*0.0004*	0.82	*0.103*
	cDC1s	IFNλ1	IFNλ1	0.31	*1*	0.21	*1*	0.77	*0.032*
		TNFα	TNFα	0.78	*0.008*	0.33	*1*	0.53	*0.568*
		TNFα	TNFα	0.62	*0.153*	-0.39	*1*	0.86	*0.003*
		TNFα		0.89	*0.0001*	0.38	*1*	0.42	*0.695*
Inter-DCs		cDC2s TNFα	pDCs TNFα	0.87	*0.022*	0.55	*1*	0.43	*1*
			pDCs TNFα	0.92	*0.002*	0.61	*1*	0.27	*1*
		pDCs IFNα	cDC1s IFNλ1	0.13	*1*	0.04	*1*	0.92	*0.026*
		pDCs TNFα		-0.46	*1*	0.37	*1*	0.96	*0.002*

Suppl Figure 9

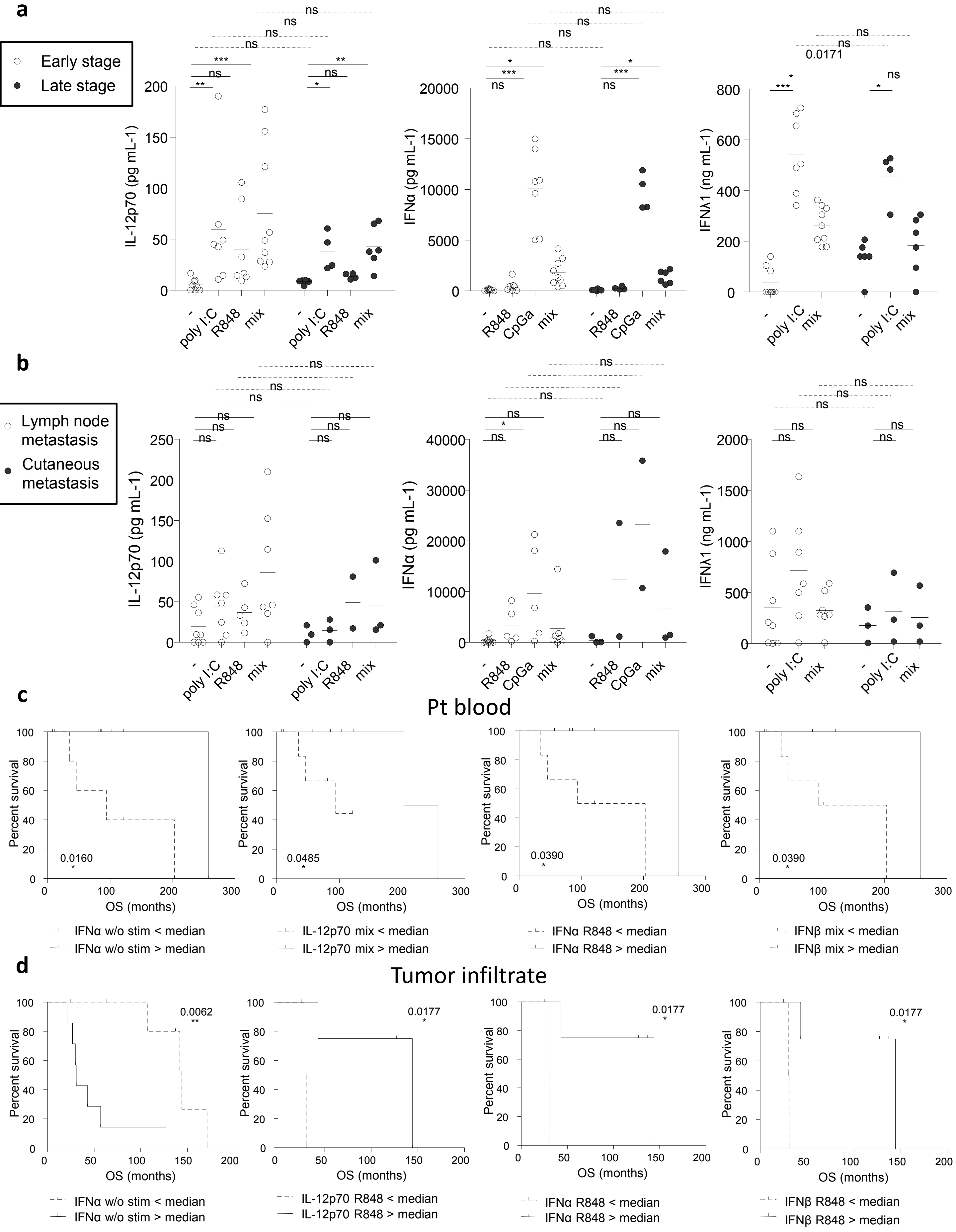

e

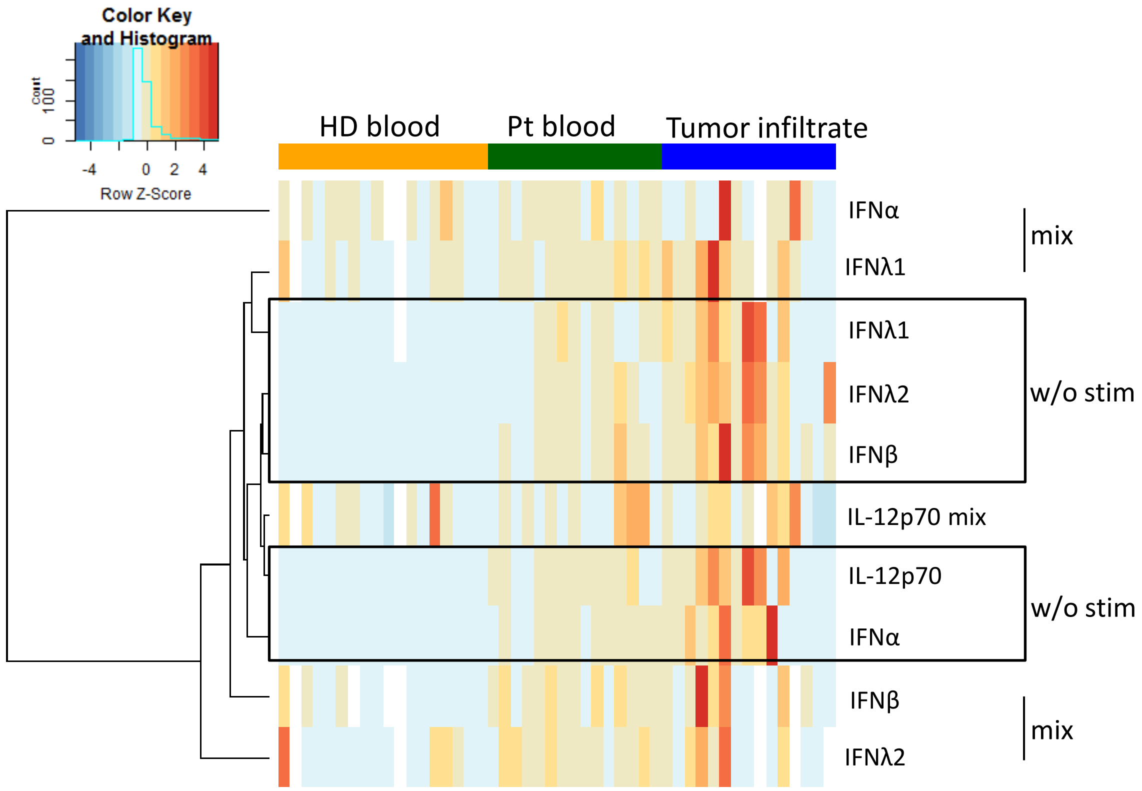

Color Key and Histogram
Count
0
100
-4
0
2
4
Row Z-Score
HD blood
Pt blood
Tumor infiltrate
IFNα
IFNλ1
mix
IFNλ1
IFNλ2
w/o stim
IFNβ
IL-12p70 mix
IL-12p70
w/o stim
IFNα
IFNβ
mix
IFNλ2

Suppl Figure 10

a

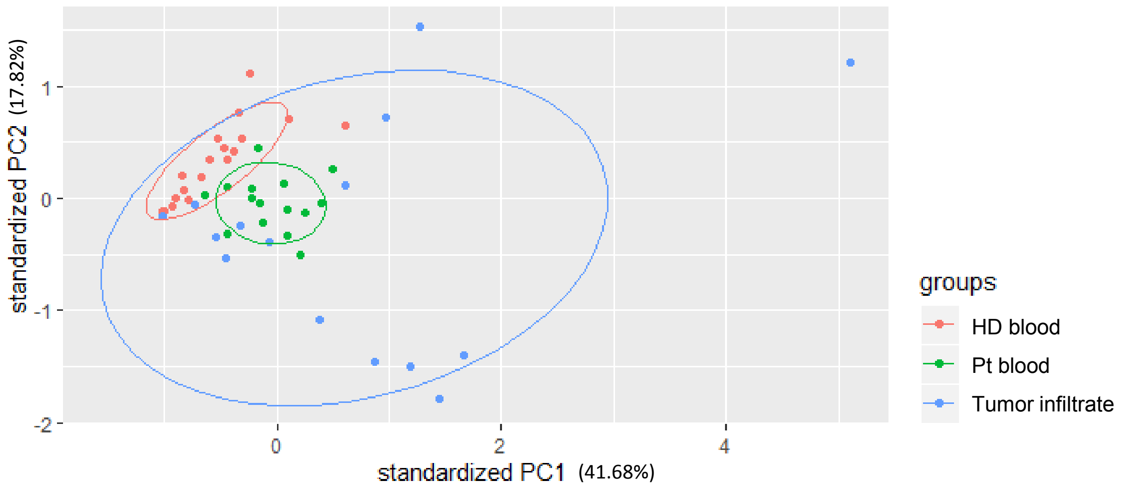

b

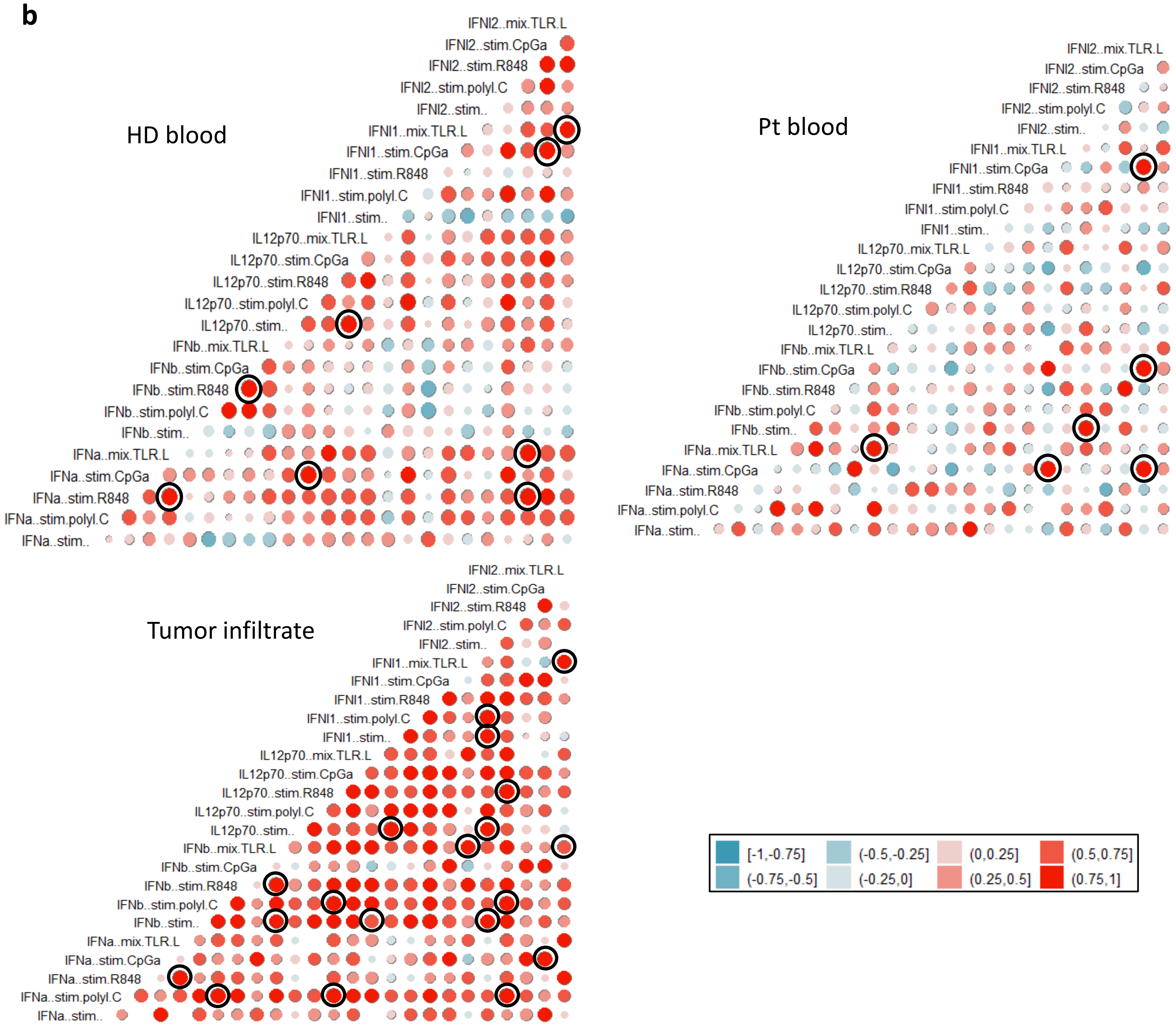

c

- w/o stim
- polyI:C
- R848
- CpGa
- mix

Variables		HD blood		Pt blood		Tumor infiltrate	
		r	*p*	r	*p*	r	*p*
IL-12p70	IL-12p70	0.86	*0.043*	-0.005	*1*	0.70	*1*
IL-12p70	IFNα	0.91	*0.006*	-0.03	*1*	0.30	*1*
IL-12p70	IFNα	0.61	*1*	-0.10	*1*	1	*<0.0001*
IL-12p70	IFNβ	0.09	*1*	0.34	*1*	0.80	*0.042*
IL-12p70	IFNβ	0.066	*1*	-0.15	*1*	1	*<0.0001*
IL-12p70	IFNλ1	0.19	*1*	0.45	*1*	0.97	*<0.0001*
	IFNλ2	0.58	*1*	0.73	*0.088*	0.81	*0.033*
IL-12p70	IFNλ2	0.51	*1*	-0.28	*1*	1	*<0.0001*
IFNα	IFNα	0.87	*0.028*	0.03	*1*	1	*<0.0001*
	IFNβ	0.60	*0.882*	0.92	*<0.0001*	0.44	*1*
IFNα	IFNβ	0.14	*1*	0.86	*0.178*	1	*<0.0001*
IFNα	IFNλ1	0.71	*1*	0.93	*0.012*	0.90	*1*
IFNα	IFNλ2	0.57	*1*	0.74	*1*	1	*<0.0001*
IFNα	IFNλ2	0.67	*1*	0.945	*0.003*	1	*<0.0001*
IFNα	IFNλ2	0.88	*0.022*	0.39	*1*	0.50	*1*
IFNα		0.89	*0.014*	0.14	*1*	0.5	*1*
IFNβ	IFNβ	-0.42	*1*	0.51	*1*	0.80	*0.041*
IFNβ	IFNβ	0.70	*1*	0.40	*1*	1	*<0.0001*
IFNβ		0.88	*0.018*	0.18	*1*	0.10	*1*
IFNβ	IFNλ1	0.39	*1*	0.61	*0.545*	0.88	*0.002*
IFNβ	IFNλ2	0.22	*1*	0.84	*0.003*	0.93	*0.0002*
IFNβ	IFNλ2	0.48	*1*	0.53	*1*	1	*<0.0001*
IFNβ	IFNλ2	0.24	*1*	0.96	*0.0006*	0.90	*1*
IFNβ	IFNλ2	0.43	*1*	0.60	*0.663*	0.90	*0.0015*
IFNλ1	IFNλ2	0.23	*1*	0.61	*0.550*	0.80	*0.037*
IFNλ1		0.26	*1*	0.29	*1*	1	*<0.0001*
IFNλ1	IFNλ2	0.88	*0.022*	0.90	*0.047*	0.90	*1*
IFNλ1	IFNλ2	0.85	*0.006*	0.73	*0.08*	0.81	*0.030*

Suppl Figure 11

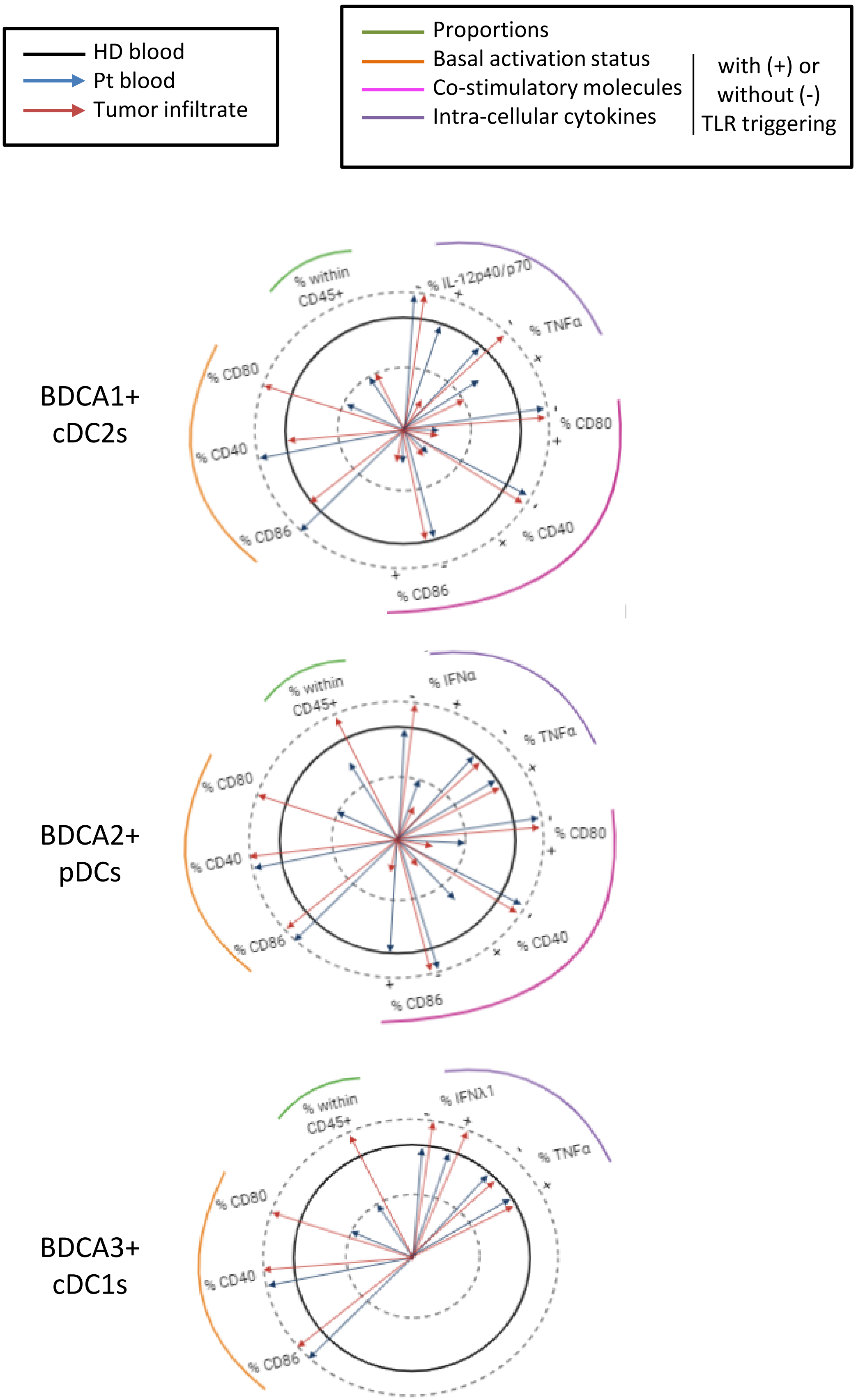

Suppl Figure 12

Blood

		BDCA1+ cDC2s	BDCA2+ pDCs	BDCA3+ cDC1s
Frequency		- -	-	- -
In situ activation		++	++	+/++
After TLR triggering	Activation status	- - -	-	
	Cytokine production	-	- -	+

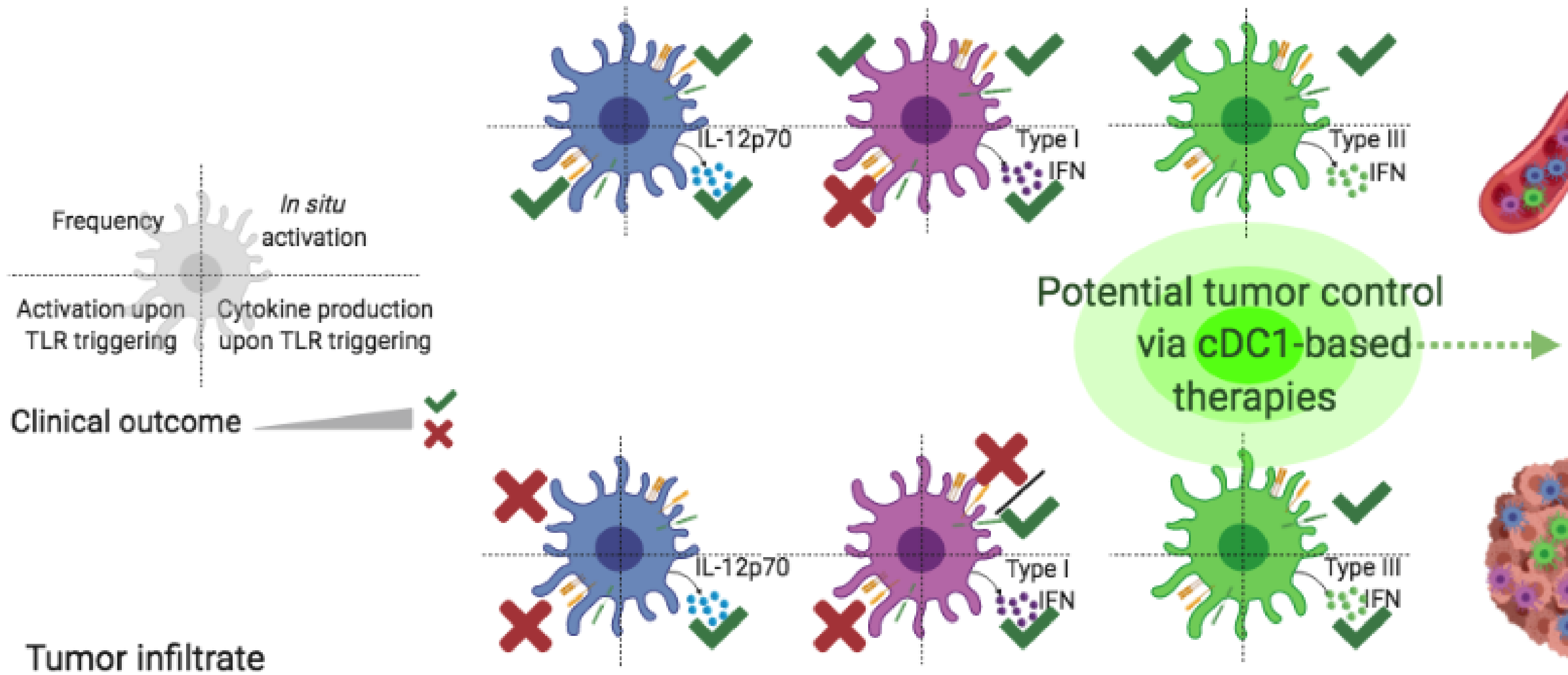

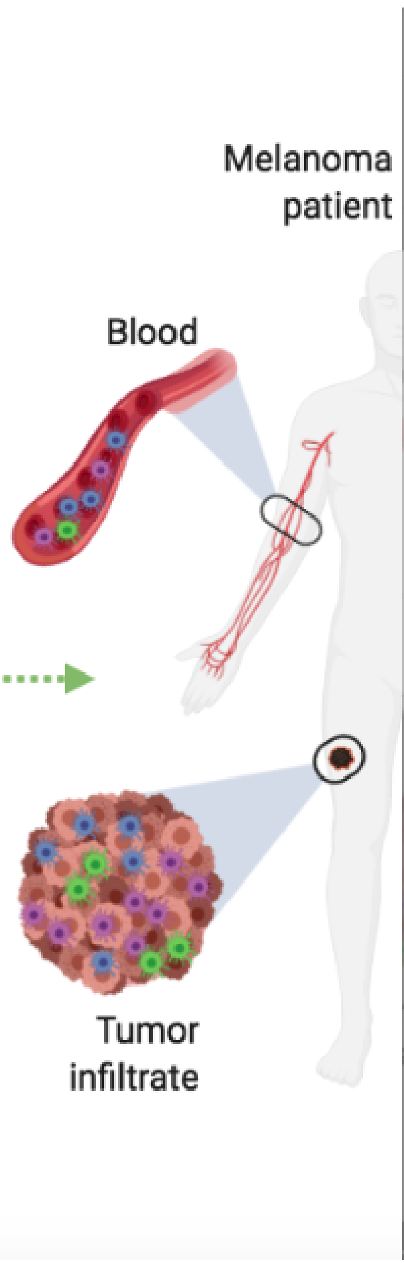

Tumor infiltrate

		BDCA1+ cDC2s	BDCA2+ pDCs	BDCA3+ cDC1s
Frequency		- -	+++	++
In situ activation		+/++	++	++/+++
After TLR triggering	Activation status	- - -	- - -	
	Cytokine production	- - -	- - -	++

Supplementary Table 1: Clinical features of patients from whom blood samples were used

Patient clinical features									From diagnosis time		From sampling time	
#	sample type	sex	age	Breslow (mm)	Clark	Ulcer	treatment before sampling	TNM classification (at sampling time)	PFS	OS	PFS	OS
1	PBMC	F	38	3	IV	yes	no	IIIb (T3b N1a M0)	41	46	41	46
2	PBMC	F	48	1.6	IV	yes	no	IIa (T2b N0 M0)	>122	>122	>122	>122
3	PBMC	M	62	1.2	III	ND	no	Ib ou IIa (T2 N0 MO)	>121	>121	>120	>120
4	PBMC	M	73	0.71 / 0.95	III / ND	no /no	surgery	Ia (T1a N0 M0) or Ib (T1b N0 M0)	>43	203	>75	171
5	PBMC	M	66	1.9	IV	yes		IIa (T2b N0 M0)	>185	>185	>184	>184
6	PBMC	F	41	2.8	III	no	surgery; chemotherapy; IFNα	IV (T3a N1b M1a)	4	23	3	9
7	PBMC	M	48	1.35	III	no	no	IIIa (T2a N2a M0)	>103	>103	>102	>102
8	PBMC	M	89	4 / 2	ND / IV	ND	surgery	IV (T3 Nx M1a)	234	257	11	23
9	PBMC	M	35	1	IV	no	no	IIIa (T1a N1a M0)	>121	>121	>120	>120
10	PBMC	F	42	1.05	IV	no	surgery	IIIb (T2a N2c M0)	22	>85	>85	>85
11	PBMC	F	80	4.1	IV	yes	no	IIc (T4b N0 M0)	20	94	19	93
12	PBMC	F	45	0.54	III	no	no	Ia (T1a N0 M0)	>8	>8	>8	>8
13	PBMC	F	49	0.4 / 1.62	III / III	ND	surgery	IV (T2a N1 M1c)	30	35		5
14	PBMC	F	64	3	IV	ND	no	IIa (T3a N0 M0)	>86	>86	>85	>85
15	PBMC	F	72	1.1	IV	no	no	Ib (T2a N0 M0)	>11	>11	>10	>10
16	PBMC	F	61	2.6	IV	yes	no	IIb (T3b N0 M0)	30	>57	30	>57
17	PBMC	F	45	1.5	IV	no	no	Ib (T2a N0 M0)	>81	>81	>81	>81

ND: not determined

Supplementary Table 2: Clinical features of patients from whom tumor samples were used

Patient clinical features									From diagnosis time		From sampling time	
#	sample type	sex	age	Breslow (mm)	Clark	Ulcer	treatment before sampling	TNM classification (at sampling time)	PFS	OS	PFS	OS
18	primary tumor or sub-cutaneous metastasis	F	80	ND	ND	ND	no	IIIc (TxN3M0)		42		42
19	lymph node metastasis	M	26	2.64	IV	ND	surgery; IFN; radiotherapy	IV (T3aN3M1a)	14	84	12	31
20	ND	F	51	0.8	III	no	no		50			
21	lymph node metastasis	F	69	0.78	III	ND	surgery; chemotherapy; vaccination (DC MEL 201)	IV (T1a Nx M1a)	44	107	6	19
22	lymph node metastasis	M	25	ND	ND	ND	no			31		30
23	primary tumor or lymph node metastasis	M	62	1.4	III	yes	no	IIIc (T2b N1b M0)	17	27	17	27
24	ND	F	72	3.09	IV	yes	no		4			
25	ND	M	39	9	IV	yes	surgery		22	57	22	57
26	ND	F	68	6	V	no	no		94			
27	sub-cutaneous metastasis	F	67	1.3	III	no	surgery	IV (T2a N0 M1a)	37	43	3	3
28	primary tumor	M	66	14	V	ND	no	IIIb or IIIc (T4 N2c M0)	13	23	2	11
29	ND	M	46	1.4	IV	no	no		160	213	36	58
30	primary tumor	F	42	0.9	III	no	no	Ia (T1a N0 M0)	>25	>25	>24	>24
31	lymph node metastasis	M	56	8	IV	ND	IFNα	IV (T1a N3 M1c)	113	117	4	4
32	lymph node metastasis	M	50	1.5	III	no	surgery		20	30		26
33	lymph node metastasis	M	33	6.9	IV	no	surgery	IV (T4a N3 M1c)	62	74	10	10
34	lymph node metastasis	F	61	4.1	IV	yes	no	IIIc (T4b N1b M0)	2	>127	1	>126
35	lymph node metastasis	F	75	1	III	no	surgery	IV (T1a N0 M1a)	84	100	1	16
36	pulmonary metastasis	M	83	2.4	III	ND	surgery	IV (T3a N1 M1b)	98	>114		
37	lymph node metastasis	F	75	5	IV	yes	surgery	IIIc (T4b N3 M0)	9	21	7	19
38	lymph node metastasis	M	59	2.3	IV	no	surgery	IIIc (T3a N3 M0)	138	144	5	6
39	primary tumor	M	64	5	IV	yes	no	IIc (T4b N0 M0)	18	42	4	4
40	lymph node metastasis	F	72	2.5	IV	no	surgery	IIIb (T3 N2b M0)	120	>137	>13	>13
41	cutaneous metastasis	F	70	ND	ND	ND	chemotherapy; radiotherapy	IV (Tx Nx M1a)	>35	>35	>37	>37
42	sub-cutaneous metastasis	F	65	1.9	III	no	surgery; radiotherapy; chemotherapy; IFNα	IV (T2 N0 M1a)	13	142	2	19
43	lymph node metastasis	M	44	1	III	ND	surgery	IIIc (T1 N3 M0)	17	27	3	9
44	lymph node metastasis	F	46	2.1	IV	ND	IFNα	IV (T3 N0 M1a)	50	>63	1	>13
45	primary tumor or lymph node metastasis	F	50	2.8	IV	no	no	IIIa (T3 N1a M0)	36	171	36	171

ND: not determined

Supplementary Table 3A: Impact of the frequencies of circulating or tumor-infiltrating DCs on clinical outcome (Log-rank test analysis and Cox regression analysis).

Log-rank (*p*-values)	Patient blood				Patient tumor infiltrate			
	PFS diagnosis	OS diagnosis	PFS sampling	OS sampling	PFS diagnosis	OS diagnosis	PFS sampling	OS sampling
BDCA1 within CD45	0.286	0.943	0.808	0.457	0.241	0.842	0.037	0.062
BDCA2 within CD45	0.028	0.017	0.052	0.085	0.376	0.724	0.173	0.783
BDCA3 within CD45	0.193	0.564	0.005	0.101	0.541	0.209	0.542	0.530

COX regression (HR, *p*-values)	Patient blood								Patient tumor infiltrate							
	PFS diagnosis		OS diagnosis		PFS sampling		OS sampling		PFS diagnosis		OS diagnosis		PFS sampling		OS sampling	
	HR	*p*-value	HR	*p*-value	HR	*p*-value	HR	*p*-value	HR	*p*-value	HR	*p*-value	HR	*p*-value	HR	*p*-value
BDCA1 within CD45	23946.770	0.041	6.056	0.668	14.300	0.494	0.246	0.704	2.801	0.069	2.963	0.116	2.368	0.110	1.596	0.370
BDCA2 within CD45	5.72E-06	0.064	5.41E-06	0.125	0.001	0.294	0.005	0.388	0.679	0.528	0.942	0.931	1.230	0.796	1.791	0.408
BDCA3 within CD45	6.49E-12	0.621	1.55E-55	0.180	2.63E-70	0.071	8.42E-66	0.073	2.521	0.466	2.169	0.563	0.870	0.906	0.618	0.690

Red when *p*-value<0.05

Supplementary Table 3B: Impact of the basal phenotype of circulating or tumor-infiltrating DCs on clinical outcome (Log-rank test analysis and Cox regression analysis).

Log-rank (*p*-values)	Patient blood				Patient tumor infiltrate			
	PFS diagnosis	OS diagnosis	PFS sampling	OS sampling	PFS diagnosis	OS diagnosis	PFS sampling	OS sampling
BDCA1 CD80	0.518	0.794	0.776	0.187	0.015	0.207	0.174	0.551
BDCA1 CD40	0.861	0.810	0.253	0.481	0.385	0.934	0.618	0.639
BDCA1 CD86	0.646	0.435	0.817	0.779	0.774	0.099	0.392	0.735
BDCA2 CD80	0.668	0.711	0.321	0.435	0.257	0.419	0.048	0.096
BDCA2 CD40	0.159	0.564	0.092	0.329	0.151	0.001	0.804	0.235
BDCA2 CD86	0.423	0.640	0.585	0.576	0.033	0.056	0.108	0.212
BDCA3 CD80	0.620	0.674	0.928	0.620	0.111	0.130	0.121	0.224
BDCA3 CD40	0.490	0.165	0.037	0.045	0.135	0.008	0.688	0.635
BDCA3 CD86	0.748	0.170	0.601	0.724	0.947	0.076	0.483	0.790

COX regression (HR, *p*-values)	Patient blood								Patient tumor infiltrate							
	PFS diagnosis		OS diagnosis		PFS sampling		OS sampling		PFS diagnosis		OS diagnosis		PFS sampling		OS sampling	
	HR	*p*-value	HR	*p*-value	HR	*p*-value	HR	*p*-value	HR	*p*-value	HR	*p*-value	HR	*p*-value	HR	*p*-value
BDCA1 CD80	0.938	0.598	1.007	0.955	0.971	0.800	0.993	0.947	1.020	0.040	1.019	0.109	1.010	0.313	0.998	0.849
BDCA1 CD40	0.940	0.171	0.924	0.150	0.799	0.025	0.863	0.026	1.066	0.211	0.975	0.615	1.024	0.625	0.975	0.527
BDCA1 CD86	1.006	0.775	0.981	0.369	1.006	0.791	1.000	0.99	0.985	0.413	0.959	0.026	1.006	0.740	1.004	0.818
BDCA2 CD80	0.956	0.839	1.068	0.769	1.013	0.951	1.037	0.863	1.020	0.226	1.022	0.159	1.016	0.328	1.016	0.267
BDCA2 CD40	0.934	0.045	0.927	0.068	0.924	0.046	0.962	0.195	0.992	0.522	0.960	0.005	1.010	0.533	0.994	0.607
BDCA2 CD86	1.010	0.484	0.995	0.707	1.007	0.640	1.008	0.602	0.987	0.122	0.981	0.042	0.997	0.745	1.000	0.967
BDCA3 CD80	0.897	0.397	0.952	0.703	0.992	0.941	1.014	0.884	1.015	0.101	1.022	0.033	1.013	0.183	1.010	0.304
BDCA3 CD40	0.884	0.111	0.849	0.064	0.851	0.038	0.864	0.055	0.989	0.808	0.922	0.090	1.049	0.399	0.958	0.428
BDCA3 CD86	1.000	0.993	0.958	0.407	1.022	0.718	1.033	0.562	0.988	0.364	0.969	0.034	1.000	0.995	1.005	0.733

Red when *p*-value<0.05

Supplementary Table 4: Impact of the activation status after TLR stimulation of circulating or tumor-infiltrating DCs on clinical outcome (Log-rank test analysis).

Log-rank (*p*-values)	Patient blood				Patient tumor infiltrate			
	PFS diagnosis	OS diagnosis	PFS sampling	OS sampling	PFS diagnosis	OS diagnosis	PFS sampling	OS sampling
BDCA1 CD80 w/o stim	0.963	0.974	0.270	0.231	0.953	0.522	0.084	0.797
BDCA1 CD80 polyIC	0.114	0.018	0.683	0.185	0.767	0.264	0.197	0.909
BDCA1 CD80 R848	0.025	0.034	0.303	0.261	0.472	0.343	0.441	0.872
BDCA1 CD80 CpGa	0.003	0.018	0.075	0.185	0.472	0.343	0.441	0.872
BDCA1 CD80 mix	0.011	0.179	0.487	0.568	0.606	0.121	0.062	0.686
BDCA1 CD40 w/o stim	0.318	0.521	0.538	0.204	0.082	0.845	0.032	0.476
BDCA1 CD40 polyIC	0.317	0.098	0.870	0.433	0.169	0.694	0.343	0.872
BDCA1 CD40 R848	0.003	0.018	0.075	0.185	0.634	0.607	0.694	0.673
BDCA1 CD40 CpGa	0.003	0.018	0.075	0.185	0.300	0.707	0.277	0.142
BDCA1 CD40 mix	0.058	0.295	0.819	0.956	0.921	0.535	0.953	0.679
BDCA1 CD86 w/o stim	0.773	0.600	0.524	0.798	0.625	0.814	0.953	0.527
BDCA1 CD86 polyIC	0.499	0.577	0.722	0.923	0.212	0.832	0.343	0.527
BDCA1 CD86 R848	0.499	0.824	0.722	0.700	0.383	0.863	0.277	0.035
BDCA1 CD86 CpGa	0.114	0.062	0.683	0.337	0.383	0.863	0.277	0.035
BDCA1 CD86 mix	0.617	0.464	0.421	0.963	0.091	0.643	0.048	0.004
BDCA2 CD80 w/o stim	0.530	0.796	0.839	0.354	0.010	0.328	0.132	0.996
BDCA2 CD80 polyIC	0.499	0.577	0.722	0.835	0.309	0.707	0.025	0.762
BDCA2 CD80 R848	0.809	0.949	0.383	0.528	0.780	0.486	0.694	0.355
BDCA2 CD80 CpGa	0.499	0.577	0.722	0.835	0.767	0.343	0.713	0.679
BDCA2 CD80 mix	0.492	0.964	0.311	0.289	0.163	0.014	0.575	0.142
BDCA2 CD40 w/o stim	0.645	0.316	0.853	0.789	0.875	0.625	0.143	0.822
BDCA2 CD40 polyIC	0.809	0.702	0.383	0.721	0.139	0.015	0.951	0.583
BDCA2 CD40 R848	0.589	0.577	0.994	0.120	0.534	0.694	0.832	0.094
BDCA2 CD40 CpGa	0.317	0.171	0.123	0.051	0.863	0.013	0.049	0.544
BDCA2 CD40 mix	0.814	0.271	0.992	0.717	0.533	0.597	0.609	0.985
BDCA2 CD86 w/o stim	0.414	0.316	0.685	0.789	0.302	0.824	0.449	0.188
BDCA2 CD86 polyIC	0.079	0.051	0.619	0.395	0.383	0.863	0.277	0.035
BDCA2 CD86 R848	0.589	0.134	0.508	0.599	0.290	0.109	0.607	0.065
BDCA2 CD86 CpGa	0.025	0.034	0.303	0.311	0.383	0.863	0.277	0.035
BDCA2 CD86 mix	0.537	0.401	0.819	0.884	0.914	0.720	0.625	0.208

Red when *p*-value<0.05; w/o stim: without stimulation

Supplementary Table 5: Impact of the activation status of circulating DCs after TLR stimulation on clinical outcome (Cox regression analysis).

COX regression (HR, *p*-values)	Patient blood							
	PFS diagnosis		OS diagnosis		PFS sampling		OS sampling	
	HR	*p*-value	HR	*p*-value	HR	*p*-value	HR	*p*-value
BDCA1 CD80 w/o stim	0.984	0.539	0.993	0.819	1.009	0.692	1.000	0.985
BDCA1 CD80 polyIC	0.864	0.111	0.898	0.181	0.975	0.485	0.975	0.438
BDCA1 CD80 R848	0.760	0.271	0.910	0.227	0.975	0.437	0.980	0.493
BDCA1 CD80 CpGa	0.901	0.029	0.941	0.098	0.960	0.151	0.970	0.219
BDCA1 CD80 mix	0.911	0.064	0.954	0.264	0.987	0.718	0.997	0.944
BDCA1 CD40 w/o stim	0.946	0.080	0.980	0.483	0.987	0.596	0.987	0.578
BDCA1 CD40 polyIC	0.876	0.031	0.905	0.113	0.957	0.287	0.959	0.253
BDCA1 CD40 R848	0.553	0.745	0.915	0.154	0.946	0.106	0.960	0.191
BDCA1 CD40 CpGa	0.905	0.026	0.933	0.103	0.955	0.121	0.963	0.152
BDCA1 CD40 mix	0.941	0.097	0.992	0.793	0.992	0.743	0.997	0.899
BDCA1 CD86 w/o stim	1.000	0.991	1.041	0.349	1.027	0.420	1.016	0.632
BDCA1 CD86 polyIC	0.967	0.445	0.940	0.327	0.991	0.844	0.968	0.465
BDCA1 CD86 R848	0.954	0.250	0.929	0.268	0.965	0.456	0.945	0.275
BDCA1 CD86 CpGa	0.936	0.133	0.062	0.999	0.902	0.299	0.830	0.125
BDCA1 CD86 mix	0.984	0.557	1.010	0.782	1.012	0.670	1.008	0.806
BDCA2 CD80 w/o stim	1.024	0.632	0.928	0.634	1.036	0.492	0.824	0.519
BDCA2 CD80 polyIC	1.024	0.285	0.987	0.842	1.012	0.619	0.950	0.554
BDCA2 CD80 R848	1.077	0.263	1.093	0.426	1.014	0.818	1.005	0.940
BDCA2 CD80 CpGa	1.024	0.502	0.971	0.715	1.010	0.763	0.971	0.599
BDCA2 CD80 mix	1.003	0.946	1.005	0.923	1.001	0.981	0.969	0.518
BDCA2 CD40 w/o stim	0.989	0.715	1.003	0.942	0.996	0.904	0.991	0.774
BDCA2 CD40 polyIC	0.943	0.414	0.949	0.569	0.912	0.188	0.932	0.342
BDCA2 CD40 R848	0.893	0.127	0.949	0.339	0.943	0.234	0.974	0.573
BDCA2 CD40 CpGa	0.916	0.145	0.935	0.174	0.953	0.229	0.953	0.206
BDCA2 CD40 mix	0.923	0.072	0.994	0.912	0.982	0.641	0.983	0.692
BDCA2 CD86 w/o stim	1.020	0.331	1.026	0.283	1.017	0.446	1.018	0.408
BDCA2 CD86 polyIC	1.263	0.253	1.087	0.202	1.021	0.531	1.019	0.526
BDCA2 CD86 R848	1.042	0.482	1.236	0.268	0.980	0.712	1.037	0.570
BDCA2 CD86 CpGa	1.032	0.281	1.047	0.310	1.019	0.518	1.016	0.653
BDCA2 CD86 mix	0.989	0.761	1.105	0.136	1.011	0.775	1.030	0.517

Red when *p*-value<0.05; w/o stim: without stimulation

Supplementary Table 6: Impact of the activation status of tumor-infiltrating DCs after TLR stimulation on clinical outcome (Cox regression analysis).

COX regression (HR. *p*-values)	Patient tumor infiltrate							
	PFS diagnosis		OS diagnosis		PFS sampling		OS sampling	
	HR	*p*-value	HR	*p*-value	HR	*p*-value	HR	*p*-value
BDCA1 CD80 w/o stim	1.022	0.253	1.046	0.043	0.964	0.194	1.001	0.949
BDCA1 CD80 polyIC	0.995	0.826	1.009	0.775	0.964	0.248	0.981	0.431
BDCA1 CD80 R848	0.955	0.360	1.005	0.890	0.988	0.824	0.969	0.401
BDCA1 CD80 CpGa	1.001	0.983	1.041	0.291	0.969	0.519	0.966	0.308
BDCA1 CD80 mix	1.031	0.174	1.033	0.188	0.972	0.236	0.970	0.220
BDCA1 CD40 w/o stim	1.106	0.227	0.937	0.388	1.290	0.082	1.005	0.922
BDCA1 CD40 polyIC	1.064	0.381	1.062	0.471	1.090	0.434	0.948	0.538
BDCA1 CD40 R848	1.077	0.459	0.979	0.705	1.031	0.695	1.009	0.829
BDCA1 CD40 CpGa	1.072	0.512	1.007	0.943	0.934	0.599	0.963	0.557
BDCA1 CD40 mix	0.982	0.583	0.977	0.434	0.996	0.880	0.980	0.523
BDCA1 CD86 w/o stim	0.983	0.367	1.017	0.324	1.007	0.775	1.035	0.070
BDCA1 CD86 polyIC	0.944	0.066	1.003	0.874	0.982	0.310	1.015	0.373
BDCA1 CD86 R848	0.971	0.241	1.006	0.722	1.010	0.643	1.059	0.055
BDCA1 CD86 CpGa	0.985	0.584	1.018	0.476	1.010	0.739	1.056	0.081
BDCA1 CD86 mix	0.964	0.093	1.006	0.700	1.020	0.438	1.049	0.034
BDCA2 CD80 w/o stim	1.012	0.245	1.012	0.332	1.012	0.269	1.013	0.270
BDCA2 CD80 polyIC	1.003	0.816	0.984	0.421	1.048	0.086	1.019	0.361
BDCA2 CD80 R848	0.983	0.588	1.021	0.403	0.991	0.828	1.054	0.115
BDCA2 CD80 CpGa	1.002	0.893	0.994	0.767	1.024	0.336	1.017	0.587
BDCA2 CD80 mix	1.018	0.213	1.027	0.088	1.001	0.960	1.033	0.081
BDCA2 CD40 w/o stim	1.004	0.699	0.996	0.768	1.019	0.283	1.004	0.718
BDCA2 CD40 polyIC	0.931	0.138	0.785	0.088	1.009	0.795	0.972	0.457
BDCA2 CD40 R848	0.904	0.450	1.096	0.505	1.061	0.661	2.076	0.078
BDCA2 CD40 CpGa	0.967	0.512	0.765	0.079	1.074	0.257	0.983	0.654
BDCA2 CD40 mix	1.006	0.618	1.003	0.820	1.009	0.520	1.011	0.457
BDCA2 CD86 w/o stim	0.986	0.262	0.984	0.273	1.022	0.133	1.026	0.123
BDCA2 CD86 polyIC	0.986	0.332	0.992	0.545	1.015	0.297	1.049	0.042
BDCA2 CD86 R848	1.007	0.726	1.018	0.394	1.014	0.415	1.099	0.245
BDCA2 CD86 CpGa	0.984	0.380	0.996	0.810	1.019	0.349	1.060	0.040
BDCA2 CD86 mix	0.999	0.962	1.008	0.474	1.013	0.397	1.028	0.081

Red when *p*-value<0.05; w/o stim: without stimulation

Supplementary Table 7: Impact of intra-cellular cytokine production by circulating or tumor-infiltrating DCs after TLR stimulation on clinical outcome (Log-rank test analysis).

Log rank (*p*-values)	Patient blood				Patient tumor infiltrate			
	PFS diagnosis	OS diagnosis	PFS sampling	OS sampling	PFS diagnosis	OS diagnosis	PFS sampling	OS sampling
BDCA1 TNFα w/o stim	0.818	0.314	0.512	0.064	0.549	0.563	0.645	0.674
BDCA1 TNFα polyIC	0.530	0.846	0.702	0.400	0.253	0.542	0.021	0.510
BDCA1 TNFα R848	0.318	0.521	0.538	0.113	0.372	0.909	0.022	0.872
BDCA1 TNFα mix	0.518	0.847	0.797	0.368	0.347	0.301	0.007	0.870
BDCA1 IL-12 p40/p70 w/o stim	0.040	0.227	0.501	0.505	0.945	0.095	0.427	0.093
BDCA1 IL-12 p40/p70 polyIC	0.002	0.028	0.153	0.176	0.919	0.046	0.449	0.233
BDCA1 IL-12 p40/p70 R848	0.318	0.290	0.469	0.653	0.051	0.779	0.212	0.779
BDCA1 IL-12 p40/p70 mix	0.264	0.609	0.402	0.100	0.353	0.215	0.144	0.835
BDCA2 IFNα w/o stim	0.439	0.618	0.942	0.882	0.396	0.542	0.079	0.699
BDCA2 IFNα R848	0.963	0.420	0.839	0.709	0.195	0.886	0.025	0.492
BDCA2 IFNα CpGa	0.799	0.919	0.554	0.599	0.520	0.596	0.391	0.254
BDCA2 IFNα mix	0.304	0.418	0.177	0.122	0.806	0.680	0.306	0.203
BDCA2 TNFα w/o stim	0.245	0.943	0.378	0.530	0.390	0.600	0.180	0.652
BDCA2 TNFα R848	0.053	0.028	0.774	0.176	0.452	0.716	0.157	0.261
BDCA2 TNFα CpGa	0.135	0.031	0.278	0.182	0.962	0.554	0.025	0.373
BDCA2 TNFα mix	0.131	0.443	0.228	0.056	0.032	0.826	0.296	0.441
BDCA3 IFNλ1 w/o stim	0.212	0.017	0.362	0.085	0.156	0.404	0.902	0.413
BDCA3 IFNλ1 polyIC	0.530	0.846	0.702	0.144	0.505	0.311	0.030	0.960
BDCA3 IFNλ1 mix	0.423	0.664	0.549	0.970	0.920	0.874	0.079	0.753
BDCA3 TNFα w/o stim	0.818	0.214	0.624	0.461	0.811	0.392	0.110	0.791
BDCA3 TNFα polyIC	0.696	0.536	0.992	0.604	0.253	0.542	0.021	0.510
BDCA3 TNFα mix	0.845	0.996	0.650	0.399	0.400	0.240	0.389	0.157

Red when *p*-value<0.05; w/o stim:without stimulation

Supplementary Table 8: Impact of intra-cellular cytokine production by circulating or tumor-infiltrating DCs after TLR stimulation on clinical outcome (Cox regression analysis).

COX regression (HR, *p*-values)	Patient blood								Patient tumor infiltrate							
	PFS diagnosis		OS diagnosis		PFS sampling		OS sampling		PFS diagnosis		OS diagnosis		PFS sampling		OS sampling	
	HR	*p*-value	HR	*p*-value	HR	*p*-value	HR	*p*-value	HR	*p*-value	HR	*p*-value	HR	*p*-value	HR	*p*-value
BDCA1 TNFα w/o stim	1.451	0.233	0.992	0.989	0.949	0.900	0.620	0.376	1.000	0.995	1.061	0.309	0.950	0.446	1.013	0.782
BDCA1 TNFα polyIC	0.988	0.872	0.995	0.958	0.969	0.749	0.819	0.206	0.908	0.043	0.991	0.779	0.896	0.076	0.973	0.545
BDCA1 TNFα R848	0.976	0.544	0.982	0.690	0.983	0.682	0.947	0.172	0.984	0.525	0.999	0.975	0.938	0.231	1.010	0.672
BDCA1 TNFα mix	0.987	0.563	1.001	0.982	0.988	0.633	0.963	0.175	0.988	0.475	1.020	0.274	0.949	0.174	1.005	0.760
BDCA1 IL-12 p40/p70 w/o stim	0.848	0.048	0.819	0.101	0.953	0.585	0.909	0.326	0.980	0.488	1.024	0.477	1.031	0.460	1.007	0.802
BDCA1 IL-12 p40/p70 polyIC	0.747	0.024	0.811	0.167	0.872	0.202	0.852	0.178	0.955	0.239	1.041	0.239	1.052	0.477	1.016	0.584
BDCA1 IL-12 p40/p70 R848	0.924	0.130	0.964	0.464	1.000	0.990	0.980	0.618	0.947	0.125	0.998	0.942	0.980	0.608	1.000	0.987
BDCA1 IL-12 p40/p70 mix	0.922	0.122	0.954	0.398	0.983	0.709	0.941	0.231	0.955	0.115	1.020	0.479	0.961	0.194	0.981	0.512
BDCA2 IFNα w/o stim	0.844	0.767	1.105	0.869	0.889	0.840	0.897	0.872	1.056	0.617	0.946	0.611	0.916	0.489	0.993	0.938
BDCA2 IFNα R848	0.877	0.568	0.819	0.602	0.805	0.465	0.545	0.228	0.960	0.262	0.970	0.426	0.957	0.357	1.022	0.633
BDCA2 IFNα CpGa	0.632	0.639	0.141	0.666	0.632	0.675	0.005	0.483	1.046	0.537	1.094	0.297	0.950	0.674	1.084	0.348
BDCA2 IFNα mix	0.876	0.439	0.861	0.506	0.851	0.405	0.750	0.261	0.966	0.180	0.970	0.349	0.962	0.137	0.989	0.605
BDCA2 TNFα w/o stim	4.574	0.225	4.637	0.403	4.240	0.231	1.688	0.638	0.894	0.604	1.022	0.911	1.211	0.545	1.136	0.564
BDCA2 TNFα R848	0.938	0.113	0.930	0.150	0.965	0.340	0.929	0.100	0.995	0.803	1.005	0.870	0.951	0.270	1.111	0.102
BDCA2 TNFα CpGa	2.637	0.106	7.208	0.113	2.440	0.139	2.193	0.118	1.009	0.806	1.029	0.453	1.005	0.901	1.135	0.161
BDCA2 TNFα mix	0.942	0.145	0.946	0.241	0.964	0.374	0.929	0.127	0.988	0.392	1.002	0.958	0.974	0.383	1.025	0.354
BDCA3 IFNλ1 w/o stim	0.598	0.157	0.000	0.997	0.798	0.324	0.706	0.187	1.025	0.288	0.983	0.549	0.980	0.484	0.997	0.890
BDCA3 IFNλ1 polyIC	0.911	0.138	0.950	0.387	0.947	0.291	0.864	0.086	0.984	0.292	0.952	0.224	0.844	0.041	0.973	0.285
BDCA3 IFNλ1 mix	1.023	0.622	1.035	0.555	0.950	0.439	0.965	0.569	1.002	0.850	0.992	0.662	0.954	0.037	0.975	0.180
BDCA3 TNFα w/o stim	1.018	0.751	0.950	0.564	1.001	0.983	0.987	0.849	0.983	0.336	0.986	0.582	0.934	0.209	0.991	0.792
BDCA3 TNFα polyIC	1.031	0.387	1.044	0.201	1.003	0.933	0.993	0.823	0.966	0.158	0.982	0.434	0.913	0.083	0.975	0.338
BDCA3 TNFα mix	0.989	0.789	1.008	0.874	0.895	0.061	0.908	0.049	0.994	0.566	1.008	0.664	0.990	0.724	1.015	0.478

Red when *p*-value<0.05; w/o stim:without stimulation

Supplementary Table 9: Impact of secreted cytokines present in circulating blood or tumor infiltrate after TLR stimulation on clinical outcome (Log-rank test analysis).

Log-rank (*p*-values)	Patient blood				Patient tumor infiltrate			
	PFS diagnosis	OS diagnosis	PFS sampling	OS sampling	PFS diagnosis	OS diagnosis	PFS sampling	OS sampling
IFNα w/o stim	0.244	0.016	0.477	0.149	0.053	0.006	0.417	0.887
IFNα polyIC	0.317	0.171	0.870	0.607	0.849	0.411	0.640	0.282
IFNα R848	0.809	0.702	0.383	0.721	0.695	0.018	0.299	0.923
IFNα CpGa	0.246	0.450	0.867	0.954	0.754	0.127	0.207	0.309
IFNα mix	0.480	0.039	0.774	0.310	0.594	0.752	0.825	0.066
IFNβ w/o stim	0.480	0.265	0.311	0.990	0.598	0.142	0.253	0.436
IFNβ polyIC	0.317	0.949	0.619	0.395	0.544	0.431	0.955	0.847
IFNβ R848	0.589	0.226	0.383	0.074	0.695	0.018	0.299	0.923
IFNβ CpGa	0.246	0.157	0.867	0.654	0.455	0.888	0.774	0.646
IFNβ mix	0.480	0.039	0.774	0.310	0.531	0.769	0.191	0.163
IL-12p70 w/o stim	0.773	0.417	0.524	0.717	0.475	0.704	0.724	0.741
IL-12p70 polyIC	0.589	0.134	0.508	0.599	0.199	0.283	0.821	0.924
IL-12p70 R848	0.809	0.702	0.508	0.176	0.695	0.018	0.299	0.923
IL-12p70 CpGa	0.170	0.281	0.994	0.897	0.251	0.920	0.946	0.171
IL-12p70 mix	0.480	0.048	0.774	0.243	0.363	0.510	0.729	0.233
IFNλ1 w/o stim	0.753	0.332	0.919	0.856	0.752	0.158	0.751	0.647
IFNλ1 polyIC	0.809	0.949	0.383	0.599	0.199	0.283	0.821	0.924
IFNλ1 R848	0.589	0.226	0.383	0.093	0.558	0.984	0.063	0.438
IFNλ1 CpGa	0.246	0.450	0.867	0.954	0.025	0.269	0.110	0.781
IFNλ1 mix	0.773	0.058	0.919	0.404	0.514	0.231	0.316	0.362
IFNλ2 w/o stim	0.773	0.417	0.524	0.717	0.825	0.802	0.945	0.500
IFNλ2 polyIC	0.499	0.824	0.722	0.700	0.655	0.806	0.766	0.506
IFNλ2 R848	0.809	0.949	0.383	0.528	0.558	0.302	0.774	0.358
IFNλ2 CpGa	0.246	0.157	0.867	0.654	0.359	0.269	0.786	0.171
IFNλ2 mix	0.773	0.058	0.919	0.404	0.297	0.711	0.900	0.787

Red when *p*-value<0.05; w/o stim:without stimulation

Supplementary Table 10: Impact of secreted cytokines present in circulating blood or tumor infiltrate after TLR stimulation on clinical outcome (Cox regression analysis).

COX regression (HR, *p*-values)	Patient blood								Patient tumor infiltrate							
	PFS diagnosis		OS diagnosis		PFS sampling		OS sampling		PFS diagnosis		OS diagnosis		PFS sampling		OS sampling	
	HR	*p*-value	HR	*p*-value	HR	*p*-value	HR	*p*-value	HR	*p*-value	HR	*p*-value	HR	*p*-value	HR	*p*-value
IFNα w/o stim	0.999	0.876	0.981	0.191	1.000	0.967	0.991	0.353	1.001	0.055	1.002	0.007	1.000	0.464	1.001	0.095
IFNα polyIC	0.999	0.273	0.999	0.403	1.000	0.412	1.000	0.619	1.000	0.830	1.001	0.120	1.000	0.622	1.001	0.286
IFNα R848	1.001	0.225	1.000	0.918	1.001	0.559	0.999	0.763	1.000	0.595	1.000	0.973	1.000	0.434	1.000	0.251
IFNα CpGa	1.000	0.265	1.000	0.319	1.000	0.674	1.000	0.736	1.000	0.589	1.000	0.369	1.000	0.794	1.000	0.131
IFNα mix	0.999	0.343	0.998	0.100	1.000	0.716	0.999	0.326	1.000	0.597	1.000	0.956	1.000	0.807	1.000	0.067
IFNβ w/o stim	0.994	0.532	0.977	0.276	0.987	0.415	0.994	0.634	1.001	0.467	1.002	0.309	1.003	0.152	1.002	0.326
IFNβ polyIC	0.997	0.250	0.998	0.422	1.000	0.924	1.000	0.702	1.000	0.977	1.000	0.645	1.000	0.629	1.002	0.332
IFNβ R848	1.004	0.374	0.988	0.571	1.002	0.656	0.983	0.319	1.000	0.555	1.000	0.932	1.000	0.393	1.000	0.239
IFNβ CpGa	0.999	0.282	0.997	0.221	1.000	0.846	1.000	0.671	1.000	0.869	1.000	0.756	1.000	0.472	1.000	0.141
IFNβ mix	0.995	0.161	0.983	0.086	1.000	0.956	0.998	0.509	1.000	0.905	1.000	0.621	1.000	0.822	1.000	0.911
IL-12p70 w/o stim	1.017	0.887	0.969	0.819	0.972	0.832	1.107	0.447	0.996	0.830	1.007	0.681	1.039	0.142	0.998	0.926
IL-12p70 polyIC	0.994	0.678	1.001	0.954	0.986	0.480	0.993	0.710	1.000	0.997	1.001	0.965	0.980	0.220	0.994	0.709
IL-12p70 R848	1.019	0.283	0.960	0.749	1.013	0.439	0.969	0.622	1.028	0.273	0.979	0.434	1.066	0.173	0.999	0.966
IL-12p70 CpGa	1.163	0.274	1.086	0.676	1.110	0.387	1.010	0.927	0.946	0.330	1.024	0.684	1.039	0.573	1.138	0.115
IL-12p70 mix	1.002	0.839	0.852	0.065	1.008	0.484	0.965	0.334	0.993	0.232	1.001	0.799	1.000	0.968	1.012	0.075
IFNλ1 w/o stim	1.000	0.961	0.993	0.331	1.001	0.909	1.002	0.745	1.000	0.664	1.001	0.491	1.002	0.199	1.000	0.723
IFNλ1 polyIC	1.002	0.705	1.000	0.982	1.005	0.320	0.999	0.889	1.002	0.112	1.000	0.692	1.001	0.592	0.999	0.442
IFNλ1 R848	0.998	0.885	1.009	0.657	1.023	0.228	1.039	0.105	1.004	0.107	0.999	0.715	1.285	0.998	0.999	0.527
IFNλ1 CpGa	0.996	0.535	0.997	0.609	1.000	0.993	1.000	0.909	1.016	0.069	1.001	0.756	1.004	0.184	0.999	0.714
IFNλ1 mix	1.001	0.766	0.994	0.217	1.003	0.541	0.998	0.731	0.999	0.417	1.001	0.277	1.001	0.595	1.001	0.379
IFNλ2 w/o stim	0.998	0.873	0.990	0.519	0.992	0.573	1.003	0.801	1.003	0.197	0.999	0.719	0.999	0.732	0.995	0.147
IFNλ2 polyIC	0.999	0.261	0.999	0.251	1.001	0.459	1.000	0.894	1.000	0.419	1.000	0.476	1.000	0.927	1.000	0.470
IFNλ2 R848	1.002	0.704	0.987	0.640	1.000	0.987	0.976	0.450	1.000	0.552	1.000	0.862	1.000	0.414	1.001	0.220
IFNλ2 CpGa	0.998	0.269	0.996	0.300	1.000	0.881	0.999	0.681	1.000	0.584	1.000	0.739	1.000	0.811	1.001	0.124
IFNλ2 mix	0.999	0.301	0.986	0.121	1.001	0.528	0.997	0.179	1.000	0.974	1.000	0.787	1.000	0.982	1.000	0.893

Red when *p*-value<0.05; w/o stim:without stimulation
